# Improving the Immunogenicity of Native-like HIV-1 Envelope Trimers by Hyperstabilization

**DOI:** 10.1016/j.celrep.2017.07.077

**Published:** 2017-08-23

**Authors:** Alba Torrents de la Peña, Jean-Philippe Julien, Steven W. de Taeye, Fernando Garces, Miklos Guttman, Gabriel Ozorowski, Laura K. Pritchard, Anna-Janina Behrens, Eden P. Go, Judith A. Burger, Edith E. Schermer, Kwinten Sliepen, Thomas J. Ketas, Pavel Pugach, Anila Yasmeen, Christopher A. Cottrell, Jonathan L. Torres, Charlotte D. Vavourakis, Marit J. van Gils, Celia LaBranche, David C. Montefiori, Heather Desaire, Max Crispin, Per Johan Klasse, Kelly K. Lee, John P. Moore, Andrew B. Ward, Ian A. Wilson, Rogier W. Sanders

**Affiliations:** 1 Department of Medical Microbiology, Academic Medical Center, University of Amsterdam, Amsterdam 1105 AZ, the Netherlands; 2 Department of Integrative Structural and Computational Biology, Scripps CHAVI-ID, IAVI Neutralizing Antibody Center and Collaboration for AIDS Vaccine Discovery (CAVD), The Scripps Research Institute, La Jolla, CA 92037, USA; 3 Department of Medicinal Chemistry, University of Washington, Seattle, WA 98195, USA; 4 Oxford Glycobiology Institute, Department of Biochemistry, University of Oxford, Oxford OX1 3QU, UK; 5 Department of Chemistry, University of Kansas, Lawrence, KS 66047, USA; 6 Department of Microbiology and Immunology, Weill Medical College of Cornell University, New York, NY 10021, USA; 7 Microbial Systems Ecology, Department of Freshwater and Marine Ecology, Institute for Biodiversity and Ecosystem Dynamics, University of Amsterdam, Amsterdam 1098 XH, the Netherlands; 8 Department of Surgery, Duke University Medical Center, Durham, NC 27710, USA; 9 The Skaggs Institute for Chemical Biology, The Scripps Research Institute, La Jolla, CA 92037, USA

## Abstract

The production of native-like recombinant versions of the HIV-1 envelope glycoprotein (Env) trimer requires overcoming the natural flexibility and instability of the complex. The engineered BG505 SOSIP.664 trimer mimics the structure and antigenicity of native Env. Here, we describe how the introduction of new disulfide bonds between the glycoprotein (gp)120 and gp41 subunits of SOSIP trimers of the BG505 and other genotypes improves their stability and antigenicity, reduces their conformational flexibility, and helps maintain them in the unliganded conformation. The resulting next-generation SOSIP.v5 trimers induce strong autologous tier-2 neutralizing antibody (NAb) responses in rabbits. In addition, the BG505 SOSIP.v6 trimers induced weak heterologous NAb responses against a subset of tier-2 viruses that were not elicited by the prototype BG505 SOSIP.664. These stabilization methods can be applied to trimers from multiple genotypes as components of multivalent vaccines aimed at inducing broadly NAbs (bNAbs).

## Highlights

• The stability of HIV-1 envelope trimers can be increased by structure-based design
• Design improvements are applicable to envelope trimers from different HIV-1 isolates
• Immunization of stabilized HIV-1 envelope trimers is improved

## Graphical Abstract

**Figure:**
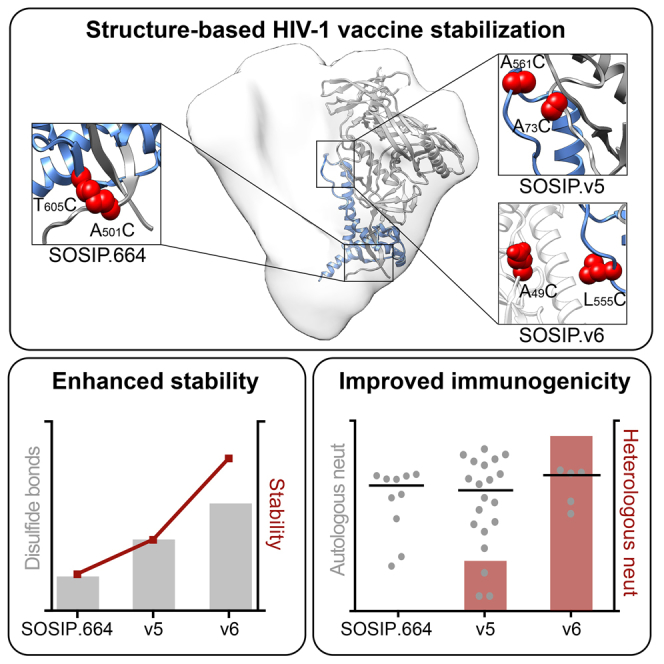

## Teaser

Native-like HIV-1 envelope trimers are a platform for efforts to induce broadly neutralizing antibodies. Torrents de la Peña et al. design HIV-1 envelope trimers with enhanced stability and reduced flexibility. These modified trimers improve the induction of neutralizing antibodies and provide new opportunities toward elicitation of broadly neutralizing antibodies.

## Introduction

Despite many attempts, no experimental vaccine has induced strongly protective immunity against HIV-1 infection. One approach to this problem is the generation of an envelope glycoprotein (Env)-based vaccine that induces broadly neutralizing antibodies (bNAbs) (van Gils and Sanders, 2013). A major obstacle to creating such a vaccine is the instability of the Env trimer, which for many years hindered the generation of recombinant, soluble proteins that adequately mimicked the functional Env trimer on virions.

We have described a soluble, recombinant Env trimer, BG505 SOSIP.664, that is stabilized by a disulfide bond between glycoprotein (gp)120 and gp41 and an Ile-to-Pro substitution at position 559 in gp41 (Binley et al., 2000, Sanders et al., 2002, Sanders et al., 2013). Several BG505 trimer structures, determined by X-ray crystallography and cryoelectron microscopy (cryo-EM), have provided new insights into the architecture and function of HIV-1 Env (Garces et al., 2015, Julien et al., 2013a, Kwon et al., 2015, Lee et al., 2016, Lyumkis et al., 2013, Pancera et al., 2014, Sanders and Moore, 2014, Scharf et al., 2015, Stewart-Jones et al., 2016). Negative-stain electron microscopy (EM) and cryo-EM studies show that it closely resembles the native, membrane-associated trimer at the structural level (Lee et al., 2016, Lyumkis et al., 2013, Sanders et al., 2013). The BG505 SOSIP.664 trimer, and others of the same design based on different genotypes, displays the epitopes for most bNAbs and few non-NAbs (Derking et al., 2015, Huang et al., 2014, Julien et al., 2013b, Sanders et al., 2013). In immunogenicity studies in rabbits, the BG505, AMC008, B41, CZA97, and DU422 SOSIP.664 trimers induced NAbs against the corresponding autologous viruses (Cheng et al., 2015, Klasse et al., 2016, Sanders et al., 2015, de Taeye et al., 2015).

Although SOSIP.664 trimers are stable enough to produce and purify, we hypothesized that their performance as immunogens could be improved by reducing their conformational flexibility and the consequent exposure of immunodominant, potentially distractive non-NAb epitopes (de Taeye et al., 2015).

The increasingly high-resolution structures of SOSIP trimers greatly facilitate the design of stabilization strategies. BG505 SOSIP.664 trimers have already been further stabilized by adding an intra-gp120 disulfide bond linking residues 201 and 433, which fixes the bridging sheet in its ground state and thereby reduces the exposure of non-NAb epitopes (Guenaga et al., 2015, Kwon et al., 2015). Combining two different substitutions in the gp120 subunit, E64K or H66R plus A316W, defined as SOSIP.v4, increases the stability of trimers of various genotypes, reduces the exposure of non-NAb CD4i and V3 epitopes (i.e., 17b, 19b, and 14e), and decreases the induction of V3-directed non-NAbs in immunized rabbits (de Taeye et al., 2015). Forming a complex with the quaternary-structure-dependent bNAb, PGT145, can also improve trimer stability (Cheng et al., 2015). Moreover, by comparing the BG505 sequence with other sequences that form stable trimers poorly, several substitutions were identified that increase the stability of JR-FL and 16055 SOSIP.664 trimers (Guenaga et al., 2015). Steichen et al. (2016) used mammalian cell surface display to design stabilized trimers that showed an improvement in trimer expression and reduction of V3 exposure. A considerable improvement in yield was achieved by computationally redesigning the N-terminal residues of heptad region 1 (HR1) of Env SOSIP trimers (Kong et al., 2016). Finally, we have also introduced a disulfide bond between gp120 residue-49 of one protomer and gp41 residue-555 of a second protomer to increase thermostability of the trimer. Strong precedents underpin this approach as disulfide bonds play a well-established role in protein stability (Camacho and Thirumalai, 1995, Creighton, 1988). For example, disulfide bonds can be up to 17-fold more abundant in proteins from thermophilic archea and bacteria, compared to mesophiles, and the number of disulfides correlates with their maximum growth temperature (Liszka et al., 2012). Furthermore, disulfide bonds have been used to stabilize the immunogens from respiratory syncytial virus (RSV) and influenza virus (Lee et al., 2016, McLellan et al., 2013).

Here, we describe the structure-guided introduction of additional disulfide bonds between gp120 and gp41 that further stabilize the resulting SOSIP.v5 trimers in their unliganded, closed state. Furthermore, we have combined these intra-protomer bonds with recently described inter-protomer bonds to create hyperstable SOSIP.v6 trimers. When BG505 SOSIP.v6 trimers were tested as immunogens in rabbits, they induced strong autologous responses, as well as weak, but consistent, NAbs against a subset of heterologous tier-2 viruses.

## Results

### Addition of a Second Disulfide Bond between gp120 and gp41

**Figure 1:**
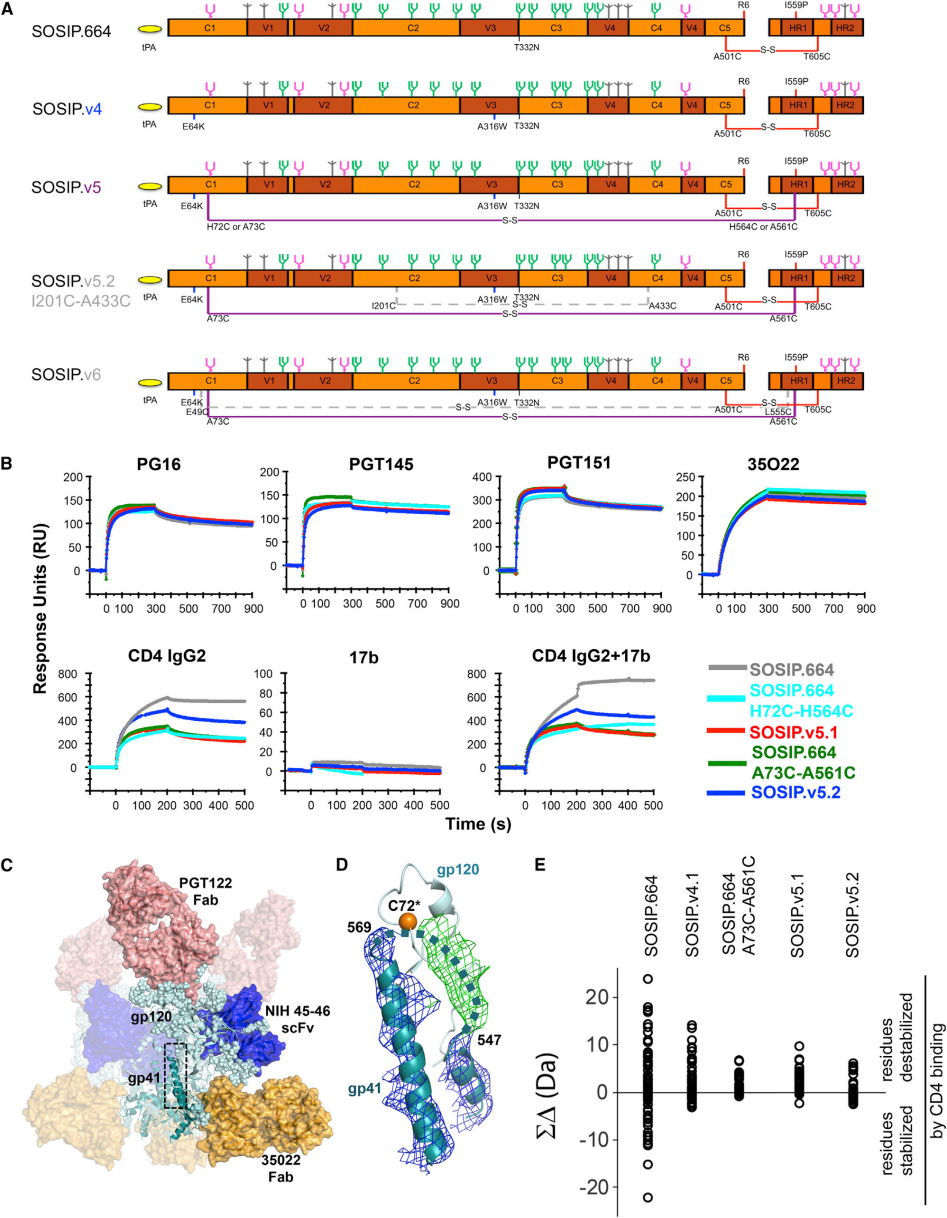
Design, Antigenicity, Structure, and Conformational Dynamics of BG505 SOSIP.v5 Trimers Containing Novel Disulfide Bonds between gp120 and gp41 (A) Linear schematic of the BG505 SOSIP.664, SOSIP.v4, SOSIP.v5, SOSIP.v5.2 I201C-A433C, and SOSIP.v6 constructs. Modifications that create the SOSIP.664 construct are indicated in red (Sanders et al., 2013). The E64K and A316W substitutions added to make the SOSIP.v4 construct are colored blue (de Taeye et al., 2015). The engineered disulfide bonds in SOSIP.v5 are shown in purple. There are two variants of the SOSIP.v5 construct: the SOSIP.v5.1 has a disulfide bond between H72C and H564C and the SOSIP.v5.2 has a disulfide bond between A73C and A561C. The I201C-A433C disulfide bond, previously described by Kwon et al. (2015), was introduced into the SOSIP.v5.2 background. The E49C-L555C disulfide bond was introduced into the SOSIP.v5.2 construct. The resulting constructs are designated SOSIP.v5.2 I201C-A433C and SOSIP.v6, respectively. (B) SPR analysis of the binding of bNAbs PG16, PGT145, PGT151, and 35O22 to quaternary epitopes (upper panel), and of CD4-IgG2 and non-NAb 17b CD4i (±prior addition of CD4-IgG2) (lower panel), to the indicated BG505 trimer variants. (C) Crystal structure of the quaternary complex of BG505 SOSIP.664 H72C-H564C trimer (cyan) in complex with PGT122 Fab (pink), 35O22 Fab (orange), and NIH45-46 scFv (deep blue). The three different antibody Fab or scFv fragments are represented as colored surfaces, and the trimer N-linked glycans as cyan spheres. One of the three protomers is highlighted for clarity, and the gp41 central HR1 helix location proximal to the engineered H72C-H564C disulfide is marked with a rectangle. (D) Detail of the interaction between gp41 HR1 and gp120. A 2Fo-Fc composite omit map contoured at 1.0σ around gp41 HR1 (blue mesh) reveals ordered electron density (green mesh) for gp41 HR1_N_ residues 547–569 (dotted lines), implying that the H72C-H564C disulfide bond helps stabilize this region of the trimer in a ground-state prefusion conformation. The figure was rendered using PyMOL. (E) Differences in hydrogen/deuterium (H/D) exchange rates between the unliganded and CD4-bound forms of the indicated SOSIP variants. The difference between the two states is the difference obtained by subtracting the deuterons exchanged in the unliganded condition from the deuterons exchanged in the CD4-bound forms. While positive values indicate that the residues were destabilized in the presence of 2D-sCD4, negative values indicate stabilization. The net difference in H/D exchange as a sum of all time points is plotted for each observable peptide. Only differences that were outside the error range were included in the summation process. The same set of peptides was used for each trimer construct. The individual exchange plots are shown in Data S1A.

To reduce the flexibility and increase the stability of BG505 SOSIP.664 trimers, we designed, screened, and produced variants containing an additional engineered intra-protomer disulfide bond; i.e., between the gp120 and gp41 subunits. Since the disulfide bond between residue 501 of gp120 and residue 605 of gp41 (i.e., the SOS bond), used to create SOSIP trimers, is located near the base of the trimer (Binley et al., 2000, Garces et al., 2015, Pancera et al., 2014), we sought more central locations for a second intra-protomer bond. Initially, new disulfide bonds were evaluated in the absence of the SOS bond, and a few promising candidates were subsequently chosen to make double disulfide-bond variants (Figures S1 and S2). Based on favorable biochemical and antigenic properties, we selected two such variants, designated SOSIP.664 H72C-H564C and SOSIP.664 A73C-A561C, for further analyses (Figures S1 and S2). To provide additional stability, we also introduced two point substitutions: A316W to improve hydrophobic packing of V3 residues and prevent sporadic, unwanted V3 exposure from its hidden location below V1V2 and E64K to impede the occasional spontaneous and reversible sampling of the CD4-bound conformation (Figure 1A; de Taeye et al., 2015). SOSIP.664 trimers containing the E64K and A316W substitutions and either an H72C-H564C or A73C-A561C disulfide bond are referred to as SOSIP.v5.1 or SOSIP.v5.2 variants, respectively (Figure 1A; see also Table S1).

### Biochemical and Biophysical Properties of Stabilized BG505 SOSIP.v5 Trimers

The two variants of BG505 SOSIP.v5 trimers were expressed in 293F cells and purified via PGT145-affinity chromatography, as previously described (de Taeye et al., 2015). Non-reducing SDS-PAGE analysis showed that the SOSIP.v5 proteins migrated more slowly than their SOSIP.664 counterparts (Figures S3A and S3B), consistent with a decrease in SDS uptake when a protein becomes more compact. We used tandem mass spectrometry (MS/MS) to confirm that, in addition to the ten canonical disulfide bonds and the SOS bond, the new bond was also formed in both SOSIP.v5 variants (Table S5).

PGT145-purified, native-like BG505 SOSIP.664 trimers have a more open configuration on average compared to those purified by 2G12 columns, typically ∼30% versus >95% closed (de Taeye et al., 2015). The addition of the H72C-H564C or A73C-A561C disulfides further increases to 85% closed for SOSIP.v5.1 and 90% for SOSIP.v5.2 trimers (Table 1 and Figure S3C). Dynamic light scattering (DLS) studies support the conclusion that both SOSIP.v5 trimers are more compact (see Table S2 and Figure S3D for these and other biophysical analyses, including small angle X-ray scattering [SAXS]).

**Table 1:** Biophysical Characterization of Stabilized SOSIP Trimers from BG505, AMC008, B41, and ZM197M Isolates

New Substitutions Added to SOSIP.664	SOSIP Version^a^	Yield (mg/L) (≈)	Morphology (NS-EM)		Thermostability (DSC)^b^		Glycan Composition (HILIC-UPLC)^c^		
			Native-like (%) (>)	Closed Native-like (%)	*T*_m_ (°C)	Δ*T*_m_ (°C)	Man_8_	Man_9_	Oligo-mannose
**BG505**									

–	SOSIP.664	2.0	98	35	67.6	–	18	23	64
H72C-H564C		2.0	98	70	71.9	4.3	21	20	63
A73C-A561C		2.0	98	70	72.5	4.9	ND	ND	ND
E64K A316W	SOSIP.v4.1^d^	2.0	98	70	70.7	3.1	21	23	64
E64K A316W H72C-H564C	SOSIP.v5.1	2.0	98	85	75.0	7.4	21	20	68
E64K A316W A73C-A561C	SOSIP.v5.2	2.0	98	90	75.3	7.7	21	23	64
I201C-A433C^e^	DS-SOSIP	1.0	98	65	74.5	8.9	ND	ND	ND
E64K A316W A73C-A561C I201C-A433C		1.5	98	85	80.7	13.1	23	19	61
E49C-L555C^f^		0.2	ND	ND	75.2	7.6	ND	ND	ND
E64K A316W A73C-A561C E49C-L555C	SOSIP.v6	0.8	98	50	78.7/92.2^g^	11.2/24.6^g^	25	25	69

**AMC008**									

–	SOSIP.664	2.0	98	15	60.2	–	14	34	67
I535M L543N H66R A316W	SOSIP.v4.2^d^	2.0	98	85	64.5	4.5	12	39	69
I535M L543N H66R A316W H72C-H564C	SOSIP.v5.1	1.5	98	100	68.3	8.1	13	33	65
I535M L543N H66R A316W A73C-A561C	SOSIP.v5.2	1.5	98	100	68.5	8.3	14	30	65

**B41**									

–	SOSIP.664	2.0	98	45	58.6	–	15	29	63
L543N E64K A316W	SOSIP.v4.1^d^	2.0	98	55	60.7	2.1	14	32	64
L543N E64K A316W H72C-H564C	SOSIP.v5.1	0,2	98	95	63.0	4.3	ND	ND	ND
L543N E64K A316W A73C-A561C	SOSIP.v5.2	1.5	98	70	64.7	6.1	17	25	64

**ZM197M**									

–	SOSIP.664	0.3	95	15	62.2	–	22	26	70
V535M H66R T316W	SOSIP.v4.2^d^	0.3	90	30	62.6	0.4	21	26	69
V535M H66R T316W H72C-H564C	SOSIP.v5.1	1.0	98	100	69.5	7.3	ND	ND	ND
V535M H66R T316W A73C-A561C	SOSIP.v5.2	1.0	98	100	69.2	7.0	ND	ND	ND

The yields of PGT145-purified trimers are listed, together with the percentages that have a native-like conformation and also in the completely closed form, as determined by negative-stain EM. The 2D class averages were derived using trimers without a C-terminal tag and are shown in Figure S5C. ND, not determined.

a An overview of the modifications made to the stabilized trimer variants is shown in Table S1.

b The *T*_m_ values for each construct were obtained by DSC using a two-state model (Figure S5D). The *T*_m_ values are based on two-state model fitting. *T*_m_ values for His-tagged trimers (BG505) were consistently ∼0.9°C –1.0°C higher than the values for the same trimers without a tag. *T*_m_ values obtained with D7324-tagged trimers (AMC008, B41, and ZM197M) were up to 0.3°C higher than those without a tag.

c Percentages of Man_8_, Man_9_, and total oligomannose glycans are given for each trimer (Figure S5E). The percentage of Man_8_GlcNAc_2_ and Man_9_GlcNAc_2_ glycans, as well as the total percentage of oligomannose glycans are provided for each His/D7324-tagged trimer (Figure S5E).

d NS-EM and glycan composition data for SOSIP v4.1 and SOSIP v4.2 trimers were previously reported in de Taeye et al. (2015).

e Data for this trimer variant were previously described in Kwon et al. (2015) and Guenaga et al. (2015). Experiments were performed with newly purified trimers.

f SOSIP.664 trimer variant was described in Garces et al. (2015). Experiments were performed with newly purified trimers.

g The two values represent the peaks of the two independent unfolding events observed by DSC (see Figure S3E for the raw data).

We then assessed BG505 trimer thermostability. The introduction of either disulfide bond in the SOSIP.v4.1 context again increased trimer thermostability with *T*_m_ values of 75.0°C and 75.3°C for SOSIP.v5.1 and SOSIP.v5.2, respectively, representing increases of 4.3°C and 4.6°C over SOSIP.v4.1 and 7.4°C and 7.7°C over SOSIP.664 (Table 1; Figure S3E).

The glycosylation profiles of the SOSIP.664 H72C-H564C, SOSIP.664 A73C-A561C, and both SOSIP.v5 trimers were all similar to those of SOSIP.664 and SOSIP.v4.1, with oligomannose glycoforms dominating (61%–64%) and, in particular, Man_8_GlcNAc_2_ and Man_9_GlcNAc_2_ glycans (Table 1; Figure S3F). The high density of unprocessed oligomannose glycans on gp120 is a hallmark of native-like Env trimers (Pritchard et al., 2015a, Pritchard et al., 2015b).

### Antigenicity of Stabilized BG505 SOSIP.v5 Trimers

We used a panel of bNAbs to assess the antigenicity of stabilized BG505 trimer variants by ELISA. All of the tested bNAbs bound comparably to the SOSIP.664, SOSIP.v4, and SOSIP.v5 trimers, implying that the changes did not compromise antigenicity (Table 2; Data S1A). Surface plasmon resonance (SPR) studies confirmed that the quaternary-structure-dependent epitopes at the trimer apex (PG16 and PGT145) and at the gp120/gp41 interface (35O22 and PGT151) were fully preserved on both versions of SOSIP.v5 trimers (Figure 1B). SPR analysis showed that the association of CD4-IgG2 was similar between SOSIP.664 and SOSIP.v5, but the dissociation from both SOSIP.v5 variants was markedly faster (Figure 1B), consistent with previous measurements with SOSIP.v4 trimers (de Taeye et al., 2015). These data are also consistent with a report in which stabilization of BG505 SOSIP.664 with an intra-gp120 disulfide bond (DS-SOSIP.664) was shown to increase soluble CD4 (sCD4) dissociation (Kwon et al., 2015).

**Table 2:** Antigenic Characterization of Stabilized Trimers from BG505, AMC008, B41, and ZM197M Isolates

New Substitutions Added to SOSIP.664	SOSIP Version^a^	Broadly Neutralizing Antibodies									Non-neutralizing Antibodies					
		V1V2 Apex			V3-Glycan	OD Glycan	CD4bs	gp120-gp41 Interface			CD4i		V3		CD4bs	CD4bs
		PG9	PG16	PGT145	PGT121	2G12	VRC01	PGT151	35022	3BC315	17b	17b+sCD4	14e	19b	B6	CD4 IgG2
**BG505**																

–	SOSIP.664	0.079	0.054	0.020	0.044	0.014	0.022	0.016	0.026	0.130	0.304	0.077	0.039	0.013	0.143	0.020
H72C-H564C		90	98	76	88	129	118	87	73	89	0	56	72	88	55	80
A73C-A561C		95	158	84	115	119	105	136	93	111	0	43	71	83	59	61
E64K A316W	SOSIP.v4.1^b^	91	93	77	101	104	113	107	71	106	0	15	2	0	13	54
E64K A316W H72C-H564C	SOSIP.v5.1	69	81	94	72	103	106	118	77	95	0	8	2	4	9	35
E64K A316W A73C-A561C	SOSIP.v5.2	77	94	86	79	122	116	131	75	93	0	8	2	5	7	55
E64K A316W A73C-A561C I201C-A433C		76	95	231	88	85	87	135	130	102	0	0	2	9	10	61
E64K A316W A73C-A561C E49C-L555C	SOSIP.v6	112	103	135	58	123	78	130	85	99	0	9	2	6	5	68

**AMC008**																

–	SOSIP.664	ND	1.761	1.300	0.026	0.010	0.061	0.162	0.832	0.050	0.13	0.001	0.002	ND	0.017	0.534
I535M L543N H66R A316W	SOSIP.v4.2^a^	ND	62	125	114	95	102	115	162	116	0	1	6	ND	30	103
I535M L543N H66R A316W H72C-H564C	SOSIP.v5.1	ND	128	134	127	69	102	118	171	107	0	1	12	ND	34	63
I535M L543N H66R A316W A73C-A561C	SOSIP.v5.2	ND	127	130	119	40	118	185	231	95	0	0	10	ND	23	96

**B41**																

–	SOSIP.664	0.077	0.094	0.151	0.053	0.043	0.126	0.152	1.665	0.024	0.839	0.001	0.007	0.003	0.017	0.029
L543N E64K A316W	SOSIP.v4.1^a^	97	104	92	108	102	126	121	115	58	0	1	8	14	20	21
L543N E64K A316W H72C-H564C	SOSIP.v5.1	127	73	59	104	70	125	232	135	44	0	2	1	18	34	9
L543N E64K A316W A73C-A561C	SOSIP.v5.2	113	418	86	116	125	116	221	159	84	0	5	28	16	23	23

**ZM197M**																

–	SOSIP.664	0.263	0.487	2.310	0.192	0.126	0.034	0.142	0.013	0.022	0.10	0.055	0.064	0.637	0.295	0.138
V535M H66R T316W	SOSIP.v4.2^a^	73	93	113	88	95	108	95	85	28	0	42	14	55	59	92
V535M H66R T316W H72C-H564C	SOSIP.v5.1	16	91	352	84	96	125	234	92	28	0	3	12	17	9	13
V535M H66R T316W A73C-A561C	SOSIP.v5.2	30	121	342	87	102	116	267	425	45	0	5	19	36	10	26

Binding of bNAbs and non-NAbs was determined using a Ni-NTA (BG505) or a D7324- (AMC008, B41, and ZM197M) capture ELISA. Half-maximal binding concentrations (half maximal effective concentration [EC_50_], in μg/mL) are shown for SOSIP.664 trimers (underlined). Antibody binding to the various stabilized trimers is expressed as percentage of the binding to SOSIP.664 (EC_50_ is defined as 100%). The values are representative of at least two independent experiments. The ELISA curves from one representative experiment are shown in Data 1B–1E. ND, not determined.

a An overview of the modifications made to the stabilized trimer variants is shown in Table S1.

b The SOSIP v4.1 and SOSIP v4.2 trimer variants were described in de Taeye et al. (2015).

BG505 SOSIP.664 trimers minimally, but detectably, bind 17b, a non-NAb against a CD4-inducible (CD4i) epitope, by ELISA (Sanders et al., 2013). Under the same conditions, neither SOSIP.v5 trimer bound detectably to 17b when CD4-immunoglobulin G (IgG2) was absent, and the extent of CD4i-epitope induction by CD4-IgG2 was less than with SOSIP.664 and SOSIP.v4.1 in ELISA (Table 2; Data S1B). SPR analysis confirmed and extended these results (Figure 1B). Moreover, non-NAb b6 that targets the CD4 binding site (CD4bs) bound less well to the SOSIP.v5 trimers than to SOSIP.664 and SOSIP.v4.1 (Table 2; Data S1B). Some of the reduction in non-NAb epitope exposure in SOSIP.v5 is attributable to the A316W substitution that is present in SOSIP.v4 trimers (de Taeye et al., 2015). However, the new disulfide bonds in the SOSIP.v5 trimers confer additional benefits (Table 2; Data S1B).

Taken together, the antigenicity studies show that the SOSIP.v5 trimers preserve the desired bNAb reactivity profiles of their SOSIP.664 and SOSIP.v4.1 precursors.

### X-Ray Structure of a Stabilized BG505 SOSIP Trimer

To assess the impact of an extra disulfide bond on the trimer structure, we expressed the BG505 SOSIP.664 H72C-H564C trimer in N-acetyl-glucosaminyltransferase I (GnTI)-deficient HEK293S cells and purified it by 2G12-affinity chromatography followed by size exclusion chromatography (SEC). Its crystal structure was determined in complex with bNAbs PGT122 Fab, 35O22 Fab, and NIH45-46 scFv (Figure 1C). Although the crystals only diffracted to 7 Å resolution, the availability of higher-resolution structures of other BG505 SOSIP trimers allowed a clear interpretation of the lower-resolution electron density maps, as exemplified by good refinement statistics (Table S3). The overall architecture of the trimer is indeed preserved upon addition of the 72C-564C inter-subunit disulfide bond (Figure 1C). In addition, bNAbs against three distinct sites of vulnerability (PGT122, N332; 35O22, gp41/gp120 interface; and NIH45-46, CD4bs) interacted with their epitopes in a similar manner to the SOSIP.664 trimer (Figure 1C). Residues 547–569 in the gp41 HR1_N_ region near the 72C substitution in gp120 have defined electron density similar to that in higher-resolution BG505 SOSIP.664 trimer structures (Garces et al., 2015; PDB: 5CEZ) filtered at the same resolution (Figure 1D), indicating that this region is well ordered and native like. Thus, the added H72C-H564C disulfide bond preserves the native-like trimer structure.

### Dynamics of BG505 SOSIP.v5 Trimers

We compared the SOSIP constructs by hydrogen-deuterium exchange (HD-X) to explore the impact of the new trimer-stabilizing changes on protein dynamics and conformational flexibility. The resulting exchange profiles for the BG505 SOSIP.664 A73C-A561C, SOSIP.v5.1 and SOSIP.v5.2 trimers were overall consistent with the SOSIP.664 precursor (Figure S4). Only a few minor changes were observed, which are proximal to the newly introduced disulfide linkages. We used the same method to compare CD4-induced conformational changes. Addition of sCD4 to the SOSIP.664 trimers resulted in less protection in the V2 and V3 loops, but more protection of the CD4bs, gp120 layers 1–3, and gp41 HR1, in agreement with previous studies (Figures 1E and S4) (Guttman et al., 2014). The changes in the various stabilized mutants were greatly diminished or completely abolished (Figures 1E and S4). The SOSIP.v5.2 variant responded least to sCD4, in that the CD4-induced exposure of V2 and V3 was now entirely abrogated (Figure S4). Thus, the extra inter-subunit disulfide bond and the E64K and A316W substitutions are synergistic in preventing the opening of the apex of the SOSIP.v5.2 trimers when sCD4 is present. The data strongly suggest that the BG505 SOSIP.v5.2 trimers are effectively trapped in the closed, ground state.

### Stabilizing SOSIP Trimers from Clades B and C

To assess the generality of the stabilization method, we introduced the H72C-H564C or A73C-A561C disulfide bonds into B41 (clade B) SOSIP.v4.1, AMC008 (clade B), and ZM197M (clade C) SOSIP.v4.2 constructs (Julien et al., 2015, Pugach et al., 2015, de Taeye et al., 2015). The resulting variants were purified and analyzed by SDS-PAGE and blue native (BN)-PAGE analysis. While AMC008 SOSIP.v5 and B41 SOSIP.v5 yielded similar trimer amounts to comparably produced SOSIP.664 and SOSIP.v4 counterparts, the yields for ZM197M were considerably improved (1.0 mg/L for SOSIP.v5.1 and v5.2 versus ∼0.3 mg/L for SOSIP.664 and SOSIP.v4) (Table 1). All of the variously purified SOSIP.v5 trimers were cleaved efficiently (Figures S5A and S5B) and migrated more slowly on non-reducing SDS-PAGE gels (Figure S5A). Negative-stain EM confirmed that the PGT145-purified SOSIP.v5 trimers were again invariably native like and also more likely to be in the closed conformation (Table 1; Figure S5C). As for BG505, oligomannose glycoforms dominated (63%–70%, in particular Man_8_GlcNAc_2_ and Man_9_GlcNAc_2_, Table 1; Figure S5E). The additional disulfide bond improved the thermostability of each trimer construct. For AMC008, the *T*_m_ increased from 60.2°C (SOSIP.664) to 68.3°C–68.5°C (SOSIP.v5.1 and v5.2; Table 1; Figure S5D). Increases in *T*_m_ of between 4°C and 7°C compared to SOSIP.664 were also observed for the various B41 and ZM197M SOSIP.v5.1 and SOSIP.v5.2 trimers (Table 1; Figure S5D). Overall, we conclude that introducing a second inter-subunit disulfide bond confers additional stability on SOSIP.v5 trimers of four different isolates from three different clades.

### Antigenic Properties of B41, AMC008, and ZM197M SOSIP.v5 Trimers

In general, the antigenicity properties of the SOSIP.v5.1 and v5.2 trimers of all three genotypes were again comparable to the corresponding SOSIP.v4 and/or SOSIP.664 trimers, bNAb binding was retained, while non-NAb reactivity with V3 and CD4i epitopes was reduced (Table 2; Data S1C–S1E). CD4-IgG2 binding to B41 and ZM197M SOSIP.v5 variants was reduced compared to SOSIP.v4 (Table 2). An unexpected observation was that introduction of an additional disulfide bond into the ZM197M SOSIP.v5 construct improved binding of the quaternary-structure-dependent bNAb PGT145 and PGT151, compared to SOSIP.664 and SOSIP.v4 trimers (Table 2; Data S1C). We noticed that in contrast to the quaternary-specific PGT145 bNAb, the quaternary preferring PG9 showed a slightly decreased binding for ZM197M SOSIP.v5 compared to SOSIP.664 (Table 2; Data S1C). The introduction of an additional disulfide bond also had a noticeable effect for B41, for which PGT151 binding was negligible for SOSIP.664 trimers, but strong for both SOSIP.v5 versions (Table 2; Data S1D). We can conclude that SOSIP.v5 exhibits a similar or somewhat better antigenic profile compared to SOSIP.664 and SOSIP.v4.1.

### Comparison and Combination with Other Trimer Stabilization Approaches

Two other SOSIP trimer stabilization strategies are outlined in Figure 1A. First, introducing a disulfide bond between residues 201 and 433 (substitutions I201C and A433C) in the gp120 bridging sheet has been reported to increase the thermostability and reduce the conformational flexibility of these BG505 trimers (Kwon et al., 2015). Second, a disulfide bond between gp120 and gp41 of different protomers (substitutions E49C and L555C) also makes BG505 trimers more thermostable (Garces et al., 2015). BG505 SOSIP trimer variants containing either of these disulfide bonds have a reduced exposure of CD4i non-NAb epitopes (Garces et al., 2015, Kwon et al., 2015).

We introduced the above disulfide bonds into BG505 SOSIP.664 or SOSIP.v5.2 constructs and determined the yields, antigenic profiles, and thermal stability of the resulting trimers. The SOSIP.v6 construct contains the E49C-L555C substitutions on the SOSIP.v5.2 background, while the “SOSIP.v5.2 I201C-A433C” construct includes the 201C-433C disulfide bond in the SOSIP.v5.2 background (Figure 1A). The yields of PGT145-purified SOSIP.v5.2 I201C-A433C and SOSIP.v6 trimers (∼1.5 and ∼0.8 mg/L, respectively) were lower than for SOSIP.664 and SOSIP.v5.2 (∼2.0 mg/L) (Table 1). Non-reducing SDS-PAGE analysis showed that SOSIP.v6 proteins migrated as a trimer, which is consistent with the formation of inter-protomer disulfide bonds (Table S3A). However, two subpopulations were visible on the gels. Based on a comparison with the migration patterns of various control proteins, we propose that the slower migrating variant, ∼60% of the total, contains all three engineered disulfide bonds (501C-605C, 73C-561C, and 49C-555C), while the faster migrating species lacks the 73C-561C bond whose formation may be restricted by the presence of the 49C-555C bond in a subpopulation of trimers (Figure S3A).

The antigenic profile of these more stable trimers, SOSIP.v5.2 I201C-A433C and SOSIP.v6, are very similar to their less stable counterparts. The quaternary antibodies PGT151 and PGT145 bound slightly more strongly to the BG505 SOSIP.v5.2 I201C-A433C and SOSIP.v6 trimers than to their precursors, while the binding of non-NAbs to V3 and CD4i epitopes remained very low (Table 2; Data S1B). Both new trimers resembled their precursors in containing mostly oligomannose glycoforms (Table 1; Figure S3F).

The new disulfide bonds further increased BG505 trimer thermostability. Adding the 201C-433C bond to the SOSIP.v5.2 construct increased the *T*_m_ from 75.3°C to 80.7°C. The SOSIP.v6 trimer was particularly thermostable, the *T*_m_ of the majority subpopulation was 92.2°C, while a smaller unfolding event was detected at 78.8°C, which we propose reflects the minority of trimers in which the 73C-561C linkage is not formed (Table 1; Figure S3E).

### Immunogenicity of Stabilized BG505 SOSIP Trimers in Rabbits

**Figure 2:**
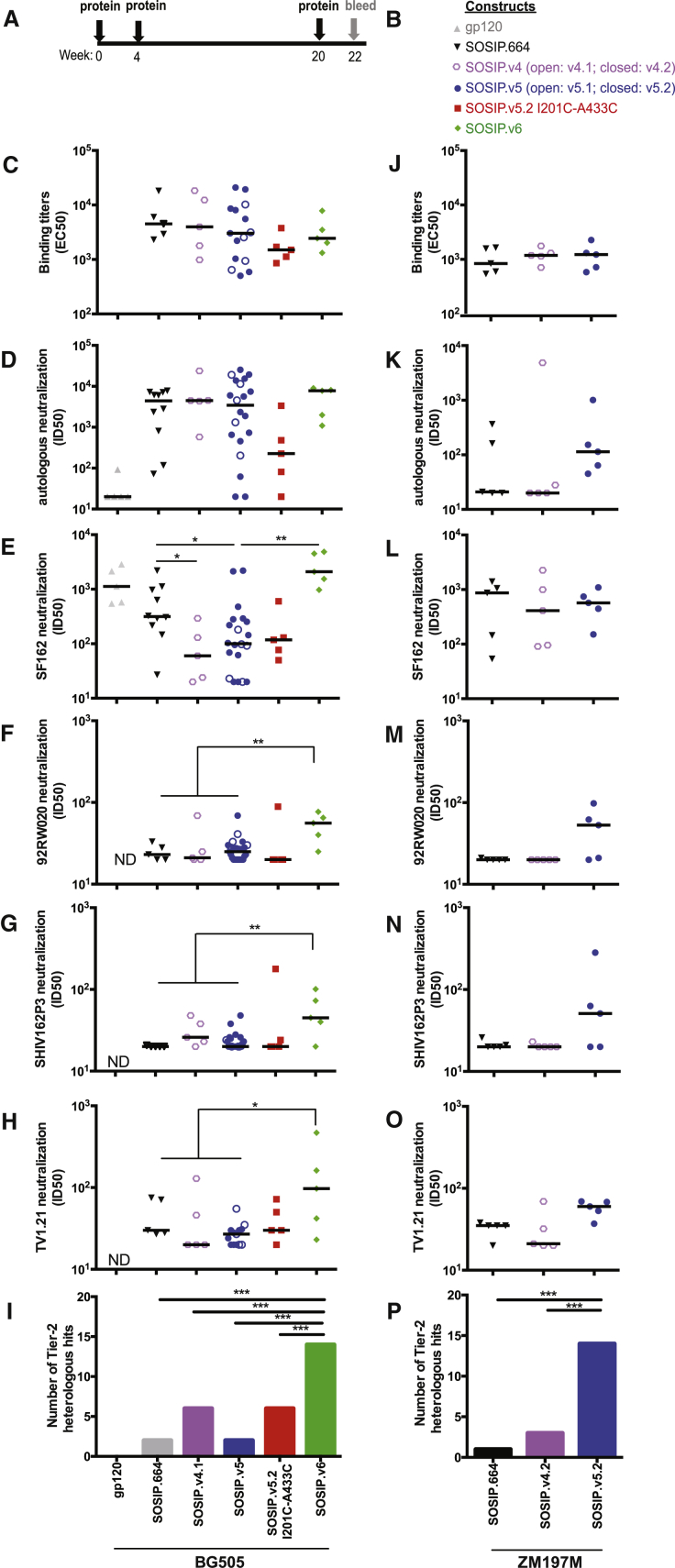
Immunogenicity of BG505 and ZM197M SOSIP.v5 Trimers in Rabbits (A) Schematic representation of immunization schedule. (B) Color coding for the immunogens tested. (C–I) Shows data for BG505-based immunogens. (J –P) Shows data for ZM197M. (C and J) Midpoint antibody binding titers (ED_50_) as measured by D7324-capture ELISA for the trimer variants indicated on the x axis at the bottom of the figure, and by the color-coding scheme outlined in (B). (D–H and J–O) Neutralization of HIV-1 viruses in the TZM-bl assay by sera from animals immunized with BG505 or ZM197M trimer variants. The plots show ID_50_ values, the serum dilution at which infectivity is inhibited by 50%. (D–H and K–O) Autologous viruses (D and K); SF162 heterologous tier-1A (E and L); 92RW020 heterologous tier-2 virus (F and M); SHIV162P3 heterologous tier-2 virus (G and N); and TV1.21 heterologous tier-2 virus (H and O). (I and P) The cumulative numbers of heterologous viruses neutralized with an ID_50_ >40 by the five sera from each group are shown. In the particular case of the BG505 SOSIP.v5 group, where 15 animals were analyzed (SOSIP.v5.1: five animals and SOSIP.v5.2: ten animals), the number of heterologous hits was divided by three. Statistical tests were performed using Mann-Whitney t test for (E)–(H) and Kruskal-Wallis with Dunn’s post-test for (I) and (P). Note that Kruskal-Wallis tests were performed on the ID_50_ values. The horizontal bars represent medians. Neutralization titers with SOSIP.v6 sera against 92RW020, SHIVP3, and TV1.21 (plotted in F–H and M–O) were obtained from two or three independent experiments performed in duplicate. The average values are plotted, and the data from the replicate experiments can be found in Table S4C.

We compared the immunogenicity of various prototypic and stabilized BG505 SOSIP trimers in rabbits using a previously described protocol with 22 μg of trimer per dose and measuring the antibody responses 2 weeks after the third immunization (Figure 2A) (Sanders et al., 2015, de Taeye et al., 2015).

All the BG505 trimer-immunized animals had high and comparable titers of binding antibodies to the corresponding trimers, as measured in ELISA (Figure 2C). The sera from the SOSIP.664 trimer recipients consistently neutralized the autologous tier-2 BG505.T332N virus, which is consistent with our earlier reports (Figure 2D) (Sanders et al., 2015, de Taeye et al., 2015). The corresponding gp120 monomer was almost entirely ineffective in this regard, with only one of five animals responding weakly (Figure 2D). The inferiority of BG505 gp120 at inducing autologous BG505.T332N NAbs was consistent with a previous report, although the difference between the gp120 recipients and the trimer-immunized animals was much more pronounced in the current experiment (Sanders et al., 2015; Figure 2D). The autologous NAb responses elicited by the different trimers were generally comparable (median half maximal inhibitory concentration [IC_50_]; SOSIP.664, 4432; SOSIP.v4.1, 4503; SOSIP.v5.1 + SOSIP.v5.2 pooled, 3457; and SOSIP.v6, 7,798), except for the SOSIP.v5.2 I201C-A433C hyperstabilized trimer (median IC_50_, 226; p = 0.0159 compared to SOSIP.664) (Figure 2D). Thus, with the possible exception of the I201C-A433C change to the SOSIP.v5.2 construct, the modifications used to create new, more stable trimers do not impair the induction of the autologous BG505.T332N NAb response.

One of the goals of trimer stabilization projects is to reduce the antigenicity and immunogenicity of epitopes for non-NAbs and tier-1 NAbs, the latter being dominated by V3-directed antibodies, and thereby focus the immune response on more productive targets (Kwon et al., 2015, de Taeye et al., 2015). Compared to the BG505 SOSIP.664 trimers and, more so, the gp120 monomers, NAb titers against the tier-1A SF162 virus were reduced by 3-fold for the combined SOSIP.v5.1 and SOSIP.v5.2 groups (p = 0.0175 versus SOSIP.664; not statistically significant versus SOSIP.v4.1) and were also lower for SOSIP.v5.2 201C-433C (not statistically significant versus SOSIP.664). In contrast, the SF162 NAb titers, as well as V3 binding antibody titers (data not shown), were almost 7-fold higher in the SOSIP.v6 group compared to the combined SOSIP.664, SOSIP.v4.1, and SOSIP.v5 groups (p = 0.0031; Figure 2E).

Sera from the various BG505 trimer-immunized rabbits were generally weakly and sporadically active against a panel of heterologous tier-2 viruses (Table S4A). While the clade A virus 92RW020, the clade B virus SHIV162P3, and the clade C TV1.21 virus were poorly or not neutralized by SOSIP.664-, SOSIP.v4.1-, or SOSIP.v5-immunized animals (median IC_50_ values for the combined SOSIP.664, SOSIP.v4.1, and SOSIP.v5 groups of 25, 20, and 28, respectively), they were more consistently neutralized by sera from SOSIP.v6 recipients (IC_50_ values of 56, 45, and 97). The differences in the ability to neutralize 92RW020, SHIV162P3, and TV1.21 between the BG505 SOSIP.v6 recipient animals and the animals that were immunized with earlier BG505 SOSIP versions were statistically significant (p = 0.0071, p = 0.0021, and p = 0.0207 for the three viruses, respectively). In contrast, prebleed sera did not neutralize the autologous BG505.T332N pseudovirus, nor the heterologous 92RW020 or SHIV162P3 pseudoviruses (Table S4C). For each individual rabbit, we analyzed the number of heterologous tier-2 viruses that were neutralized with IC_50_ values >40. Neutralization at this titer level was only sporadic for the SOSIP.664, SOSIP.v4.1, or SOSIP.v5 groups; the majority of the sera were inactive against all heterologous Tier-2 viruses. In contrast, sera from the SOSIP.v6 group neutralized one (one animal), two (one animal), three (one animal), or four (two animals) of the sixteen viruses in the test panel at a titer >40 (Figure 2I and Table S4A). When the SOSIP.v6 group was compared with the SOSIP.664, SOSIP.v4.1, SOSIP.v5.1 + SOSIP.v5.2, and SOSIP.v5.2 I201C-A433C groups, the number of heterologous Tier-2 viruses neutralized with IC_50_ values >40 was significantly higher (n = 14 for SOSIP.v6 versus n = 0, 4, 4, and 6 for SOSIP.664, SOSIP.v4.1, SOSIP.v5.1 + SOSIP.v5.2, and SOSIP.v5.2 I201C-A433C, respectively; Kruskal-Wallis p < 0.001 in each case) (Figure 2I).

### Immunogenicity of Stabilized ZM197M SOSIP Trimers in Rabbits

In a similar study, we tested the ZM197M SOSIP.664, SOSIP.v4.2, and SOSIP.v5.2 trimers in rabbits (Figures 2J–2O). The autologous trimer binding antibody titers were again comparable among the groups, but ∼2- to 5-fold lower than those induced by BG505 trimers (Figure 2J). The autologous NAb response to the ZM197M trimers, in general, was also markedly weaker and less consistent than seen with their BG505 counterparts (cf. Figure 2J with Figure 2C and Figure 2K with Figure 2D). The ZM197M SOSIP.v5.2 trimer was clearly the most immunogenic for the autologous NAb response (5/5 responders, with a median IC_50_ of 114, compared to 3/10 responders for the combined SOSIP.664 and SOSIP.v4.2 groups; p < 0.0001 by χ^2^ test; Figure 2K). The three ZM197M trimers induced SF162 tier-1A NAb titers to comparable extents (Figure 2L).

As seen in the BG505 study, sera against the various ZM197M trimers only sporadically neutralized heterologous tier-2 viruses (Table S4A). Overall, the most frequent NAb responses to 92RW020, SHIV162P3, and TV1.21 were induced by the ZM197M SOSIP.v5.2 variant (p = 0.007, non-significant and p = 0.0137 for 92RW020, SHIV162P3, and TV1.21, respectively compared to SOSIP.664 and SOSIP.v4.2 combined; Figures 2N–2P). Three of the five SOSIP.v5.2 sera neutralized three heterologous tier-2 viruses at a titer >40, while none of the SOSIP.664 or SOSIP.v4.2 sera did so (Kruskal-Wallis p < 0.001; Figure 2P). Assessed across the entire heterologous tier-2 panel, the ZM197M SOSIP.v5.2 sera neutralized significantly more viruses (IC_50_ values > 40) compared to the SOSIP.664 or SOSIP.v4.2 groups (n = 9 versus 0 or 0, respectively, Fischer-Freeman p < 0.0001; Figure 2P).

In conclusion, the hyperstable BG505 SOSIP.v6 trimers induced significantly broader tier-2 NAb responses than the SOSIP.664, SOSIP.v4, and SOSIP.v5 variants. For the ZM197M genotype, the SOSIP.v5.2 construct was the most broadly immunogenic for tier-2 NAbs compared to its SOSIP.664 and SOSIP.v4.2 counterparts.

### Properties of the Autologous Tier-2 NAb Specificities

To investigate what epitopes were targeted by the autologous BG505.T332N NAbs induced by, in particular, BG505 SOSIP.v6 trimers in rabbits, we first performed neutralization depletion experiments using a BG505 V3 peptide and a set of BG505 proteins: SOSIP.664 gp140, gp120, gp120-ΔV1V2, gp120-ΔV3, and gp120-7C3 (Sanders et al., 2015). All proteins contained the D368R substitution to prevent neutralization by CD4 binding and interference in neutralization assays. The gp120-7C3 contains seven substitutions in residues 354–363 in the C3 domain that were under selective pressure in the BG505-infected infant (Goo et al., 2014, Sanders et al., 2015). One BG505 SOSIP.v5.2 recipient animal was also included in the analyses.

**Figure 3:**
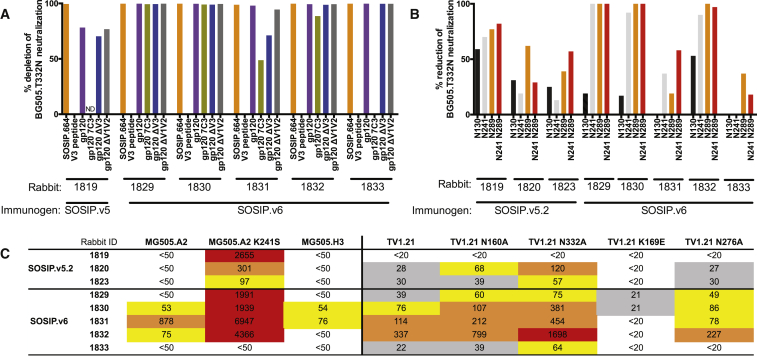
Properties of the Autologous NAb Specificities (A) Neutralization depletion assays with sera from one BG505 SOSIP.v5.2 recipient and all five BG505 SOSIP.v6-immunized animals using the autologous BG505.T332N virus and a BG505 V3 peptide, as well as a set of different BG505 proteins. Neutralization of BG505.T332N from each individual sera was set at 100%, and the percent depletion was calculated when the V3 peptide or the different BG505 proteins were added in the assay. The percentage shown is the mean of two different experiments. (B) Neutralization assays with BG505.T332N PNGS mutants. The ID_50_ of the individual sera with the parental BG505.T332N virus was set at 100%, and the percent reduction in ID_50_ when the respective PNGSs are introduced is shown. See also Table S4B. (C) Neutralization of maternal MG505 viruses and heterologous TV1.21 mutant viruses. The ID_50_s are shown. Strong neutralization (ID_50_ > 1,000) is shown in red; 1,000 > ID_50_ > 100 is shown in orange; 100 > ID_50_ > 50 is shown in yellow, and no neutralization (ID_50_ < 50) is shown in white.

The autologous NAb response in all SOSIP.v6 recipients and the one SOSIP.v5.2 recipient rabbit was completely depleted when the sera were preincubated with BG505 SOSIP.664 D368R trimer, confirming that the neutralization was trimer directed (Figure 3A). gp120, gp120-ΔV1V2, and gp120-ΔV3 were also efficient at depleting the BG505.T332N neutralization, indicating that most of the autologous NAb response was directed to gp120-associated epitope(s) that did not involve V1V2 or V3. Rabbit 1831 was an exception. In this animal, gp120-ΔV3 was considerably less efficient at depleting BG505.T332N neutralization compared to full-length gp120, suggesting that the V3 domain was involved in the (presentation of) the epitope for the autologous NAb response in this animal. The gp120-7C3 protein was unable to deplete the BG505.T332N NAb activity from the sera of rabbit 1833 and poorly effective in depleting the BG505.T332N NAb activity from animal 1831, suggesting that residues 354–363, in the C3 domain, are involved in the epitope(s) for the autologous NAb activity in these animals. BG505.T332N neutralization of all sera was unaffected by addition of the BG505 V3 peptide, confirming that autologous NAb response was not directed against simple linear epitopes in the V3 domain that are associated with tier-1A virus neutralization (Figure 3A; Sanders et al., 2015).

Next, we tested neutralization of BG505.T332N mutants, specifically focusing on virus mutants in which holes in the BG505 trimer glycan shield that are immunogenic in rabbits were filled in by restoring the respective predicted N-linked glycan sites (PNGSs) (Klasse et al., 2016, McCoy et al., 2016). We previously found that these holes, especially a large glycan hole centered around positions 241 and 289, are frequent targets of autologous BG505.T332N NAb responses induced by BG505 SOSIP trimers (Klasse et al., 2016).

Introduction of PNGSs at positions 241 and 289 almost completely abolished BG505.T332N neutralization by sera from three out of five SOSIP.v6 recipients, as well as one SOSIP.v5.2 recipient (animals 1819, 1829, 1830, and 1832), showing that the NAb activity in these animals was dominated by Ab specificities targeting the 241/289 hole (Figure 3B; Table S4B). The NAb activity in four other rabbits tested, two receiving SOSIP.v5.2 and two receiving SOSIP.v6 (animals 1831 and 1833), were much less affected by the PNGSs knockin mutations, showing that the NAb response in those rabbits was only partly directed to the 241/289 glycan hole. The introduction of the PNGSs at position 130 generally had a much smaller effect on the NAb sensitivity compared to the introduction of PNGSs at positions 241 and 289. The largest effects were observed for animals 1819 (SOSIP.v5.2) and 1832 (SOSIP.v6), in which cases the NAb activity was reduced by ∼50%.

The finding that introduction of the PNGSs at 241 and 289 only had a partial effect on the NAb activity in animals 1831 and 1833 is consistent with the observation that the gp120-7C3 protein did not deplete the NAb activity from the sera of these two animals. Thus, while the NAb specificities of three SOSIP.v6 recipient animals appear to be directed to the 241/289 glycan hole, those in the two other animals appear to be predominantly targeting an epitope that is affected by residues 354–363.

We also tested the maternal virus clones MG505.A2 and MG505.H3. While MG505.A2 has 14 amino-acid differences in gp160 compared to BG505.T332N, only one involves a change in a PNGSs: N332T. MG505.H3 contains 24 amino-acid differences compared to BG505.T332N, including the presence of a PNGS at 241 and the lack of PNGSs at positions 190, 234, 332, 363, and 411.

The sera of three rabbits immunized with BG505 SOSIP.v6 neutralized MG505.A2, but none immunized with BG505 SOSIP.v5.2 did. While MG505.A2 has a hole at position 241, the amino acid at that position is different compared to BG505.T332N (K for MG505.A2 and S for BG505.T332N). To mimic the BG505.T332N virus, a K241S substitution was introduced into MG505.A2. The sera from all the rabbits, except one animal, became substantially more sensitive to this virus, suggesting that the NAb specificities in these sera were directed toward this glycan hole, but were also dependent on the composition of amino acid 241. One exception was animal 1833, which did not neutralize MG505.A2 or its K241S variant, in agreement with the observation that the response in this animal did not target the 241/289 hole. Furthermore, although the serum from animal 1831 potently neutralized the MG505.A2 K241S virus, it also neutralized the parental MG505.A2 virus effectively, providing further evidence that NAb specificities other than those directed to the 241/289 hole were present in this animal. Finally, when sera were tested against the MG505.H3 virus, only two rabbits (1830 and 1831, both recipients of BG505 SOSIP.v6) neutralized this virus and then at low titer (Figure 3C), suggesting that any or all of the 24 amino-acid changes in the MG505.H3 virus cause resistance to the NAb specificities present in the SOSIP.v6-immunized rabbit sera.

Finally, to obtain information on the heterologous NAb responses, we tested some of the sera against a set of TV1.21 pseudovirus mutants. The results showed that knocking out the N160 or N332 glycan enhanced TV1.21 neutralization by SOSIP.v6 sera, suggesting that these glycans shield the heterologous neutralization epitope(s) targeted by the SOSIP.v6 recipient animals. Conversely, the K169E mutation ablated neutralization, suggesting that K169 is part of the neutralization epitope, or that the K169E mutation destroys the target epitope by indirect means. Removal of the N276 glycan had no effect on the sensitivity to the SOSIP.v6 sera (Figure 3C; Table S4C). Because of the poor magnitude of the other heterologous NAb titers, we were not able to map them.

## Discussion

We describe the creation of native-like SOSIP trimers that are stabilized by the introduction of additional disulfide bonds. The most stable variant, BG505 SOSIP.v6, has its major *T*_m_ at 92.2°C and a minor *T*_m_ at 78.8°C, representing an increase of 24.5°C and 11.1°C over the prototypic SOSIP.664 design. The most stable trimers contain two new disulfide bonds per gp120-gp41 protomer, in addition to SOS. The 30 intra-molecular disulfide bonds naturally present in the trimer (ten per protomer) are located within the inner or outer domains of gp120 or, in one case, the immunodominant loop of gp41. In contrast, the engineered bonds are all inter-domain.

The inter-subunit disulfide bond linking gp120 residue-501 to gp41 residue-605 (the “SOS bond”) was positioned without the availability of structural information on the gp120-gp41 interface (Binley et al., 2000). The cryo-EM structure of the native, membrane-associated trimer (Lee et al., 2016) confirms the accuracy with which this bond was placed in the SOSIP trimer. The newly introduced gp41 cysteine residues that successfully form inter-subunit disulfide bonds are located between the α6 and α7 segments (i.e., HR1_N_) that were not well resolved in the initial BG505 SOSIP.664 trimer structures (Julien et al., 2013a, Lyumkis et al., 2013, Pancera et al., 2014). More recent structures and HD-X experiments imply that this region in the SOSIP structures might be quite dynamic and, hence, capable of adopting multiple conformations (Garces et al., 2015, Guttman et al., 2014). The finding that cysteine residues at multiple positions in gp41 HR1 can pair with gp120 residues 72 or 73 is consistent with the flexibility of this region (Lee et al., 2016). However, the trimer structure predicted various other positions for cysteine substitutions that did not, in practice, lead to the efficient formation of new disulfide bonds. One explanation is that the presence of additional cysteine residues can sometimes interfere with the proper oxidative folding and disulfide bond isomerization in the endoplasmic reticulum, leading to the production of misfolded proteins that are subsequently degraded (Bulleid and van Lith, 2014).

While a previous report showed a correlation between trimer stability and the ability to induce autologous NAb responses (Feng et al., 2016), we did not observe such a correlation here. Thus, the BG505 SOSIP.664, SOSIP.v4.1, SOSIP.v5.1, SOSIP.v5.2, and SOSIP.v6 trimers, displaying a wide range of stabilities, were all equivalent in inducing BG505.T332N NAbs. However, the observation that the BG505 SOSIP.v6 trimer and the ZM197M SOSIP.v5 trimer were more efficient at inducing heterologous tier-2 NAb responses suggests that the relationship between trimer stability and the ability to induce NAbs is likely to be complex. Regardless, the BG505 SOSIP.v6 and ZM197M SOSIP.v5.2 trimers have encouraging immunogenicity properties in rabbits and constitute a baseline for further trimer design and delivery improvements. An additional virtue of a more stable vaccine immunogen is an increased shelf life and simplified storage capacity under real-word conditions (Karp et al., 2015).

From a broader perspective, the reduced conformational flexibility of stabilized trimers may help maintain them in the ground state for longer in vivo, thereby maximizing the presentation of bNAb epitopes and increasing the probability of a successful encounter with susceptible but rare B cells. How these various factors intersect to drive the induction of higher titer autologous and heterologous tier-2 NAb titers is likely to be also influenced by the genotype of the trimer, as well as its design.

## Experimental Procedures

### Trimer Expression and Purification

The constructs expressing BG505, B41, AMC008, and ZM1097M SOSIP.664 were transiently expressed in adherent 293T cells or suspension 293F cells in the presence of *furin* and purified using PGT145-affinity chromatography, as previously described (Julien et al., 2013a, Julien et al., 2015, Pugach et al., 2015, Sanders et al., 2013, de Taeye et al., 2015).

### Antigenicity Assays

SPR analyses were performed with His-tagged trimers immobilized to CM5 chips, as previously described (Derking et al., 2015, Yasmeen et al., 2014). D7324-capture, nickel nitrilotriacetic acid (Ni-NTA)-capture, and thermostability ELISAs have been described elsewhere (Derking et al., 2015, Sanders et al., 2013, de Taeye et al., 2015).

### Biophysical Techniques

Multiple biophysical techniques were used to analyze the properties of the Env trimers, including DLS, SAXS, differential scanning calorimetry (DSC), hydrophilic interaction chromatography-ultra-performance liquid chromatography (HILIC-UPLC), and HDX-MS. Images of Env trimers were generated by negative stain EM (NS-EM) following previously described procedures (Sanders et al., 2013). X-ray crystallography was performed with BG505 Env trimer together with different Fabs as previously described (Julien et al., 2013a).

### Neutralization Assays

We used Env-pseudotyped or chimeric molecular clone viruses to perform neutralization assays at Duke University Medical Center, Academic Medical Center, and Weill Medical College of Cornell University (Sanders et al., 2013, Klasse et al., 2016).

### Statistics

Different groups in rabbit immunizations were compared by two-tailed Mann-Whitney U tests. Kruskal-Wallis with Dunn’s post-test was used when differences between the entire heterologous tier-2 panel were addressed.

### Rabbit Immunizations

Rabbits were immunized with 22 μg of trimer and ISCOMATRIX at weeks 0, 4, and 20. Rabbit immunizations were performed under contract at Covance Research Products Inc. (Denver, PA, USA) under permits with approval numbers C0022-15, C0119-15, and C0120-15.

## Author Contributions

Conceptualization, A.T.d.l.P., J.-P.J., R.W.S., and I.A.W.; Methodology, A.T.d.l.P., J.-P.J., S.W.d.T., M.G., G.O., L.K.P., A.-J.B., E.P.G., J.A.B., E.E.S., K.S., T.J.K., P.P., A.Y., C.A.C., J.L.T., M.J.v.G., C.L., D.C.M., H.D., M.C., P.J.K., K.K.L., J.P.M., A.B.W., I.A.W., and R.W.S.; Writing - Original Draft, A.T.d.l.P., J.-P.J., J.P.M., A.B.W., I.A.W., and R.W.S.; Writing - Review & Editing, A.T.d.l.P., J.-P.J., S.W.d.T., F.G., M.G., G.O., T.J.K., P.P., C.D.V., D.C.M., H.D., M.C., P.J.K., K.K.L., J.P.M., A.B.W., I.A.W., and R.W.S.; and Funding Acquisition, A.B.W., J.P.M., I.A.W., and R.W.S.

## Accession Numbers

The accession number for the trimer sequences reported in this paper is PDB: 5WDU.

## Supplemental Information

Document S1. Supplemental Experimental Procedures, Figures S1–S5, and Tables S1–S3 and S5

Table S4. Midpoint Neutralization Titers of Sera from Rabbits Immunized with Stabilized SOSIP Trimers, Related to Figures 2 and 3 The table shows midpoint neutralization titers of rabbit sera tested against the parental BG505.T332N (wild-type), and pseudoviruses in which specific PNGSs were mutated, and midpoint neutralization titers of rabbit sera from prebleeds and week 22 sera against autologous and heterologous pseudoviruses.

Data S1. Supportive Data for HDX and ELISA Experiments, Related to Figure 1 and Table 2 (A) Individual exchange plots for peptides derived from trimers. The percentage exchange at each time point is shown. The error bars show SDs derived from duplicate measurements. (B–E) Antigenicity of BG505, AMC008, B41, and ZM197M trimers was determined by ELISA. Binding curves, representative of two or three experiments, are shown.

Document S2. Article plus Supplemental Information

## References

[bib1] Binley J.M., Sanders R.W., Clas B., Schuelke N., Master A., Guo Y., Kajumo F., Anselma D.J., Maddon P.J., Olson W.C. (2000). A recombinant human immunodeficiency virus type 1 envelope glycoprotein complex stabilized by an intermolecular disulfide bond between the gp120 and gp41 subunits is an antigenic mimic of the trimeric virion-associated structure. J. Virol..

[bib2] Bulleid N.J., van Lith M. (2014). Redox regulation in the endoplasmic reticulum. Biochem. Soc. Trans..

[bib3] Camacho C.J., Thirumalai D. (1995). Modeling the role of disulfide bonds in protein-folding: entropic barriers and pathways. Proteins.

[bib4] Cheng C., Pancera M., Bossert A., Schmidt S.D., Chen R., Chen X., Druz A., Narpala S., Doria-Rose N.A., McDermott A.B. (2015). Immunogenicity of a prefusion HIV-1-envelope trimer in complex with a quaternary-specific antibody. J. Virol..

[bib5] Creighton T.E. (1988). Disulphide bonds and protein stability. BioEssays.

[bib6] de Taeye S.W., Ozorowski G., Torrents de la Peña A., Guttman M., Julien J.P., van den Kerkhof T.L.G.M., Burger J.A., Pritchard L.K., Pugach P., Yasmeen A. (2015). Immunogenicity of stabilized HIV-1 Envelope trimers with reduced exposure of non-neutralizing epitopes. Cell.

[bib7] Derking R., Ozorowski G., Sliepen K., Yasmeen A., Cupo A., Torres J.L., Julien J.P., Lee J.H., van Montfort T., de Taeye S.W. (2015). Comprehensive antigenic map of a cleaved soluble HIV-1 envelope trimer. PLoS Pathog..

[bib8] Feng Y., Tran K., Bale S., Kumar S., Guenaga J., Wilson R., de Val N., Arendt H., DeStefano J., Ward A.B. (2016). Thermostability of well-ordered HIV spikes correlates with the elicitation of autologous tier 2 neutralizing antibodies. PLoS Pathog..

[bib9] Garces F., Lee J.H., de Val N., Torrents de la Peña A., Kong L., Puchades C., Hua Y., Stanfield R.L., Burton D.R., Moore J.P. (2015). Affinity maturation of a potent family of HIV antibodies is primarily focused on accommodating or avoiding glycans. Immunity.

[bib10] Goo L., Chohan V., Nduati R., Overbaugh J. (2014). Early development of broadly neutralizing antibodies in HIV-1-infected infants. Nat. Med..

[bib11] Guenaga J., de Val N., Tran K., Feng Y., Satchwell K., Ward A.B., Wyatt R.T. (2015). Well-ordered trimeric HIV-1 subtype B and C soluble spike mimetics generated by negative selection display native-like properties. PLoS Pathog..

[bib12] Guttman M., Garcia N.K., Cupo A., Matsui T., Julien J.P., Sanders R.W., Wilson I.A., Moore J.P., Lee K.K. (2014). CD4-induced activation in a soluble HIV-1 Env trimer. Structure.

[bib13] Huang J., Kang B.H., Pancera M., Lee J.H., Tong T., Feng Y., Georgiev I.S., Chuang G.Y., Druz A., Doria-Rose N.A. (2014). Broad and potent HIV-1 neutralization by a human antibody that binds the gp41-120 interface. Nature.

[bib14] Julien J.P., Cupo A., Sok D., Stanfield R.L., Lyumkis D., Deller M.C., Klasse P.-J., Burton D.R., Sanders R.W., Moore J.P. (2013). Crystal structure of a soluble cleaved HIV-1 envelope trimer. Science.

[bib15] Julien J.P., Sok D., Khayat R., Lee J.H., Doores K.J., Walker L.M., Ramos A., Diwanji D.C., Pejchal R., Cupo A. (2013). Broadly neutralizing antibody PGT121 allosterically modulates CD4 binding via recognition of the HIV-1 gp120 V3 base and multiple surrounding glycans. PLoS Pathog..

[bib16] Julien J.P., Lee J.H., Ozorowski G., Hua Y., Torrents de la Peña A., de Taeye S.W., Nieusma T., Cupo A., Yasmeen A., Golabek M. (2015). Design and structure of two HIV-1 clade C SOSIP.664 trimers that increase the arsenal of native-like Env immunogens. Proc. Natl. Acad. Sci. USA.

[bib17] Karp C.L., Lans D., Esparza J., Edson E.B., Owen K.E., Wilson C.B., Heaton P.M., Levine O.S., Rao R. (2015). Evaluating the value proposition for improving vaccine thermostability to increase vaccine impact in low and middle-income countries. Vaccine.

[bib18] Klasse P.J., Labranche C.C., Ketas T.J., Ozorowski G., Cupo A., Pugach P., Ringe R.P., Golabek M., van Gils M.J., Guttman M. (2016). Sequential and simultaneous immunization of rabbits with HIV-1 envelope glycoprotein SOSIP.664 trimers from clades A, B and C. PLoS Pathog..

[bib19] Kong L., He L., de Val N., Vora N., Morris C.D., Azadnia P., Sok D., Zhou B., Burton D.R., Ward A.B. (2016). Uncleaved prefusion-optimized gp140 trimers derived from analysis of HIV-1 envelope metastability. Nat. Commun..

[bib20] Kwon Y.D., Pancera M., Acharya P., Georgiev I.S., Crooks E.T., Gorman J., Joyce M.G., Guttman M., Ma X., Narpala S. (2015). Crystal structure, conformational fixation and entry-related interactions of mature ligand-free HIV-1 Env. Nat. Struct. Mol. Biol..

[bib21] Lee J.H., Ozorowski G., Ward A.B. (2016). Cryo-EM structure of a native, fully glycosylated, cleaved HIV-1 envelope trimer. Science.

[bib22] Liszka M.J., Clark M.E., Schneider E., Clark D.S. (2012). Nature versus nurture: Developing enzymes that function under extreme conditions. Annu. Rev. Chem. Biomol. Eng..

[bib23] Lyumkis D., Julien J.P., de Val N., Cupo A., Potter C.S., Klasse P.-J., Burton D.R., Sanders R.W., Moore J.P., Carragher B. (2013). Cryo-EM structure of a fully glycosylated soluble cleaved HIV-1 envelope trimer. Science.

[bib24] McCoy L.E., van Gils M.J., Ozorowski G., Messmer T., Briney B., Voss J.E., Kulp D.W., Macauley M.S., Sok D., Pauthner M. (2016). Holes in the glycan shield of the native HIV envelope are a target of trimer-elicited neutralizing antibodies. Cell Rep..

[bib25] McLellan J.S., Chen M., Leung S., Graepel K.W., Du X., Yang Y., Zhou T., Baxa U., Yasuda E., Beaumont T. (2013). Structure of RSV fusion glycoprotein trimer bound to a prefusion-specific neutralizing antibody. Science.

[bib26] Pancera M., Zhou T., Druz A., Georgiev I.S., Soto C., Gorman J., Huang J., Acharya P., Chuang G.-Y., Ofek G. (2014). Structure and immune recognition of trimeric pre-fusion HIV-1 Env. Nature.

[bib27] Pritchard L.K., Vasiljevic S., Ozorowski G., Seabright G.E., Cupo A., Ringe R., Kim H.J., Sanders R.W., Doores K.J., Burton D.R. (2015). Structural constraints determine the glycosylation of HIV-1 envelope trimers. Cell Rep..

[bib28] Pritchard L.K., Harvey D.J., Bonomelli C., Crispin M., Doores K.J. (2015). Cell- and protein-directed glycosylation of native cleaved HIV-1 envelope. J. Virol..

[bib29] Pugach P., Ozorowski G., Cupo A., Ringe R., Yasmeen A., de Val N., Derking R., Kim H.J., Korzun J., Golabek M. (2015). A native-like SOSIP.664 trimer based on an HIV-1 subtype B *env* gene. J. Virol..

[bib30] Sanders R.W., Moore J.P. (2014). HIV: A stamp on the envelope. Nature.

[bib31] Sanders R.W., Vesanen M., Schuelke N., Schiffner L., Kalyanaraman R., Berkhout B., Maddon P.J., Olson W.C., Lu M., Moore J.P. (2002). Stabilization of the soluble, cleaved, trimeric form of the envelope glycoprotein complex of human immunodeficiency virus type 1. J. Virol..

[bib32] Sanders R.W., Derking R., Cupo A., Julien J.P., Yasmeen A., de Val N., Kim H.J., Blattner C., de la Peña A.T., Korzun J. (2013). A next-generation cleaved, soluble HIV-1 Env trimer, BG505 SOSIP.664 gp140, expresses multiple epitopes for broadly neutralizing but not non-neutralizing antibodies. PLoS Pathog..

[bib33] Sanders R.W., van Gils M.J., Derking R., Sok D., Ketas T.J., Burger J.A., Ozorowski G., Cupo A., Simonich C., Goo L. (2015). HIV-1 neutralizing antibodies induced by native-like envelope trimers. Science.

[bib34] Scharf L., Wang H., Gao H., Chen S., McDowall A.W., Bjorkman P.J. (2015). Broadly neutralizing antibody 8ANC195 recognizes closed and open states of HIV-1 Env. Cell.

[bib35] Steichen J.M., Kulp D.W., Tokatlian T., Escolano A., Dosenovic P., Stanfield R.L., McCoy L.E., Ozorowski G., Hu X., Kalyuzhniy O. (2016). HIV vaccine design to target germline precursors of glycan-dependent broadly neutralizing antibodies. Immunity.

[bib36] Stewart-Jones G.B., Soto C., Lemmin T., Chuang G.Y., Druz A., Kong R., Thomas P.V., Wagh K., Zhou T., Behrens A.J. (2016). Trimeric HIV-1-Env structures define glycan shields from clades A, B, and G. Cell.

[bib37] van Gils M.J., Sanders R.W. (2013). Broadly neutralizing antibodies against HIV-1: templates for a vaccine. Virology.

[bib38] Yasmeen A., Ringe R., Derking R., Cupo A., Julien J.P., Burton D.R., Ward A.B., Wilson I.A., Sanders R.W., Moore J.P., Klasse J.P. (2014). Differential binding of neutralizing and non-neutralizing antibodies to native-like soluble HIV-1 Env trimers, uncleaved Env proteins, and monomeric subunits. Retrovirology.

