# Supplementary material for: Improving the Immunogenicity of Native-like HIV-1 Envelope Trimers by Hyperstabilization
Source: Cell Rep. 2017 Aug 23;20(8):1805–17. doi: 10.1016/j.celrep.2017.07.077 (PMC5590011; doi:10.1016/j.celrep.2017.07.077)
Supplement: Document S1. Supplemental Experimental Procedures, Figures S1–S5, and Tables S1–S3 and S5 [file mmc1.pdf]

Cell Reports, Volume *20*

Supplemental Information

Improving the Immunogenicity of Native-like HIV-1 Envelope Trimers by Hyperstabilization

Alba Torrents de la Peña, Jean-Philippe Julien, Steven W. de Taeye, Fernando Garces, Miklos Guttman, Gabriel Ozorowski, Laura K. Pritchard, Anna-Janina Behrens, Eden P. Go, Judith A. Burger, Edith E. Schermer, Kwinten Sliepen, Thomas J. Ketas, Pavel Pugach, Anila Yasmeen, Christopher A. Cottrell, Jonathan L. Torres, Charlotte D. Vavourakis, Marit J. van Gils, Celia LaBranche, David C. Montefiori, Heather Desaire, Max Crispin, Per Johan Klasse, Kelly K. Lee, John P. Moore, Andrew B. Ward, Ian A. Wilson, and Rogier W. Sanders

Figure S1

A

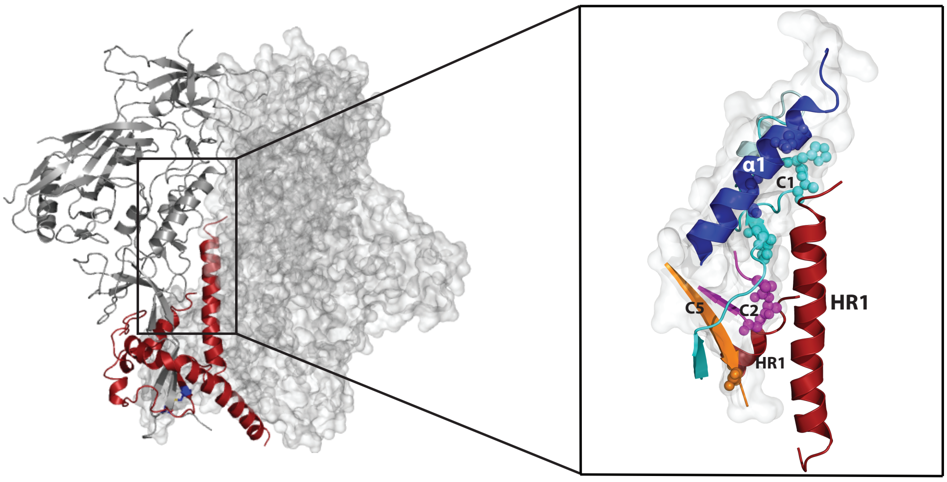

B

Location	Disulfide Bond	Trimerization	2G12	PGT145	PGT151	VRC01	PGT121	PG16	19b	F240	17b	17b+sCD4
C5 - DL	501-605	+++	+++	+++	+++	+++	+++	+++	+++	-	++	+++
C1-HR1	51-574	+	++	-	-							
	51-578	++	++	++	+							
	53-564	-	+	-	-							
	53-573	-	++	-	++							
	53-574	+++	++	+	+							
	72-554	++	++	++	+							
	72-555	+	++	++	++							
	72-564	++	+++	+++	+++	+++	+++	+++	+++	++	-	++
	73-558	++	+++	+++	+++	+++	+++	+++	+++	++	-	++
	73-561	++	+++	+++	+++	+++	+++	+++	+++	++	-	++
	73-567	++	+++	++	-							
	73-568	++	+++	+++	+++	+++	+++	+++	+++	++	-	++
α1-HR1	107-557	-	+	-	-							
	113-557	-	+	-	-							
	113-567	++	++	-	-							
loop A - HR1	220-568	+	+	+	++							
	221-568	+	++	+	+							
	221-571	-	+	+	-							
	221-578	+	++	-	-							
	221-579	+	++	+	++							
	221-582	+	++	++	++							
	222-571	-	+	-	-							
	222-579	-	++	-	-							
C5-HR1	492-574	-	++	-	-							
	492-585	++	++	+	++							

C

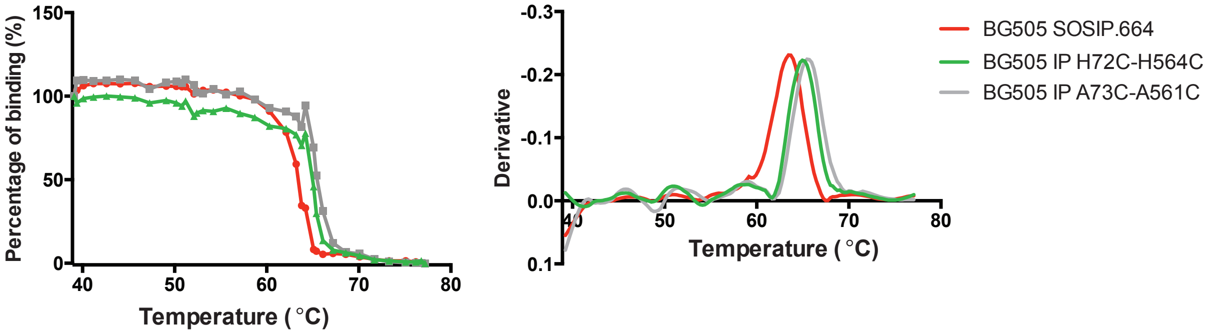

D

	T_m (°C)	ΔT_m (°C)
BG505 SOSIP.664	63.7	0.0
BG505 IP H72C-H564C	65.0	+1.3
BG505 IP A73C-A558C	64.5	+0.8
BG505 IP A73C-A561C	65.5	+2.0
BG505 IP A73C-A568C	64.0	+0.3
BG505 SOSIP.664 H72C-H564C	65.5	+1.8
BG505 SOSIP.664 A73C-A561C	66.0	+2.3

Figure S1. **Introducing a novel disulfide bond between gp120 and gp41.** Related to Fig. 1. (**A**) Five regions in gp120 and gp41 that are in reasonably close contact are shown in the crystal structure of BG505 SOSIP.664 (PDB: 4TVP). These regions involve residues in C1, C2 and C5 of gp120 and HR1 of gp41. The gp120 and gp41 subunits of a gp120-gp41 protomer are shown in grey and dark red, respectively. The SOS disulfide bond between residues 501 and 605 is colored blue. A close view of the gp120-gp41 interface is presented in the inset, where gp120 residues that likely contact gp41 are shown as blue spheres. (**B**) Screening of a panel of unpurified His-tagged BG505 SOSIP.664 proteins in which the original SOS disulfide bond (Cys501-Cys605) was replaced by two new cysteine residues in the regions highlighted in panel **A**, above. We used BG505 SOSIP.664 structures described in Julien et al., 2013b and Pancera et al., 2014 to design the structure-based disulfide bonds. BN-PAGE followed by western blotting was used to assess trimerization efficiency, which was reduced compared to SOSIP.664 for the majority of the mutants. The antigenic profile of the same trimer variants was determined by His-tag ELISA. If no disulfide bond is formed between the gp120 and gp41 subunits, they dissociate rapidly; the binding of the anti-gp120 MAb (2G12) to a construct captured via the His-tag on the gp41 subunit is therefore evidence for the successful formation of an intermolecular disulfide bond (Blattner et al., 2014; Ringe et al., 2013; Sanders et al., 2013). Several of the new constructs, particularly those with a cysteine residue introduced at position 72 or 73 in the loop between $\alpha\bar{1}$ and β0 together with one inserted within positions 554-568 in the loop between α6 and α7, were strongly reactive with 2G12. The implication is that an inter-subunit disulfide bond could form in these constructs. Two bNAbs that recognize different quaternary structure-dependent epitopes (PGT145: V1V2-apex; PGT151: gp120-gp41 interface) were used to assess the antigenic structure of the mutant trimers. Four mutants, H72C-H564C, A73C-A558C, A73C-A561C and A73C-L568C, bound to both PGT145 and PGT151 to similar extents to the original BG505 SOSIP.664 trimer; these cysteine pairs preserve the antigenic structure of the trimers appropriately. The antigenic profile of these four trimers was then assessed using a larger panel of bNAbs, which showed that the quaternary structure-dependent bNAbs 35022, 3BC315 and PG16 all bound efficiently each trimer. The spontaneous opening of the trimer variants was investigated using the CD4i non-NAb 17b, which did not bind to any of the four trimers in the absence of sCD4 in contrast with SOSIP.664, and did so only weakly when sCD4 was present. The anti-gp41 non-NAb F240 bound slightly more strongly to the four mutant trimers than to the original BG505 SOSIP.664 version, suggesting that the absence of the 501-605 disulfide exposes this epitope to some extent. This was confirmed using purified trimers (not shown). The F240 epitope in the immunodominant disulfide loop region of gp41 is known to overlap with residue 605, where the Cys residue involved in the SOS bond is located (Cavacini et al., 1998). Color code: red, no binding; orange, moderate binding; green, strong binding. Antibody binding is also scaled from no reactivity (-) to strong binding (+++). The data presented are based on 2 or 3 experiments. (**C**) The stability of unpurified His-tagged trimer mutants was evaluated using a thermal melting assay described elsewhere (de Taeye et al., 2015). The proteins were incubated for 1 h at varying temperatures in a graded PCR machine before 2G12 binding was assessed by Ni-NTA ELISA (right panel). The first derivative reveals the unfolding pattern (right panel). (**D**) The midpoints of thermal denaturation (T_m) for each trimer variant are based on the melting profiles in panel **C**. The T_m values for mutants containing the H72C-H564C or A73C-A561C disulfide bonds were increased by 1.3°C and 2.0°C, respectively, compared to SOSIP.664. The implication is that locating the disulfide bond at the core of the trimer, as opposed to the membrane proximal end in SOSIP.664, increases its stability. These results were conformed with purified trimers and DSC analysis (not shown). The T_m values for additional mutant trimers containing two disulfide proteins are also presented for reference (see Fig. S2).

Figure S2

A

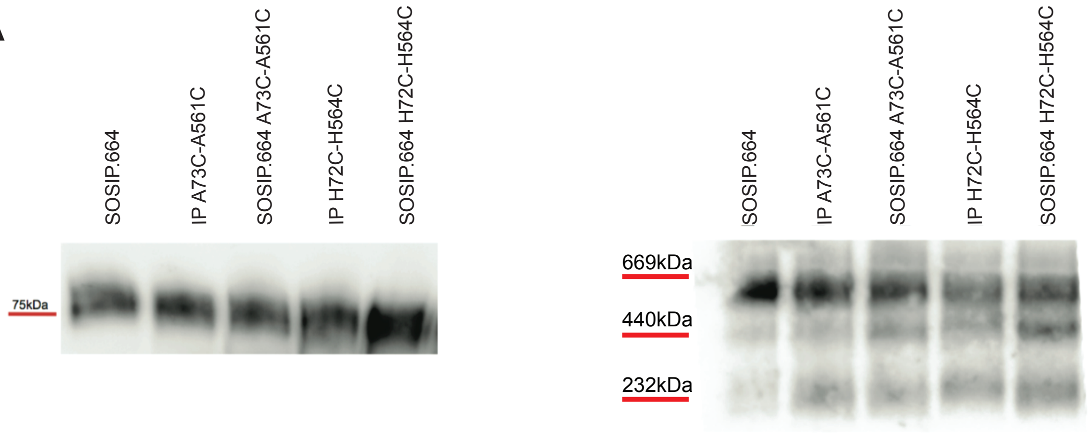

B

	2G12	PGT145	PGT151	VRC01	19b	17b	F240
BG505 SOSIP.664	+++	+++	+++	+++	++	++	-
BG505 IP H72C-H564C	+++	+++	+++	+++	++	-	++
BG505 IP A73C-A561C	+++	+++	+++	+++	++	-	++
BG505 SOSIP.664 H72C-H564C	+++	+++	+++	+++	++	-	-
BG505 SOSIP.664 A73C-A561C	+++	+++	+++	+++	++	-	-

C

BG505 SOSIP.664

Disulfide bond	2G12	PGT145	PGT151	17b	T_m (°C)
SOSIP.664	+++	+++	+++	+/-	64.0
H72C	++	+	+	-	63.3
A73C	++	-	+	-	58.2
H72C-A558C	++	++	++	-	ND
H72C-P559C	-	-	-	-	63.3
H72C-E560C	+++	++	+++	-	ND
H72C-A561C	+++	+++	+++	-	65.3
H72C-Q562C	++	-	+	-	ND
H72C-Q563C	+++	+++	+++	-	65.1
**H72C-H564C**	+++	+++	+++	-	65.6
H72C-L565C	+++	+++	+++	-	66.0
H72C-L566C	+++	+	+	-	63.8
H72C-K567C	+++	+++	+++	-	65.6
A73C-A558C	+++	++	+++	-	65.6
A73C-P559C	+	-	-	-	ND
A73C-E560C	+++	++	+++	-	65.3
**A73C-A561C**	+++	+++	+++	-	66.1
A73C-Q562C	+++	-	+	-	ND
A73C-Q563C	+++	+++	+++	-	64.2
A73C-H564C	+++	++	+++	-	65.3
A73C-L565C	+++	+++	+++	-	65.6
A73C-L566C	+++	++	+++	-	65.8
A73C-K567C	++	+	+	-	ND
A73C-L568C	+++	+++	+++	-	63.8
A73C-T569C	+++	+++	+++	-	65.1

Figure S2. Trimers with two disulfide bonds linking gp120 and gp41. Related to Fig. 1. (**A**) Unpurified His-tagged BG505 Env proteins that include the original SOS bond (Cys501-Cys605; i.e. SOSIP.664) or one of the new H72C-H564C or A73C-A561C bonds, instead of or in addition to the SOS bond, were assessed for cleavage and trimerization efficiency by reducing SDS-PAGE (left panel) and BN-PAGE (right panel). All of the new mutants were properly cleaved, but their trimerization was less efficient than for SOSIP.664. (**B**) The antigenic phenotype of the same unpurified variants was assessed by ELISA. The 2G12, VRC01, PGT145 and PGT151 bNAbs each bound strongly to all the variants, similar to SOSIP.664. The trimers containing only the H72C-H564C or the A73C-A561C disulfide bond (i.e., not the SOS bond) were less reactive with the CD4i non-NAb 17b, but more reactive gp41 non-NAb F240 (see Fig. S1). The mutants with two inter-subunit disulfide bonds had a phenotype reflecting the presence of each bond. Thus, 17b binding was not detected, a property shared with the H72C-H564C and A73C-A561C single disulfide bond mutants, while F240 binding was also very low, a property conferred by the SOS bond. (**C**) Additional Cys substitutions at most of the residues between 558 and 569 were screened for their ability to form disulfide bonds with Cys72 and Cys73, in mutants that also contained the SOS bond. The various unpurified mutant proteins were tested for 2G12, PGT145 and PGT151 reactivity and for thermostability, as described in Fig. S1. Several inter-subunit disulfide bonds could form with no apparent adverse influence on the overall conformation of the trimer, suggesting that the 558-569 region of gp41 is conformationally flexible. Thus, in general, the mutants bound quaternary structure-dependent bNAbs to a similar or higher extent than the original SOSIP.664 trimer, and their thermostability was higher, which is presumably attributable to the additional stabilizing effect of the second inter-subunit disulfide bond in the trimer core. Color code: red, no binding; orange, moderate binding; green, strong binding. Antibody binding is also scaled from no reactivity (-) to strong binding (+++). ND: Not determined. The data are based on 3 experiments.

Figure S3

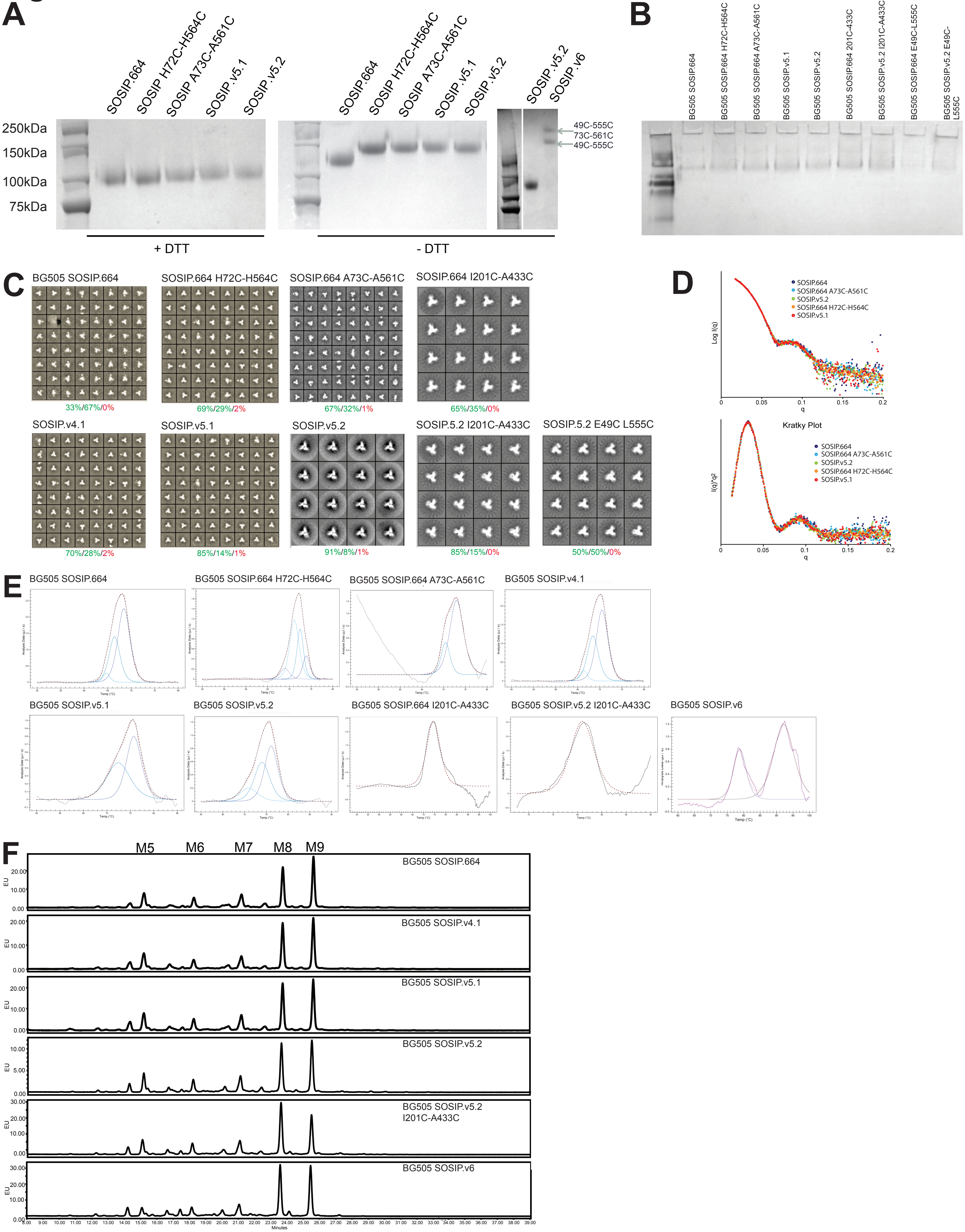
A
SOSIP.664
SOSIP H72C-H564C
SOSIP A73C-A561C
SOSIP.v5.1
SOSIP.v5.2
SOSIP.v6
250kDa
150kDa
100kDa
75kDa
49C-555C
73C-561C
49C-555C
+ DTT
- DTT
B
BG505 SOSIP.664
BG505 SOSIP.664 H72C-H564C
BG505 SOSIP.664 A73C-A561C
BG505 SOSIP.v5.1
BG505 SOSIP.v5.2
BG505 SOSIP.664 201C-433C
BG505 SOSIP.v5.2 I201C-A433C
BG505 SOSIP.664 E49C-L555C
BG505 SOSIP.v5.2 E49C-L555C
C
BG505 SOSIP.664
SOSIP.664 H72C-H564C
SOSIP.664 A73C-A561C
SOSIP.664 I201C-A433C
33%/67%/0%
69%/29%/2%
67%/32%/1%
65%/35%/0%
SOSIP.v4.1
SOSIP.v5.1
SOSIP.v5.2
SOSIP.5.2 I201C-A433C
SOSIP.5.2 E49C L555C
70%/28%/2%
85%/14%/1%
91%/8%/1%
85%/15%/0%
50%/50%/0%
D
Log I(q)
SOSIP.664
SOSIP.664 A73C-A561C
SOSIP.v5.2
SOSIP.664 H72C-H564C
SOSIP.v5.1
q
Kratky Plot
I(q)*q2
E
BG505 SOSIP.664
BG505 SOSIP.664 H72C-H564C
BG505 SOSIP.664 A73C-A561C
BG505 SOSIP.v4.1
BG505 SOSIP.v5.1
BG505 SOSIP.v5.2
BG505 SOSIP.664 I201C-A433C
BG505 SOSIP.v5.2 I201C-A433C
BG505 SOSIP.v6
F
M5
M6
M7
M8
M9
BG505 SOSIP.664
BG505 SOSIP.v4.1
BG505 SOSIP.v5.1
BG505 SOSIP.v5.2
BG505 SOSIP.v5.2 I201C-A433C
BG505 SOSIP.v6
EU
Minutes

Figure S3. Biochemical, biophysical and antigenic characterization of PGT145-purified stabilized BG505 SOSIP trimers. Related to Table 1 and Table 2 (**A**) Cleavage efficiency and disulfide bond formation was determined by SDS-PAGE under reducing and non-reducing conditions, respectively. Under reducing conditions, all proteins showed a gp120 band, indicating that they were completely cleaved. Under non-reducing conditions, the proteins all showed a gp140 band, but they migrated differently through the gels, indicative of different levels of compactness (compact proteins take up less SDS and therefore migrate slower through SDS-PAGE gels). The double disulfide bond variants all showed a slower migration pattern due to the presence of the extra bond. (**B**) The purified His-tagged BG505 SOSIP.664, SOSIP.v4.1 and SOSIP.v5 proteins were exclusively trimeric as determined by BN-PAGE. (**C**) The presence of native-like trimers in PGT145-purified preparations was assessed by NS-EM. The 2D reference-free class averages for the two double disulfide bond proteins (SOSIP.v5.1 and SOSIP.v5.2) are compared to SOSIP.664 and SOSIP.v4.1. The percentages of closed native-like and open native-like trimers (Pugach et al., 2015) are shown in green and the percentage of non-native trimers in red. (**D**) SAXS scattering curves (left panel) and Kratky plots (right panel) show the consistency of the scattering pattern among the various BG505 trimer mutants, indicating that they all have a similar overall structure in solution. (**E**) The thermal stabilities of BG505 SOSIP.664, SOSIP.v4.1 and SOSIP.v5 trimers were measured by DSC. The independent non-two state best-fit curves are depicted by a dashed red line and the T_m values of each peak are given in the graphs. See the Supplemental Experimental Procedures section for more details on curve modeling. (**F**) Glycan profiles of trimer variants were determined by HILIC-UPLC. $Man_{5-9}GlcNAc_2$ glycans are indicated as M5-M9 above the panel.

Figure S4

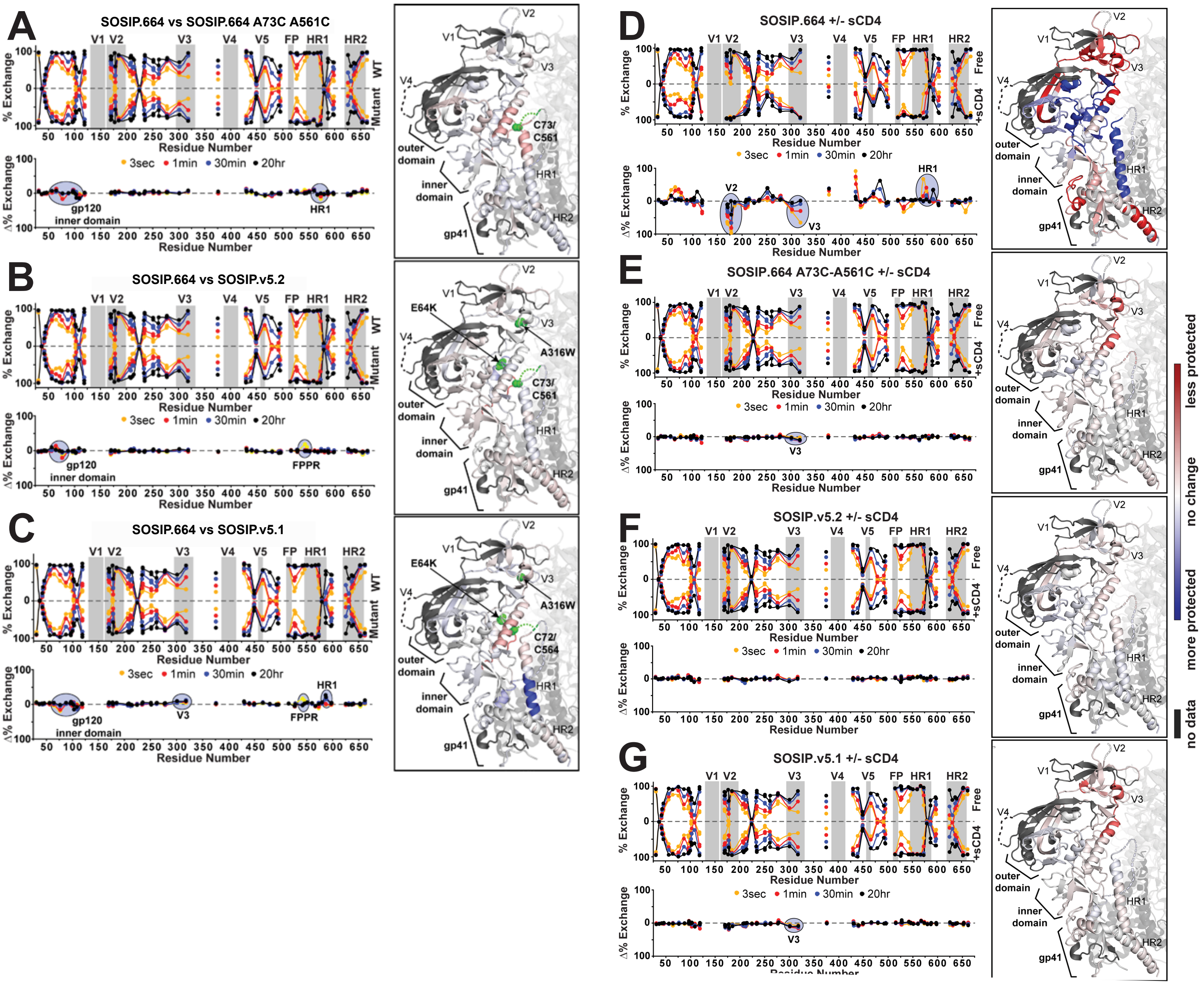

A
SOSIP.664 vs SOSIP.664 A73C A561C
B
SOSIP.664 vs SOSIP.v5.2
C
SOSIP.664 vs SOSIP.v5.1
D
SOSIP.664 +/- sCD4
E
SOSIP.664 A73C-A561C +/- sCD4
F
SOSIP.v5.2 +/- sCD4
G
SOSIP.v5.1 +/- sCD4
% Exchange
Δ% Exchange
Residue Number
3sec 1min 30min 20hr
WT
Mutant
Free
+sCD4
V1 V2 V3 V4 V5 FP HR1 HR2
FPPR
gp120 inner domain
outer domain
inner domain
gp41
C73/ C561
C72/ C564
E64K
A316W
less protected
no change
more protected
no data

Figure S4. HDX-MS profiles of PGT145 purified wild type and stabilized BG505 SOSIP trimers. Related to Fig. 1. Butterfly plots comparing the HDX-MS profiles of various purified BG505 trimers. (**A**) SOSIP.664 A73C-A561C; (**B**) SOSIP.v5.2; (**C**) SOSIP.v5.1. (**D-F**) the same trimers as in **A-C** but with sCD4 present. The percent exchange for each observable peptide is plotted at the position of the center of the peptide on the primary sequence for each time point (3 s to 20 h). The difference plots below each primary plot reveal regions undergoing slower exchange (more protected, above the zero) and faster exchange (less protected, below the zero). Differences are mapped onto one lobe of the trimer crystal structure (PDB: 4VTP, Pancera et al., 2014). Segments unresolved in the crystal structure (V2, V4, and a portion of gp41) are shown as dashed lines. Point mutations are indicated and shown as green spheres.

Figure S5

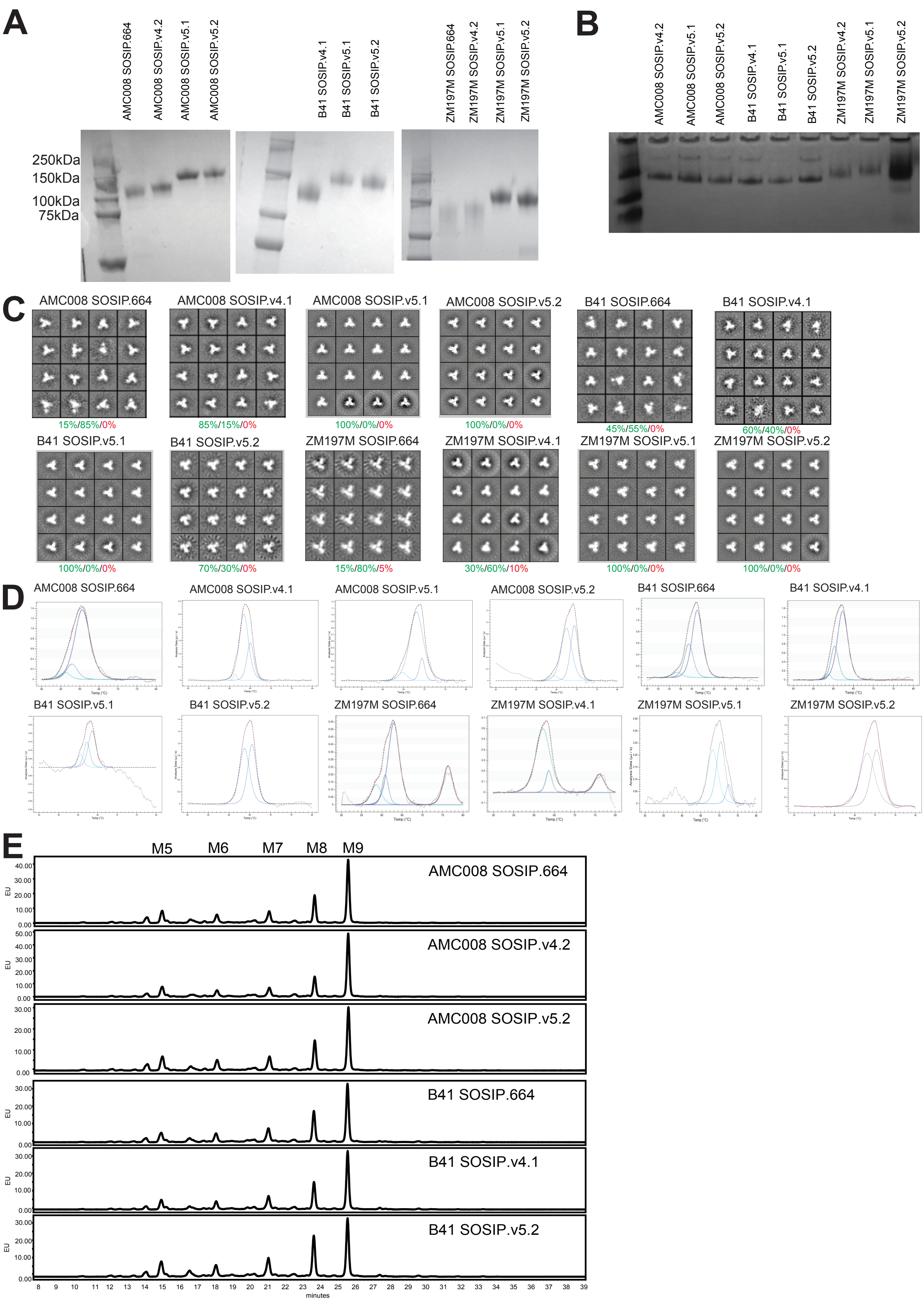

A
AMC008 SOSIP.664
AMC008 SOSIP.v4.2
AMC008 SOSIP.v5.1
AMC008 SOSIP.v5.2
B41 SOSIP.v4.1
B41 SOSIP.v5.1
B41 SOSIP.v5.2
ZM197M SOSIP.664
ZM197M SOSIP.v4.2
ZM197M SOSIP.v5.1
ZM197M SOSIP.v5.2
250kDa
150kDa
100kDa
75kDa
B
AMC008 SOSIP.v4.2
AMC008 SOSIP.v5.1
AMC008 SOSIP.v5.2
B41 SOSIP.v4.1
B41 SOSIP.v5.1
B41 SOSIP.v5.2
ZM197M SOSIP.v4.2
ZM197M SOSIP.v5.1
ZM197M SOSIP.v5.2
C
AMC008 SOSIP.664
15%/85%/0%
AMC008 SOSIP.v4.1
85%/15%/0%
AMC008 SOSIP.v5.1
100%/0%/0%
AMC008 SOSIP.v5.2
100%/0%/0%
B41 SOSIP.664
45%/55%/0%
B41 SOSIP.v4.1
60%/40%/0%
B41 SOSIP.v5.1
100%/0%/0%
B41 SOSIP.v5.2
70%/30%/0%
ZM197M SOSIP.664
15%/80%/5%
ZM197M SOSIP.v4.1
30%/60%/10%
ZM197M SOSIP.v5.1
100%/0%/0%
ZM197M SOSIP.v5.2
100%/0%/0%
D
AMC008 SOSIP.664
AMC008 SOSIP.v4.1
AMC008 SOSIP.v5.1
AMC008 SOSIP.v5.2
B41 SOSIP.664
B41 SOSIP.v4.1
B41 SOSIP.v5.1
B41 SOSIP.v5.2
ZM197M SOSIP.664
ZM197M SOSIP.v4.1
ZM197M SOSIP.v5.1
ZM197M SOSIP.v5.2
E
M5
M6
M7
M8
M9
AMC008 SOSIP.664
AMC008 SOSIP.v4.2
AMC008 SOSIP.v5.2
B41 SOSIP.664
B41 SOSIP.v4.1
B41 SOSIP.v5.2
EU
minutes
8 9 10 11 12 13 14 15 16 17 18 19 20 21 22 23 24 25 26 27 28 29 30 31 32 33 34 35 36 37 38 39

Figure S5. Biochemical biophysical and antigenic characterization of PGT145-purified stabilized clade-B (AMC008 and B41) and clade C (ZM197M) SOSIP.664 trimer variants. Related to Table 1 and Table 2. (**A**) Cleavage efficiency and disulfide bond formation was determined by SDS-PAGE under reducing and non-reducing conditions, respectively. Under reducing conditions, all proteins yielded a gp120 band, indicating that they were completely cleaved. Under non-reducing conditions the proteins all migrated as a gp140 band, but the mutants containing two disulfide bonds migrated more slowly through the gels. (**B**) The various subtype B (AMC008 and B41) and subtype C (ZM197M) proteins, purified by PGT145 affinity chromatography, were exclusively trimeric as determined by BN-PAGE. (**C**) The formation of native-like trimers was assessed by NS-EM. The 2D reference free class averages for the two double disulfide bond proteins (SOSIP.v5) compared to SOSIP.664 are shown. The percentages of closed native-like and open native-like trimers (Pugach et al., 2015) are shown in green and the percentage of non-native trimers in red. (**D**) Thermal stability was measured by DSC for the various D7324-tagged AMC008, B41 and ZM197M trimers. The independent non-two state best-fit curves are depicted in dashed red line and the T_m values of each peak are given in the graphs. See the Materials and Methods section for more details on curve modeling. (**E**) Glycan profiles of AMC008, B41 and ZM197M trimer variants were determined by HILIC-UPLC. $Man_{5\text{-}9}GlcNAc_2$ glycans are indicated as M5-M9 above the panel.

Table S1. Nomenclature of SOSIP trimers. Modifications present in each SOSIP trimer design are in green, with red indicating their absence. Related to Fig. 1.

			A501C-T605C	I559P	R6	MPER	I535M	L543Q	L543N	A316W	E64K	H66R	H72C-H564C	A73C-A561C	E49C-L555C
**SOSIP.681**	SOSIP.v1	SOSIP.v1													
**SOSIP.664**	SOSIP.v2	SOSIP.v2													
	**SOSIP.v3**	SOSIP.v3.1													
		SOSIP.v3.2													
	**SOSIP.v4**[a]	SOSIP.v4.1													
		SOSIP.v4.2													
	**SOSIP.v5**	SOSIP.v5.1													
		SOSIP.v5.2													
	**SOSIP.v6**	SOSIP.v6													

[a] Described in de Taeye et al., 2015.

Table S2. Biophysical properties of BG505 SOSIP.664 trimers. Related to Fig. 1 and Table 1.

	Introduced mutations/ SOSIP version	**DLS** [a]		**SAXS** [a]	
		R_h (Å)[a]	Pd (%)	Rg (Å) (Guinier) [b]	Rg (Å) (GNOM) [c]
BG505	SOSIP.664	69.2	2.6	52.8	52.5
	SOSIP.664 H72C-H564C	ND	ND	ND	ND
	SOSIP.664 A73C-A561C	68.8	2.0	52.8	51.7
	SOSIP.v4.1	68.7	3.3	53.0	51.9
	SOSIP.v5.1	67.8	3.6	52.1	51.7
	SOSIP.v5.2	67.2	4.4	54.6	51.8

[a] DLS and SAXS analysis were performed in stabilized SOSIP trimer variants.
[b] Standard deviation differs in +/- 0.3-0.5Å.
[c] Standard deviation is +/- 0.2.

Table S3. X-ray data collection and refinement statistics. Related to Fig. 1.

	BG505 SOSIP.664 72C-564C + PGT122 + 35O22 + NIH45-46
Beamline	APS 23-ID-D
# Crystals	1
Wavelength, Å	1.03317
Space group	C2
Unit cell a, b, c (Å)	361.8, 215.9, 176.6
α, β, γ (°)	90, 114.0, 90
Resolution (Å)	40.0 – 7.0 (7.1 – 7.0)
Completeness	99.3 (99.9)
Redundancy	7.6 (8.1)
No. total reflections	149,758
No. unique reflections	19,551
I/σ	7.2 (1.7)
$CC_{1/2}$	99.4 (44.8)
R_{sym}	0.20 (0.88)
R_{pim}	0.08 (0.33)
Resolution (Å)	40.0 – 7.0
No. reflections total/R_{free}	19,539/978
R_{cryst}/R_{free}	0.33/0.34
RMSD bond length (Å)	0.02
RMSD bond angles (°)	1.2
Protein atoms/Glycan atoms	42,237
Wilson B-value (Å^2)	278
Overall average B-value (Å^2)	367
Ramachandran allowed %	99.0
MolProbity all-atom clashscore	10.4
PDB ID	5WDU

Table S5. Disulfide bond formation in BG505 SOSIP variants. Related to Fig. 1 and Table 1. Location of disulfide bonds was assessed by MS-MS-UPLC. CS is Charged State. NA is not applicable.

Disulfide Loop Domain	Disulfide-Linked Peptides	BG505 SOSIP.664 His tag				
		CS	Theoretical m/z	Experimental m/z	Mass Error (ppm)	MS/MS
I	DAETTLFC^{54}ASDAK<br>HNVWATHAC^{74}VPTDPNPQEIHLED^{88}VTEEFNMWK	3+	1719.4427	1719.4495	4	✓
		4+	1289.8338	1289.8410	6	
		5+	1032.0685	1032.0750	6	
		6+	860.2250	860.2331	10	
		7+	737.4796	737.4832	5	
II	NNMVEQMHTDIISLWDQSLKPC^{119}VK<br>LINC^{196}D^{197}TSAITQAC^{205}PK<br>LTPLC^{126}VTLQC^{131}TD^{132}VTND^{136}ITDDMR<br>D^{156}C^{157}SFD^{160}MTTELR	7+	1169.8301	1169.8358	5	✓
		8+	1023.7272	1023.7337	6	
		9+	910.0917	910.0940	3	
		10+	819.1832	819.1869	5	
III	C^{228}K<br>FN^{234}GTGPC^{239}PSVSTVQC^{247}THGIKPVVSTQLLLN^{262}GSLAEEEVMIR<br>VSFEPIPIHYC^{218}APAGFAILK	5+	1346.6868	1346.6867	0.1	✓
		6+	1122.4069	1122.4067	0.2	
		7+	962.2069	962.2082	1	
		8+	842.0570	842.0570	0	
IV	NILVQFNTPVQID^{295}C^{296}TRPND^{301}NTR<br>QAHC^{331}D^{332}VSK	2+	1721.8332	1721.8347	1	✓
		3+	1148.2245	1148.2295	4	
		4+	861.4202	861.4246	5	
		5+	689.3376	689.3425	7	
		6+	574.6159	574.6182	4	
V	FADSSGGDLEVTTHSFNC^{378}GGEFFYC^{385}DTSGLFDSTWISDTSVQGSDSTGSDDSITLPC^{418}R<br>C^{445}VSDITGLILTR	4+	1852.5669	1852.5723	3	✓
		5+	1482.2549	1482.2632	6	
		6+	1235.3803	1235.3881	6	
gp41 SOSIP	C^{501}K<br>LIC^{604}C^{605}TNVPWN^{611}SSWSNR<br>DQQLLGIWGC^{598}SGK	3+	1176.8808	1176.8860	4	✓
		4+	882.9124	882.9167	5	
		5+	706.5314	706.5352	5	
		6+	588.9440	588.9460	3	

Disulfide Loop Domain	Disulfide-Linked Peptides	BG505 SOSIP.v5.2 His tag				
		CS	Theoretical m/z	Experimental m/z	Mass Error (ppm)	MS/MS
I	DAETTLFC^{54}ASDAK<br>HNVWATHAC^{73}C^{74}VPTDPNPQEIHLED^{88}VTEEFNMWK<br>APEC^{561}QQHLLK	4+	1588.4748	1588.4701	3	✓
		5+	1270.9813	1270.9789	2	
		6+	1059.3189	1059.3171	2	
		7+	908.1316	908.1304	1	
		8+	794.7410	794.7401	1	
II	NNMVEQMHTDIISLWDQSLKPC^{119}VK*<br>LINC^{196}D^{197}TSAITQAC^{205}PK<br>LTPLC^{126}VTLQC^{131}TN^{132}VTND^{136}ITDDMR*<br>D^{156}C^{157}SFD^{160}MTTELR*	6+	1375.1331	1375.1414	6	✓
		7+	1178.8294	1178.8367	6	
		8+	1031.6017	1031.6061	4	
		9+	917.0912	917.0961	5	
		10+	825.4828	825.4881	6	
III	C^{228}K<br>FN^{234}GTGPC^{239}PSVSTVQC^{247}THGIKPVVSTQLLLN^{262}GSLAEEEVMIR<br>VSFEPIPIHYC^{218}APAGFAILK	5+	1346.6868	1346.6826	3	✓
		6+	1122.4069	1122.4022	4	
		7+	962.2069	962.2042	3	
		8+	842.0570	842.0552	2	
		9+	748.6070	748.6050	3	
IV	NILVQFNTPVQID^{295}C^{296}TRPND^{301}NTR<br>QAHC^{331}D^{332}VSK	2+	1721.8332	1721.8383	3	✓
		3+	1148.2245	1148.2256	1	
		4+	861.4202	861.4220	3	
		5+	689.3376	689.3415	6	
		6+	574.6159	574.6183	4	
V	FADSSGGDLEVTTHSFNC^{378}GGEFFYC^{385}DTSGLFDSTWISDTSVQGSDSTGSDDSITLPC^{418}R<br>C^{445}VSDITGLILTR	4+	1852.5669	1852.5714	3	✓
		5+	1482.2549	1482.2606	4	
		6+	1235.3803	1235.3863	5	
gp41 SOSIP	C^{501}K<br>LIC^{604}C^{605}TNVPWN^{611}SSWSNR<br>DQQLLGIWGC^{598}SGK	3+	1176.8808	1176.8824	1	✓
		4+	882.9124	882.9155	4	
		5+	706.5314	706.5343	4	
		6+	588.9440	588.9454	2	

Disulfide Loop Domain	Disulfide-Linked Peptides	BG505 SOSIP.v5.2 I201C-A433C His tag				
		CS	Theoretical *m/z*	Experimental *m/z*	Mass Error (ppm)	MS/MS
I	DAETTLFC^{54}ASDAK<br>HNVWATHAC^{73}C^{74}VPTDPNPQEIHLED^{88}VTEEFNMWK<br>APEC^{561}QQHLLK	4+	1588.4748	1588.4761	1	✓
		5+	1270.9813	1270.9756	5	
		6+	1059.3189	1059.3146	4	
		7+	908.1316	908.1285	3	
		8+	794.7410	794.7391	2	
II	IGC^{433}GQAMYAPPIQGVIR* NNMVEQMHTDIISLWDQSLKPC^{119}VK*<br>LINC^{196}D^{197}TSAC^{201}TQAC^{205}PK<br>LTPLC^{126}VTLQC^{131}TN^{132}VTND^{136}ITDDMR*<br>D^{156}C^{157}SFD^{160}MTTELR*	7+	1414.5061	1414.5113	6	✓
		8+	1237.8187	1237.8262	4	
		9+	1100.3952	1100.3992	5	
		10+	990.4564	990.4588	6	
III	C^{228}K<br>FN^{234}GTGPC^{239}PSVSTVQC^{247}THGIKPVVSTQLLLN^{262}GSLAEEEVMIR<br>VSFEPIPIHYC^{218}APAGFAILK	5+	1346.6868	1346.6850	3	✓
		6+	1122.4069	1122.4051	4	
		7+	962.2069	962.2031	3	
		8+	842.0570	842.0570	2	
IV	NILVQFNTPVQID^{295}C^{296}TRPND^{301}NTR<br>QAHC^{331}D^{332}VSK	3+	1148.2245	1148.2255	1	✓
		4+	861.4202	861.4207	1	
		5+	689.3376	689.3392	2	
		6+	574.6159	574.6169	2	
V	FADSSGGDLEVTTHSFNC^{378}GGEFFYC^{385}DTSGLFDSTWISDTSVQGSDSTGSDDSITLPC^{418}R<br>C^{445}VSDITGLILTR	4+	1852.5669	1852.5686	1	✓
		5+	1482.2549	1482.2595	3	
		6+	1235.3803	1235.3865	5	
gp41 SOSIP	C^{501}K<br>LIC^{604}C^{605}TNVPWN^{611}SSWSNR<br>DQQLLGIWGC^{598}SGK	3+	1176.8808	1176.8805	0.3	✓
		4+	882.9124	882.9136	1	
		5+	706.5314	706.5323	1	
		6+	588.9440	588.9447	1	

Disulfide Loop Domain	Disulfide-Linked Peptides	BG505 SOSIP.v6 His tag				
		CS	Theoretical *m/z*	Experimental *m/z*	Mass Error (ppm)	MS/MS
I	NLLSGIVQQSNC^{555}LR<br>DAC^{49}TTLFC^{54}ASDAK<br>HNVWATHAC^{73}C^{74}VPTDPNPQEIHLED^{88}VTEEFNMWK<br>APEC^{561}QQHLLK	6+	1336.1194	1336.1263	5	✓
		7+	1145.3891	1145.3919	3	
		8+	1002.3413	1002.3442	3	
		9+	891.0820	891.0833	2	
		10+	802.0745	802.0776	4	
II	NNMVEQMHTDIISLWDQSLKPC^{119}VK*<br>LINC^{196}D^{197}TSAITQAC^{205}PK<br>LTPLC^{126}VTLQC^{131}TD^{132}VTND^{136}ITDDMR*<br>D^{156}C^{157}SFD^{160}MTTELR*	6+	1375.2971	1375.3055	6	✓
		7+	1178.9700	1178.9772	6	
		8+	1031.7247	1031.7313	6	
		9+	917.2005	917.2061	6	
		10+	825.5812	825.5874	8	
III	C^{228}K<br>FN^{234}GTGPC^{239}PSVSTVQC^{247}THGIKPVVSTQLLLN^{262}GSLAEEEVMIR<br>VSFEPIPIHYC^{218}APAGFAILK	5+	1346.6868	1346.6806	5	✓
		6+	1122.4069	1122.4022	4	
		7+	962.2069	962.2032	4	
		8+	842.0570	842.0535	4	
		9+	748.6070	748.6044	4	
IV	NILVQFNTPVQID^{295}C^{296}TRPND^{301}NTR<br>QAHC^{331}D^{332}VSK	3+	1148.2245	1148.2257	1	✓
		4+	861.4202	861.4220	2	
		5+	689.3376	689.3401	4	
		6+	574.6159	574.6174	3	
V	FADSSGGDLEVTTHSFNC^{378}GGEFFYC^{385}DTSGLFDSTWISDTSVQGSDSTGSDDSITLPC^{418}R<br>C^{445}VSDITGLILTR	4+	1852.5669	1852.5607	5	✓
		5+	1482.2549	1482.2617	4	
		6+	1235.3803	1235.3852	3	
gp41 SOSIP	C^{501}K<br>LIC^{604}C^{605}TNVPWN^{611}SSWSNR<br>DQQLLGIWGC^{598}SGK	3+	1176.8808	1176.8837	3	✓
		4+	882.9124	882.9151	3	
		5+	706.5314	706.5342	4	
		6+	588.9440	588.9458	3	

Disulfide Loop Domain	Disulfide-linked Peptides	BG505 SOSIP.664	BG505 SOSIP.v5.2	BG505 SOSIP.v5.2 I201C-A433C	BG505 SOSIP.v6
I	HNVWATHAC73C74VPTDPNPQEIHLED88VTEEFNMWK	NA	Trace	✗	✗
	DAC49TTLFC54ASDAK	NA	NA	NA	✓
II	LINC196D197TSAITQAC205PK	Trace	Trace	NA	✓
	LTPLC126VTLQC131TD132VTND136ITDDMR	✓	Trace	Trace	✓
III	C228K<br>VSFEPIPIHYC218APAGFAILK	Trace	✗	✗	Trace
	FD234GTGPC239PSVSTVQC247THGIKPVVSTQLLLD262GSLAEEEVMIR	✓	✗	Trace	✓
gp41 SOSIP	LIC604C605TNVPWN611SSWSNR	✓	✓	✓	✓

Supplemental Experimental Procedures

Construct design

The constructs expressing BG505, B41, AMC008 and ZM1097M SOSIP.664 proteins have been described elsewhere (Julien et al., 2015; Pugach et al., 2015; Sanders et al., 2013; de Taeye et al., 2015). To improve the formation of soluble trimers, these constructs contained the following changes compared to the original Env sequence: a TPA signal sequence; A501C and T605C; I559P; REKR to RRRRRR at the C-terminus of gp120; a stop codon after residue 664 (Binley et al., 2000, 2002; Khayat et al., 2013; Klasse et al., 2013; Sanders et al., 2002). In addition, we introduced substitutions to restore glycan-dependent bNAb epitopes: T332N in BG505 and D156N, E295N and D332N in ZM197M (Julien et al., 2015; Pugach et al., 2015; Sanders et al., 2013; de Taeye et al., 2015). We refer to these constructs as SOSIP.664. We further introduced the SOSIP.v4 mutations, i.e. E64K or H66R and A316W trimer-stabilizing changes (de Taeye et al., 2015), as well as the I/V535M and L543N trimer-improving changes to gp41, where necessary (de Taeye et al., 2015). We also constructed AMC008, B41 and ZM197M trimer variants bearing either a His-tag (BG505) or a D7324 epitope-tag sequence at the C-terminus after residue 664 (GSGSGGSGHHHHHHHH or GSAPTKAKRRVVQREKR, respectively), as described in Sanders et al. 2013. Point mutations were made using Quickchange site-directed mutagenesis kit (Agilent Technologies, La Jolla, CA, USA) and constructs were verified by sequencing prior to use.

Protein expression and purification

The Env proteins were transiently expressed in adherent 293T cells or suspension 293F cells in the presence of excess co-transfected *furin* and purified using PGT145-affinity chromatography as described previously (Julien et al., 2013a; Julien et al., 2015; Pugach et al., 2015; Sanders et al., 2013; de Taeye et al., 2015). All of the exploratory experiments using unpurified Env from supernatant of transfected cells made use of 293T cell-expressed proteins, while purified trimers were derived from 293F cells. SDS-PAGE and BN-PAGE analyses were performed as previously described (Julien et al., 2013a; Pugach et al., 2015; Sanders et al., 2013; de Taeye et al., 2015).

Neutralization assays

The TZM-bl cell line, which expresses high levels of CD4, CCR5 and CXCR4 and contains beta-galactosidase and luciferase genes under the control of the HIV-1 long terminal repeat promoter, was used for neutralization assays. We used Env-pseudotyped or chimeric molecular clone viruses to perform the neutralization assays at DUMC, Duke University Medical Center, AMC, Academic Medical Center, and WCMC, Weill Medical College of Cornell University. For methodology, see https://www.hiv.lanl.gov/content/nab-reference-strains/html/Protocol-for-Neutralizing-Antibody-Screening-Assay-for-HIV-1-in-TZMbl-cells_Jan2016.pdf; Sanders et al., 2013 and Klasse et al., 2016, respectively.

SDS-PAGE and Blue Native-PAGE

The presence of Env trimer was analyzed using SDS-PAGE and BN-PAGE followed by western blot or Coomassie blue dye staining according to previous protocols described in Sanders et al. 2002 and Schülke et al. 2002. In summary, the input material, Env-containing supernatant or purified trimer, was mixed with loading buffer (for SDS-PAGE: 25mM Tris, 192mM glycine, 20% v/v glycerol, 4% m/v SDS, 0.1% v/v bromophenol blue in milli-Q water; for Native-PAGE: 500µl 20x MOPS buffer, 1000µl 100% ultrapure glycerol, 50µl Coomassie brilliant blue and 600µl milli-Q water). For SDS-PAGE gels, the samples were incubated at 99°C for 5min with or without DTT (100mM) and 4-12% Tris-Glycine gels were run at 125V for 2h using running buffer (Invitrogen). For Native-PAGE, the samples were directly loaded onto a 4-12% Bis-Tris NuPAGE gel and the gel was run at 200V for 2h using Anode and Cathode-buffer (Invitrogen). Western Blot was performed using Arp3119 followed by HRP-labeled goat anti-mouse or 2G12 MAb (0.1µg/ml) followed by HRP-labeled goat anti-human IgG for SDS-PAGE and Native-PAGE, respectively. Coomassie blue staining was performed using PageBlue Protein Staining Solution (Thermo Scientific) and Colloidal Blue Staining Kit (Life Technologies) for SDS-PAGE and Native-PAGE, respectively.

Surface Plasmon Resonance (SPR), ELISA and thermostability ELISA

SPR analyses were performed with His-tagged trimers immobilized by anti-His antibody covalently linked to CM5 chips as previously described (Derking et al., 2015; Yasmeen et al., 2014).

D7324-capture and Ni-NTA capture ELISAs have been described elsewhere (Derking et al., 2015; Sanders et al., 2013). Briefly, for D7324-capture ELISA microloan-600, half-area plates (Greiner Bio-One) were coated overnight with D7324 antibody at 10μg/ml in 0.1M $NaHCO_3$, pH 8.6. After washing and blocking the D7324 antibody, the trimers were added at 2.2 μg/ml in TBS for 2h. After washing the unbound trimers with TBS, the different antibodies were added serially diluted. After washing with TBS, the HRP-labeled goat anti-human IgG (Jackson Immunoresearch) was added for 45 minutes. The colorimetric detection was performed with develop solution (1% 3,3',5,5'-tetramethylbenzidine, 0.01% H_2O_2, 100mM sodium acetate and 100mM citric acid) after five washes with TBS/0.05% tween-20). The colorimetric reaction was stopped with 0.8M H_2SO_4 when a plateau was reached. For Ni-NTA capture ELISA, Ni-HisSorb plates (Qiagen) were used. Trimers were directly added to the plate at a concentration of 0.6μg/ml. Detection was done using broadly neutralizing antibodies serially diluted.

ELISA-based thermostability ELISA was performed with unpurified BG505 His-tagged mutants as previously described (de Taeye et al. 2015). In short, 100μl of unpurified supernatants were incubated for 30 mins at different temperatures ranging between 39 and 77°C using a G-storm PCR machine (GRI Lab Care). Supernatants were transferred to a 96-well Ni-NTA plate and a Ni-NTA capture ELISA was performed following the described protocol above. The detection was done using the 2G12 antibody at 0.1μg/ml.

Differential scanning calorimetry (DSC)

To probe the thermostability of SOSIP.664 trimers, we used a Nano-DSC (TA Instruments, New Castle, DE, USA) and analyzed the data with NanoAnalyze Software v.3.3.0 (TA Instruments). The data were fitted using an independent non-two-state model, as the asymmetry of some of the peaks suggested the presence of unfolding intermediates. However, for simplification, we also analyzed the data using a two-state scaled model. We report the T_m values derived from the two-state scaled model in the main manuscript. All of the DSC data were derived using tagged trimers. When some comparative studies were performed, we found that the T_m values obtained with His-tagged trimers were consistently ~0.9-1.0°C higher than those for the same trimers without tag (data not shown), and 0.6°C higher than those for D7324-tagged trimers (de Taeye et al., 2015). For example, PGT145-purified His-tagged BG505 SOSIP.664 trimers have a T_m of 67.6°C, while the corresponding non-tagged trimers have a T_m of 66.7°C (data not shown), and the D7324-tagged versions have a T_m of 67.1°C (de Taeye et al., 2015). The implication is that the His-tag has a modest stabilizing effect on the trimer.

Analysis of total glycan profiles by HILIC-UPLC

N-linked glycans were enzymatically released from Env glycoproteins by in-gel digestion with Peptide-N-Glycosidase F (PNGase F), subsequently fluorescently labelled with 2-aminobenzoic acid (2-AA) and analyzed by HILIC-UPLC (Hydrophilic Interaction Liquid Chromatography – Ultra Performance Liquid Chromatography), as previously described (Behrens et al., 2016; Neville et al., 2009; Pritchard et al., 2015). Digestion of released glycans with Endoglycosidase H (Endo H) was used to determine the abundance of oligomannose-type glycans (Pritchard et al., 2015).

Negative-stain electron microscopy

Purified Env trimers were analyzed by negative-stain EM. A 3 μl aliquot containing ~0.03 mg/mL of the trimer was applied for 5 s onto a carbon-coated 400 Cu mesh grid that had been glow discharged at 20 mA for 30 s, then negatively stained with uranyl formate for 30 s. Data were collected using a FEI Tecnai F20 or T12 electron microscope operating at 120 keV, with an electron dose of ~55 $e^-/Å^2$ and a magnification of 52,000x that resulted in a pixel size of 2.05Å at the specimen plane. Images were acquired with a Gatan US4000 CCD or Tietz TemCam-F416 CMOS camera using a nominal defocus range of 900 to 1300 nm.

Image processing

Particles were picked automatically using DoG Picker and put into a particle stack using the Appion software package. Initial, reference-free, two-dimensional (2D) class averages were calculated using particles binned by five via the Xmipp Clustering 2D Alignment and sorted into classes. Particles

corresponding to trimers were selected into a substack and binned by four before another round of reference-free alignment was carried out using the Xmipp Clustering and 2D alignment and IMAGIC software systems.

Small Angle X-ray Scattering (SAXS)

SAXS measurements were conducted on Beam Line 4-2 at the Stanford Synchrotron Radiation Lightsource (Smolsky et al., 2007). The focused 11 keV X-ray beam irradiated a thin-wall quartz capillary cell, placed at 2.5 m upstream of the MX 225HE detector (Rayonix, Evanston, IL, USA). Aliquots containing 50 µl of BG505 constructs (1 – 2 mg/ml) were injected onto a high resolution Sepharose 200 column (GE Healthcare, Wilmington, MA, USA) with a flow rate of 50 µl/min in a buffer comprising 20 mM Na_3PO_4 pH 7.4, 150 mM NaCl, 0.02% NaN_3, 1 mM EDTA. The flow from the column passed through a UV detector cell and into the quartz capillary cell. Exposures of 1 s duration were collected every 5 s throughout the run, with a circulating water bath maintaining the capillary cell temperature at 8°C. The detector pixel numbers were radially integrated and scaled as described previously (de Taeye et al., 2015). The radius of gyration (R_g) and scattering at zero angle [I(0)] parameters for each frame were batch analyzed using autoRg, and frames with stable R_g values were merged *in primus* for the final scattering curve (Petoukhov et al., 2007). Real space distance distribution functions were calculated from the merged data sets using GNOM (Svergun, 1992).

Dynamic Light Scattering (DLS)

Proteins in PBS at 1 - 2 mg/ml were centrifuged at 15000g for 10 min at 4°C immediately before light scattering measurements. Data were collected on a Dynapro instrument (Wyatt Technologies, Goleta, CA, USA), with 30 acquisitions of 10 s each at 20°C, and analyzed with the manufacturer's software (Dynamics, Wyatt Technologies). We note that the hydrodynamic radius (R_h) of 6.9 nm derived using DLS for BG505 SOSIP.664 trimers that we report here and elsewhere (Table S2; de Taeye et al., 2015) is slightly lower than the previously reported value of 8.1 nm (Julien et al., 2013b). The latter value was derived using flow mode SEC coupled with static and quasi-elastic light scattering detectors, and fitted using a monomodal model. Calculated hydrodynamic radius values derived using this method represent the mean and distribution of diffusion constants.

X-ray crystallography

The BG505 SOSIP.664 72C-564C trimer was expressed and purified as previously described (Julien et al. 2013b). Purified trimers were mixed with a molar excess of the PGT122 Fab and 35O22 Fab and treated with EndoH (New England BioLabs). Subsequently, the complex was mixed with NIH45-46 scFv in molar excess and the quaternary complex was purified to size homogeneity using a Superose 6 10/30 gel filtration column (GE Healthcare). The complex was set up for crystallization trials at a protein concentration of 4 mg/ml, using an Oryx8 crystallization robot (Douglas Instruments). Crystals grew in sitting drop experiments from a condition containing 8% (w/v) polyethylene glycol 8000, 0.1 M Tris, pH 8.5. A complete dataset was obtained to 7.0 Å resolution from a single flash-cooled crystal that was cryo-protected with 30% glycerol. Data were processed using XDS (Kabsch, 2010), and data collection and processing statistics are reported in Table S3. For molecular replacement in PHASER (McCoy et al., 2007), a hybrid model was generated using the BG505 SOSIP.664 trimer + PGT122 Fab + 35022 Fab from PDB ID: 4TVP and NIH45-46 scFv superposed from PDB ID: 5D9Q. The resulting hybrid model was used as the search model. Iterative rigid body and grouped B-factor refinements were carried-out with non-crystallographic symmetry (NCS) in PHENIX and inspected in COOT (Adams et al., 2010; Emsley and Cowtan, 2004). Refinement statistics are summarized in Table S3.

Hydrogen-Deuterium Exchange (HD-X)

Soluble two-domain CD4 (2D-sCD4) (Garlick et al., 1990) was obtained from the NIH. All proteins were SEC-purified using a Superdex S200 column (GE Healthcare) and a PBS buffer (20 mM sodium phosphate pH 7.4, 150 mM NaCl, 1 mM EDTA, 0.02 % sodium azide) and concentrated to 1 mg/mL immediately before HDX-MS analysis. Complexes were formed by an overnight incubation at 4°C with 2D-sCD4, which was present at a 3-fold molar excess relative to each protomer of the trimer. Native gels were run for each sample to monitor sCD4-trimer complex formation. BG505 trimers (15 µg) were diluted 10-fold into deuterated PBS buffer at room temperature. After incubation periods of 3 s, 1 min, 30 min, and 20 h, the exchange reactions were quenched by mixing with an equal volume of cold 200 mM

TCEP, 0.2% formic acid (final pH 2.5). The samples were subsequently digested with pepsin (0.15 mg/mL) for 5 min on ice, flash frozen in liquid nitrogen and stored at -80°C. Differences in deuterium exchange profiles that exceeded the error of the measurement were visualized on the trimer structure (PDB: 4TVP) using custom macros in PyMOL (DeLano, 2002).

Analysis of disulfide bond patterns in BG505 Env trimers

Disulfide bond patterns of BG505 Env trimers were determined by mapping the disulfide-linked peptides by mass spectrometry. Details of the sample preparation were described previously (Go et al., 2011, 2014). Briefly, about 20 μg of Env samples were alkylated with a 10-fold molar excess of 4-vinylpyridine in the dark for one hour at room temperature to cap free cysteine residues. Alkylated Env samples were subsequently deglycosylated with 500 U of PNGase F in 100 μL of 100 mM ammonium citrate buffer (pH 6.5) for one week at 37°C. The fully deglycosylated and alkylated samples were digested overnight with trypsin (protein to enzyme ratio of 30:1) at 37°C and were analyzed by LC-MS using an Orbitrap Velos Pro™ hybrid (Thermo Scientific, San Jose CA) mass spectrometer equipped with electron transfer dissociation (ETD) module coupled to an Acquity Ultra Performance Liquid Chromatography (UPLC®) system (Waters, Milford MA). About 5 μL (1 μg equivalent) of the tryptic digest was injected onto a C18 PepMap™ 300 column (300 μm i.d. x 15 cm, 300 Å; Thermo Scientific, Sunnyvale, CA) and the peptides were separated using a linear gradient starting from 3% B to 40% B gradient in 50 min, then 90% B in 10 min, and re-equilibration at 97% A for 10 min. LC-MS runs were performed with a flow rate of 5 μL/min using mobile phases consisting of solvent A: 99.9% HPLC-grade H_2O + 0.1% formic acid and solvent B: 99.9 % HPLC grade CH_3CN + 0.1% formic acid. Data were collected using the data-dependent mode with five most intense ions in a high resolution scan in the Orbitrap were subjected to alternating collision-induced dissociation (CID) and ETD in the linear ion trap to determine the disulfide connectivity. Data analysis was performed using Mascot search engine for peptides containing free cysteine residues and disulfide bond patterns were analyzed manually as described previously (Go et al., 2011, 2014).

Immunizations

Rabbits were immunized at week 0, 4 and 20 with 22 μg of Env trimer or gp120. Rabbit sera was taken at week 22 and assayed for autologous and cross-reactive antibody responses using the TZM-bl cell neutralization assay. Binding titers were assessed by performing D7324 capture ELISA with week 22 sera as described elsewhere (Sanders et al., 2013). The tier classification of the Env pseudotyped viruses have been described in deCamp et al., 2014.

Supplemental References

Adams, P.D., Afonine, P. V., Bunkóczi, G., Chen, V.B., Davis, I.W., Echols, N., Headd, J.J., Hung, L.W., Kapral, G.J., Grosse-Kunstleve, R.W., et al. (2010). PHENIX: A comprehensive Python-based system for macromolecular structure solution. Acta Crystallogr. Sect. D Biol. Crystallogr. *66*, 213–221.

Behrens, A.J., Vasiljevic, S., Pritchard, L.K., Harvey, D.J., Andev, R.S., Krumm, S.A., Struwe, W.B., Cupo, A., Kumar, A., Zitzmann, N., et al. (2016). Composition and antigenic effects of individual glycan sites of a trimeric HIV-1 envelope glycoprotein. Cell Rep. *14*, 2695–2706.

Binley, J.M., Sanders, R.W., Master, A., Cayanan, C.S., Wiley, C.L., Schiffner, L., Travis, B., Kuhmann, S., Burton, D.R., Hu, S.-L., et al. (2002). Enhancing the proteolytic maturation of human immunodeficiency virus type 1 envelope glycoproteins. J. Virol. *76*, 2606–2616.

Cavacini, L.A., Emes, C.L., Wisnewski, A. V, Power, J., Lewis, G., Montefiori, D., and Posner, M.R. (1998). Functional and molecular characterization of human monoclonal antibody reactive with the immunodominant region of HIV type 1 glycoprotein 41. AIDS Res. Hum. Retroviruses *14*, 1271–1280.

deCamp, A., Hraber, P., Bailer, R.T., Seaman, M.S., Ochsenbauer, C., Kappes, J., Gottardo, R., Edlefsen, P., Self, S., Tang, H., et al. (2014). Global panel of HIV-1 Env reference strains for standardized assessments of vaccine-elicited neutralizing antibodies. J. Virol. *88*, 2489–2507.

DeLano, W. (2002). Pymol: An open-source molecular graphics tool. CCP4 Newsl. Protein Crystallogr. *700*.

Emsley, P., and Cowtan, K. (2004). Coot: Model-building tools for molecular graphics. Acta Crystallogr. Sect. D Biol. Crystallogr. *60*, 2126–2132.

Garlick, R.L., Kirschner, R.J., Eckenrode, F.M., Tarpley, W.G., and Tomich, C.S. (1990). *Escherichia coli* expression, purification, and biological activity of a truncated soluble CD4. AIDS Res. Hum. Retroviruses *6*, 465–479.

Go, E.P., Zhang, Y., Menon, S., and Desaire, H. (2011). Analysis of the disulfide bond arrangement of the HIV-1 envelope protein CON-S gp140 ΔCFI shows variability in the V1 and V2 regions. J. Proteome Res. *10*, 578–591.
Go, E.P., Hua, D., and Desaire, H. (2014). Glycosylation and disulfide bond analysis of transiently and stably expressed clade C HIV-1 gp140 trimers in 293T cells identifies disulfide heterogeneity present in both proteins and differences in o-linked glycosylation. J. Proteome Res. *13*, 4012–4027.
Kabsch, W. (2010). *XDS*. Acta Crystallogr. Sect. D Biol. Crystallogr. *66*, 125–132.
Khayat, R., Lee, J.H., Julien, J.-P., Cupo, A., Klasse, P.J., Sanders, R.W., Moore, J.P., Wilson, I. A., and Ward, A.B. (2013). Structural characterization of cleaved, soluble HIV-1 envelope glycoprotein trimers. J. Virol. *87*, 9865–9872.
Klasse, P.J., Depetris, R.S., Pejchal, R., Julien, J.-P., Khayat, R., Lee, J.H., Marozsan, A.J., Cupo, A., Cocco, N., Korzun, J., et al. (2013). Influences on trimerization and aggregation of soluble, cleaved HIV-1 SOSIP envelope glycoprotein. J. Virol. *87*, 9873–9885.
McCoy, A.J., Grosse-Kunstleve, R.W., Adams, P.D., Winn, M.D., Storoni, L.C., and Read, R.J. (2007). Phaser crystallographic software. J. Appl. Crystallogr. *40*, 658–674.
Neville, D.C.A., Dwek, R.A., and Butters, T.D. (2009). Development of a single column method for the separation of lipid- And protein-derived oligosaccharides. J. Proteome Res. *8*, 681–687.
Petoukhov, M. V., Konarev, P. V., Kikhney, A.G., and Svergun, D.I. (2007). ATSAS 2.1 - Towards automated and web-supported small-angle scattering data analysis. J. Appl. Crystallogr. *40*, 223–228.
Ringe, R.P., Sanders, R.W., Yasmeen, A., Kim, H.J., Lee, J.H., Cupo, A., Korzun, J., Derking, R., van Montfort, T., Julien, J.-P., et al. (2013). Cleavage strongly influences whether soluble HIV-1 envelope glycoprotein trimers adopt a native-like conformation. Proc. Natl. Acad. Sci. U. S. A. *110*, 18256–18261.
Schülke, N., Vesanen, M.S., Sanders, R.W., Lu, M., Anselma, D.J., Villa, A.R., Paul, W., Parren, H.I., Binley, J.M., Roux, K.H., et al. (2002). Oligomeric and conformational properties of a proteolytically mature , immunodeficiency virus type 1 gp140 envelope glycoprotein. J. Virol. *76*, 7760.
Smolsky, I.L., Liu, P., Niebuhr, M., Ito, K., Weiss, T.M., and Tsuruta, H. (2007). Biological small-angle X-ray scattering facility at the Stanford Synchrotron Radiation Laboratory. J. Appl. Crystallogr. *40*, 453–458.
Svergun, D.I. (1992). Determination of the regularization parameter in indirect-transform methods using perceptual criteria. J. Appl. Crystallogr. *25*, 495–503.
Yasmeen, A., Ringe, R., Derking, R., Cupo, A., Julien, J.P., Burton, D.R., Ward, A.B., Wilson, I.A., Sanders, R.W., Moore, J.P., et al. (2014). Differential binding of neutralizing and non-neutralizing antibodies to native-like soluble HIV-1 Env trimers, uncleaved Env proteins, and monomeric subunits. Retrovirology *11*, 41.
