# Supplementary figures and images for: Improving the Immunogenicity of Native-like HIV-1 Envelope Trimers by Hyperstabilization
Source: Cell Rep. 2017 Aug 23;20(8):1805–17. doi: 10.1016/j.celrep.2017.07.077 (PMC5590011; doi:10.1016/j.celrep.2017.07.077)

# Data S1

A

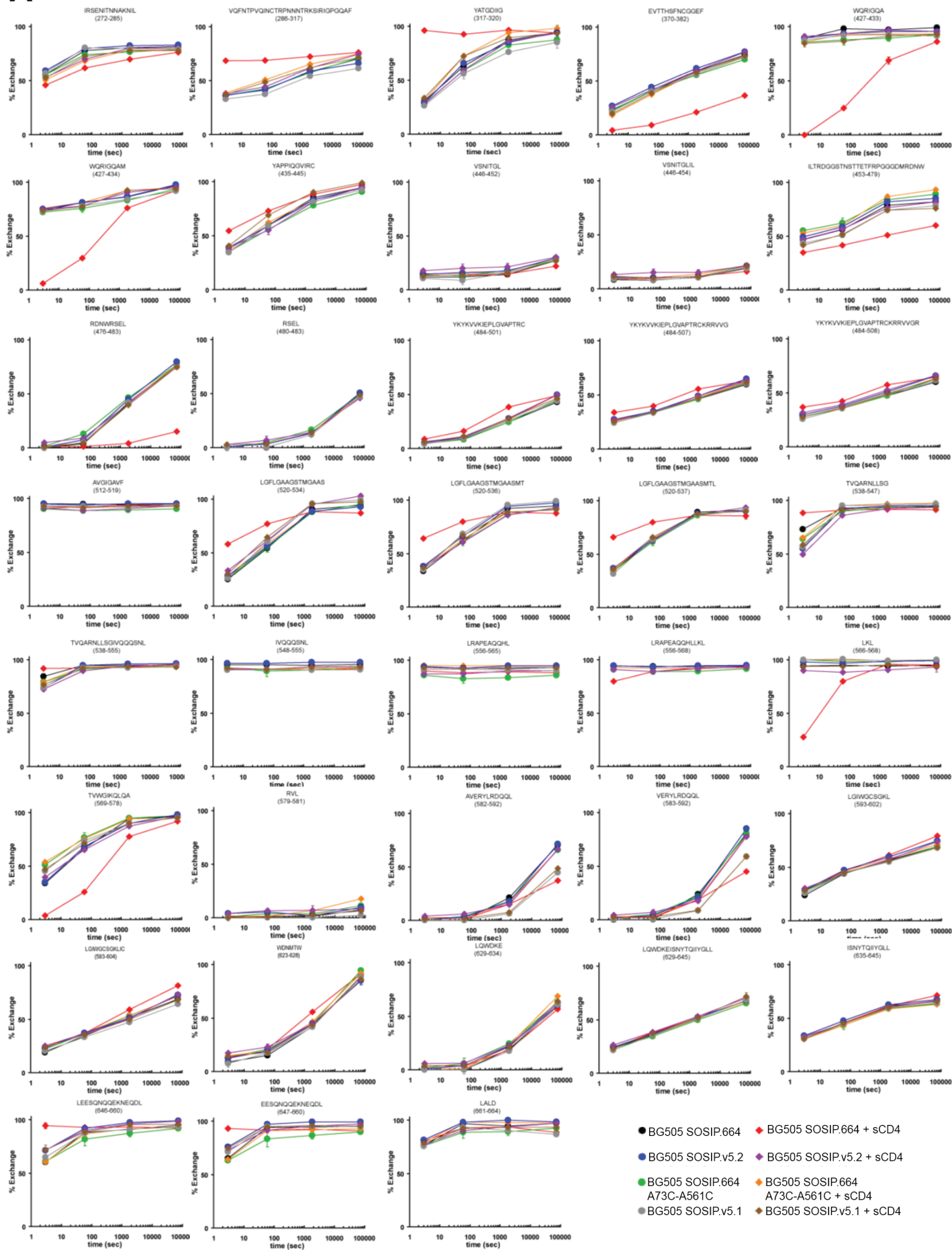

# Data S1

A

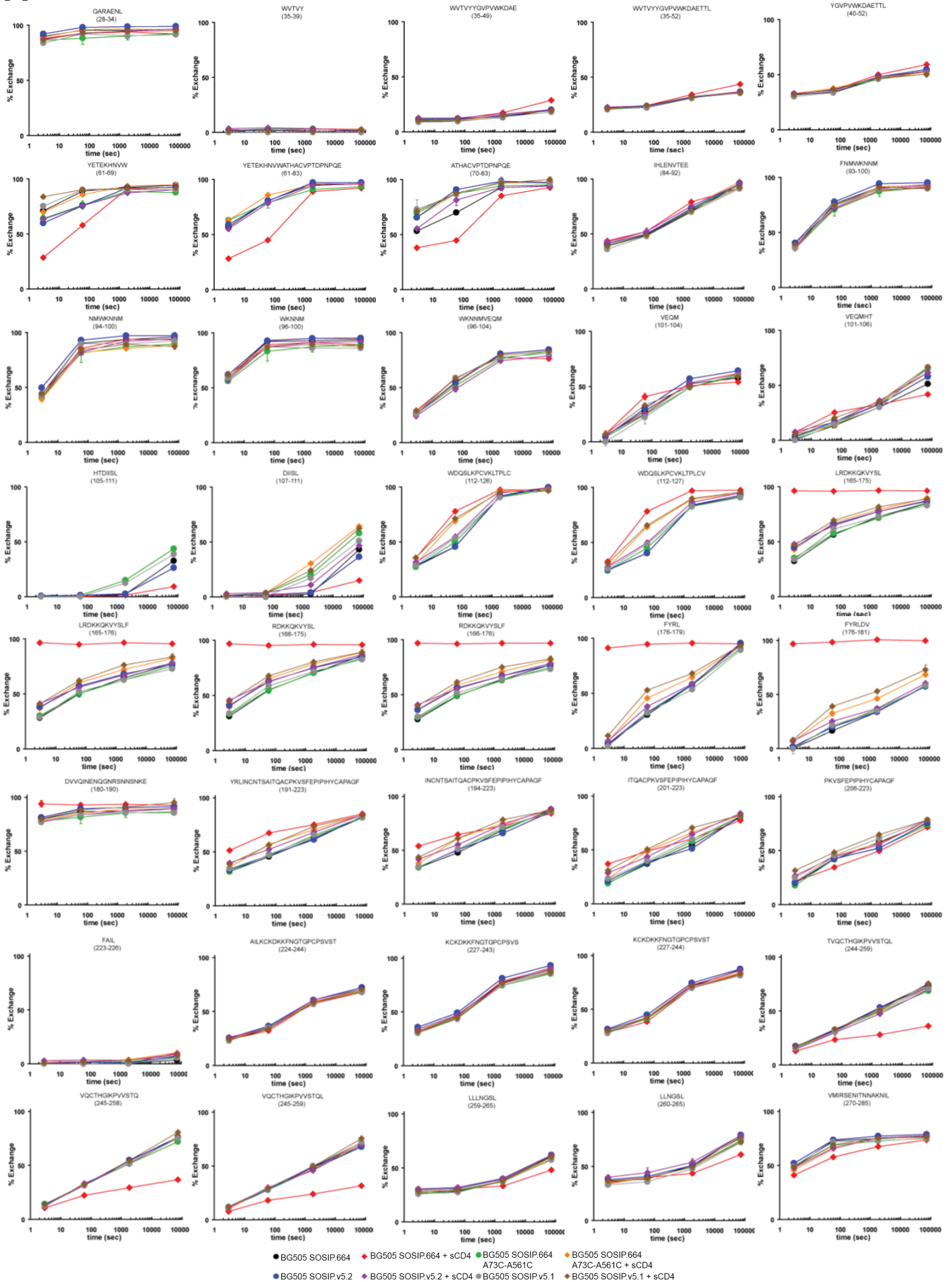

Data S1

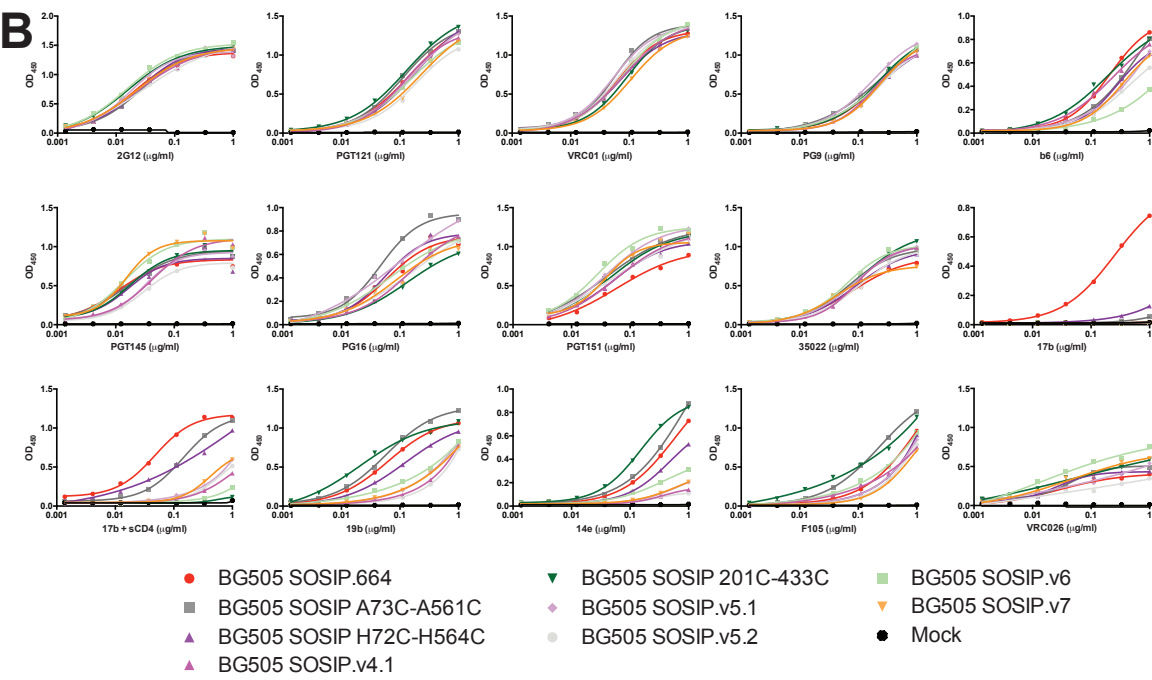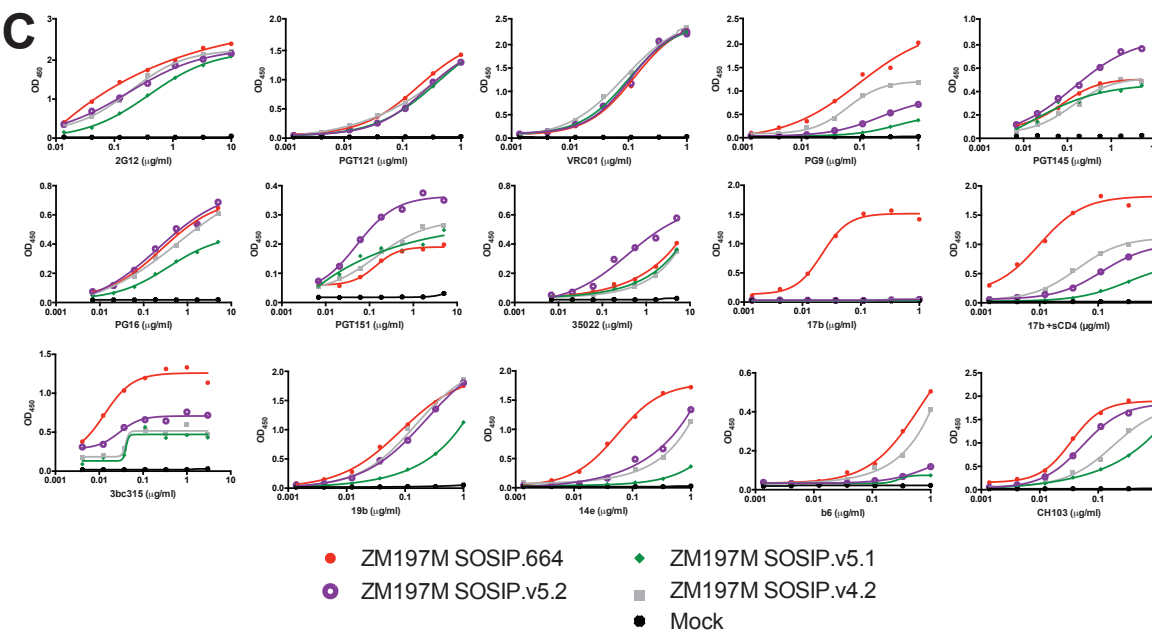

Data S1

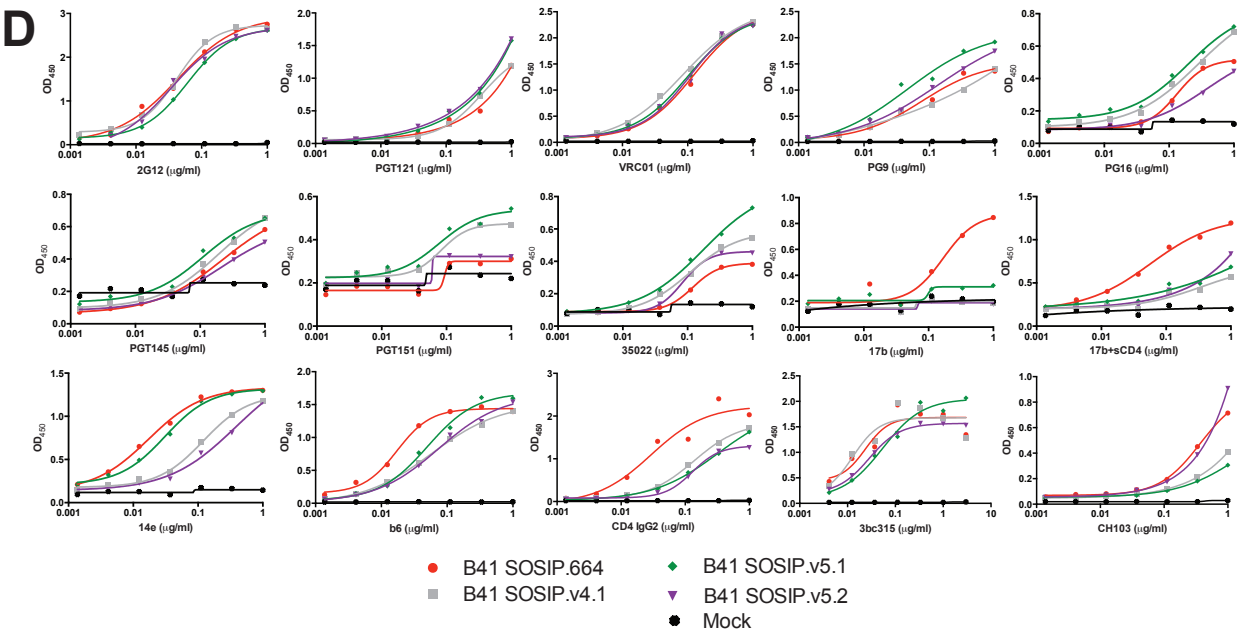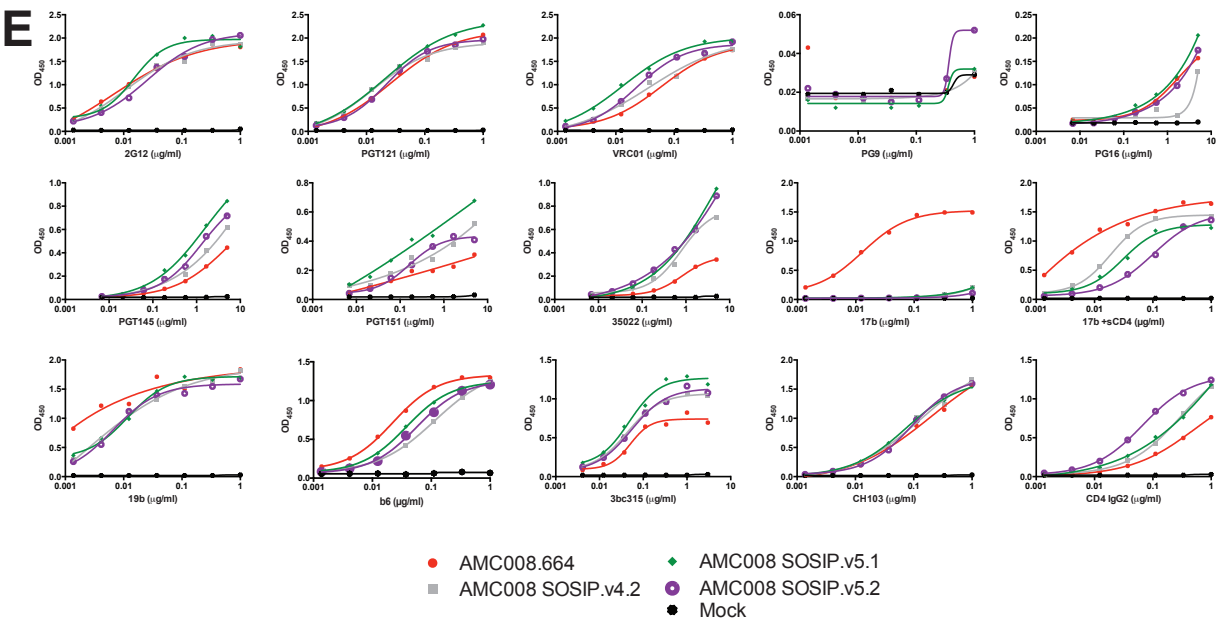

Supplement: Table S4. Midpoint Neutralization Titers of Sera from Rabbits Immunized with Stabilized SOSIP Trimers, Related to Figures 2 and 3 — The table shows midpoint neutralization titers of rabbit sera tested against the parental BG505.T332N (wild-type), and pseudoviruses in which specific PNGSs were mutated, and midpoint neutralization titers of rabbit sera from prebleeds and week 22 sera against autologous and heterologous pseudoviruses. [file mmc2.pdf]
