# Supplementary material for: Improving the Immunogenicity of Native-like HIV-1 Envelope Trimers by Hyperstabilization
Source: Cell Rep. 2017 Aug 23;20(8):1805–17. doi: 10.1016/j.celrep.2017.07.077 (PMC5590011; doi:10.1016/j.celrep.2017.07.077)
Supplement: Document S2. Article plus Supplemental Information [file mmc4.pdf]

Cell Reports

Article

Improving the Immunogenicity of Native-like HIV-1 Envelope Trimers by Hyperstabilization

Graphical Abstract

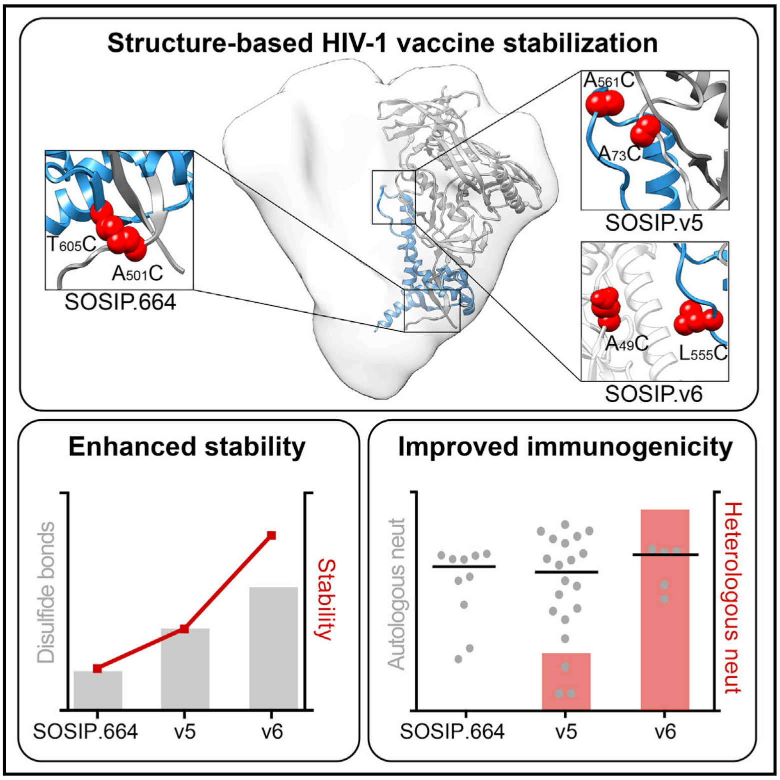

Authors

Alba Torrents de la Peña,
Jean-Philippe Julien,
Steven W. de Taeye, ..., Andrew B. Ward,
Ian A. Wilson, Rogier W. Sanders

Correspondence

rws2002@med.cornell.edu

In Brief

Native-like HIV-1 envelope trimers are a platform for efforts to induce broadly neutralizing antibodies. Torrents de la Peña et al. design HIV-1 envelope trimers with enhanced stability and reduced flexibility. These modified trimers improve the induction of neutralizing antibodies and provide new opportunities toward elicitation of broadly neutralizing antibodies.

Highlights

- The stability of HIV-1 envelope trimers can be increased by structure-based design
- Design improvements are applicable to envelope trimers from different HIV-1 isolates
- Immunization of stabilized HIV-1 envelope trimers is improved

Accession Numbers

5WDU

Torrents de la Peña et al., 2017, Cell Reports *20*, 1805–1817
August 22, 2017 © 2018 The Authors.
http://dx.doi.org/10.1016/j.celrep.2017.07.077

CellPress

OPEN ACCESS
CellPress

Improving the Immunogenicity of Native-like HIV-1 Envelope Trimers by Hyperstabilization

Alba Torrents de la Peña,[1] Jean-Philippe Julien,[2,10,11] Steven W. de Taeye,[1] Fernando Garces,[2,12] Miklos Guttman,[3] Gabriel Ozorowski,[2] Laura K. Pritchard,[4] Anna-Janina Behrens,[4] Eden P. Go,[5] Judith A. Burger,[1] Edith E. Schermer,[1] Kwinten Sliepen,[1] Thomas J. Ketas,[6] Pavel Pugach,[6] Anila Yasmeen,[6] Christopher A. Cottrell,[2] Jonathan L. Torres,[2] Charlotte D. Vavourakis,[7] Marit J. van Gils,[1] Celia LaBranche,[8] David C. Montefiori,[8] Heather Desaire,[5] Max Crispin,[4] Per Johan Klasse,[6] Kelly K. Lee,[3] John P. Moore,[6] Andrew B. Ward,[2] Ian A. Wilson,[2,9] and Rogier W. Sanders[1,6,13,*]
[1]Department of Medical Microbiology, Academic Medical Center, University of Amsterdam, Amsterdam 1105 AZ, the Netherlands
[2]Department of Integrative Structural and Computational Biology, Scripps CHAVI-ID, IAVI Neutralizing Antibody Center and Collaboration for AIDS Vaccine Discovery (CAVD), The Scripps Research Institute, La Jolla, CA 92037, USA
[3]Department of Medicinal Chemistry, University of Washington, Seattle, WA 98195, USA
[4]Oxford Glycobiology Institute, Department of Biochemistry, University of Oxford, Oxford OX1 3QU, UK
[5]Department of Chemistry, University of Kansas, Lawrence, KS 66047, USA
[6]Department of Microbiology and Immunology, Weill Medical College of Cornell University, New York, NY 10021, USA
[7]Microbial Systems Ecology, Department of Freshwater and Marine Ecology, Institute for Biodiversity and Ecosystem Dynamics, University of Amsterdam, Amsterdam 1098 XH, the Netherlands
[8]Department of Surgery, Duke University Medical Center, Durham, NC 27710, USA
[9]The Skaggs Institute for Chemical Biology, The Scripps Research Institute, La Jolla, CA 92037, USA
[10]Present address: Program in Molecular Medicine, The Hospital for Sick Children Research Institute, Toronto, ON M5G 0A4, Canada
[11]Present address: Departments of Biochemistry and Immunology, University of Toronto, Toronto, ON M5S 1A8, Canada
[12]Present address: Department of Therapeutic Discovery, Amgen, Inc., Thousand Oaks, CA 92320, USA
[13]Lead Contact
*Correspondence: rws2002@med.cornell.edu
http://dx.doi.org/10.1016/j.celrep.2017.07.077

SUMMARY

The production of native-like recombinant versions of the HIV-1 envelope glycoprotein (Env) trimer requires overcoming the natural flexibility and instability of the complex. The engineered BG505 SOSIP.664 trimer mimics the structure and antigenicity of native Env. Here, we describe how the introduction of new disulfide bonds between the glycoprotein (gp)120 and gp41 subunits of SOSIP trimers of the BG505 and other genotypes improves their stability and antigenicity, reduces their conformational flexibility, and helps maintain them in the unliganded conformation. The resulting next-generation SOSIP.v5 trimers induce strong autologous tier-2 neutralizing antibody (NAb) responses in rabbits. In addition, the BG505 SOSIP.v6 trimers induced weak heterologous NAb responses against a subset of tier-2 viruses that were not elicited by the prototype BG505 SOSIP.664. These stabilization methods can be applied to trimers from multiple genotypes as components of multivalent vaccines aimed at inducing broadly NAbs (bNAbs).

INTRODUCTION

Despite many attempts, no experimental vaccine has induced strongly protective immunity against HIV-1 infection. One approach to this problem is the generation of an envelope glycoprotein (Env)-based vaccine that induces broadly neutralizing antibodies (bNAbs) (van Gils and Sanders, 2013). A major obstacle to creating such a vaccine is the instability of the Env trimer, which for many years hindered the generation of recombinant, soluble proteins that adequately mimicked the functional Env trimer on virions.

We have described a soluble, recombinant Env trimer, BG505 SOSIP.664, that is stabilized by a disulfide bond between glycoprotein (gp)120 and gp41 and an Ile-to-Pro substitution at position 559 in gp41 (Binley et al., 2000; Sanders et al., 2002, 2013). Several BG505 trimer structures, determined by X-ray crystallography and cryoelectron microscopy (cryo-EM), have provided new insights into the architecture and function of HIV-1 Env (Garces et al., 2015; Julien et al., 2013a; Kwon et al., 2015; Lee et al., 2016; Lyumkis et al., 2013; Pancera et al., 2014; Sanders and Moore, 2014; Scharf et al., 2015; Stewart-Jones et al., 2016). Negative-stain electron microscopy (EM) and cryo-EM studies show that it closely resembles the native, membrane-associated trimer at the structural level (Lee et al., 2016; Lyumkis et al., 2013; Sanders et al., 2013). The BG505 SOSIP.664 trimer, and others of the same design based on different genotypes, displays the epitopes for most bNAbs and few non-NAbs (Derking et al., 2015; Huang et al., 2014; Julien et al., 2013b; Sanders et al., 2013). In immunogenicity studies in rabbits, the BG505, AMC008, B41, CZA97, and DU422 SOSIP.664 trimers induced NAbs against the corresponding autologous viruses (Cheng et al., 2015; Klasse et al., 2016; Sanders et al., 2015; de Taeye et al., 2015).

Although SOSIP.664 trimers are stable enough to produce and purify, we hypothesized that their performance as immunogens could be improved by reducing their conformational flexibility and the consequent exposure of immunodominant, potentially distractive non-NAb epitopes (de Taeye et al., 2015).

This is an open access article under the CC BY license (http://creativecommons.org/licenses/by/4.0/).

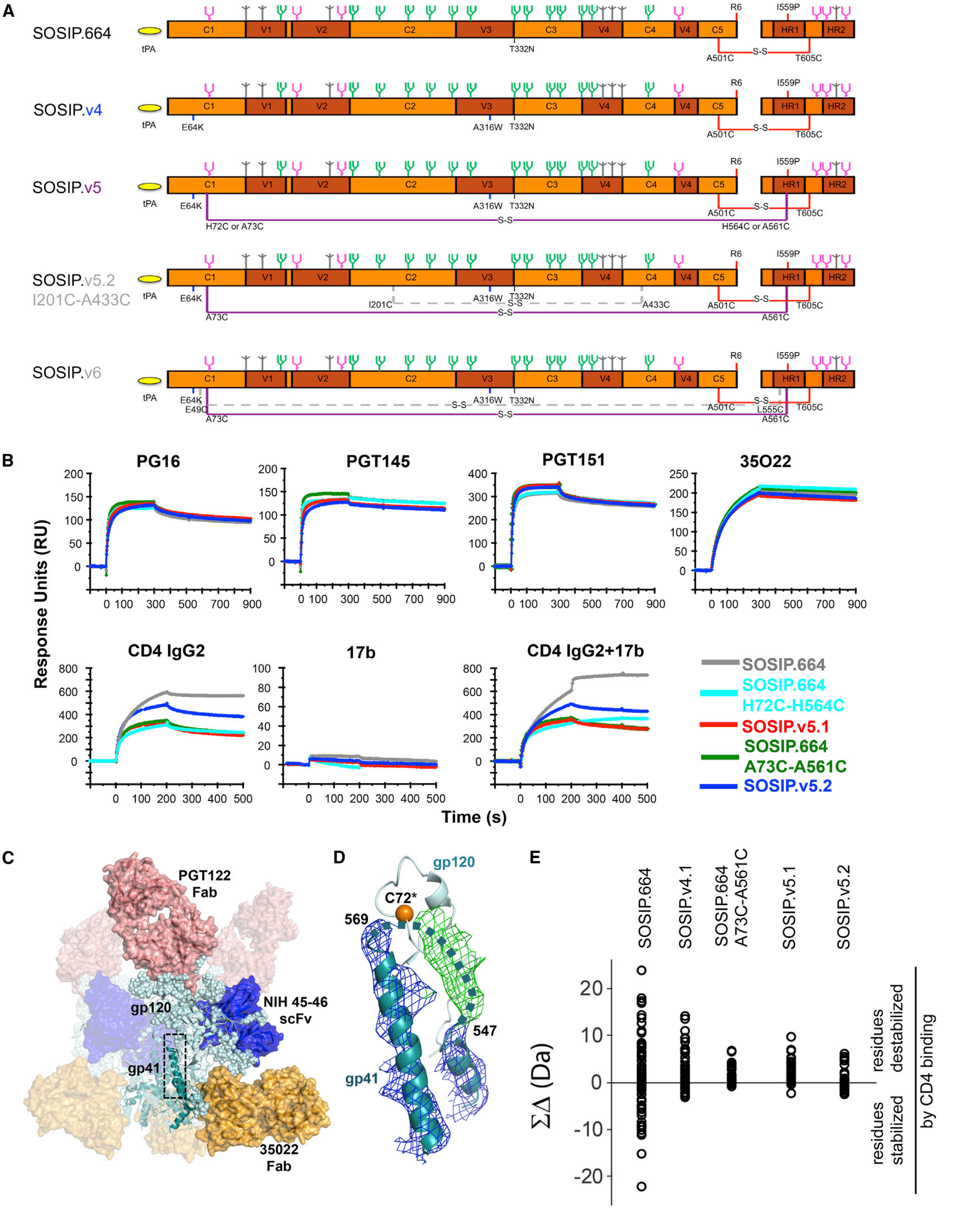

(legend on next page)

The increasingly high-resolution structures of SOSIP trimers greatly facilitate the design of stabilization strategies. BG505 SOSIP.664 trimers have already been further stabilized by adding an intra-gp120 disulfide bond linking residues 201 and 433, which fixes the bridging sheet in its ground state and thereby reduces the exposure of non-NAb epitopes (Guenaga et al., 2015; Kwon et al., 2015). Combining two different substitutions in the gp120 subunit, E64K or H66R plus A316W, defined as SOSIP.v4, increases the stability of trimers of various genotypes, reduces the exposure of non-NAb CD4i and V3 epitopes (i.e., 17b, 19b, and 14e), and decreases the induction of V3-directed non-NAbs in immunized rabbits (de Taeye et al., 2015). Forming a complex with the quaternary-structure-dependent bNAb, PGT145, can also improve trimer stability (Cheng et al., 2015). Moreover, by comparing the BG505 sequence with other sequences that form stable trimers poorly, several substitutions were identified that increase the stability of JR-FL and 16055 SOSIP.664 trimers (Guenaga et al., 2015). Steichen et al. (2016) used mammalian cell surface display to design stabilized trimers that showed an improvement in trimer expression and reduction of V3 exposure. A considerable improvement in yield was achieved by computationally redesigning the N-terminal residues of heptad region 1 (HR1) of Env SOSIP trimers (Kong et al., 2016). Finally, we have also introduced a disulfide bond between gp120 residue-49 of one protomer and gp41 residue-555 of a second protomer to increase thermostability of the trimer. Strong precedents underpin this approach as disulfide bonds play a well-established role in protein stability (Camacho and Thirumalai, 1995; Creighton, 1988). For example, disulfide bonds can be up to 17-fold more abundant in proteins from thermophilic archea and bacteria, compared to mesophiles, and the number of disulfides correlates with their maximum growth temperature (Liszka et al., 2012). Furthermore, disulfide bonds have been used to stabilize the immunogens from respiratory syncytial virus (RSV) and influenza virus (Lee et al., 2016; McLellan et al., 2013).

Here, we describe the structure-guided introduction of additional disulfide bonds between gp120 and gp41 that further stabilize the resulting SOSIP.v5 trimers in their unliganded, closed state. Furthermore, we have combined these intra-protomer bonds with recently described inter-protomer bonds to create hyperstable SOSIP.v6 trimers. When BG505 SOSIP.v6 trimers were tested as immunogens in rabbits, they induced strong autologous responses, as well as weak, but consistent, NAbs against a subset of heterologous tier-2 viruses.

RESULTS

Addition of a Second Disulfide Bond between gp120 and gp41

To reduce the flexibility and increase the stability of BG505 SOSIP.664 trimers, we designed, screened, and produced variants containing an additional engineered intra-protomer disulfide bond; i.e., between the gp120 and gp41 subunits. Since the disulfide bond between residue 501 of gp120 and residue 605 of gp41 (i.e., the SOS bond), used to create SOSIP trimers, is located near the base of the trimer (Binley et al., 2000; Garces et al., 2015; Pancera et al., 2014), we sought more central locations for a second intra-protomer bond. Initially, new disulfide bonds were evaluated in the absence of the SOS bond, and a few promising candidates were subsequently chosen to make double disulfide-bond variants (Figures S1 and S2). Based on favorable biochemical and antigenic properties, we selected two such variants, designated SOSIP.664 H72C-H564C and SOSIP.664 A73C-A561C, for further analyses (Figures S1 and S2). To provide additional stability, we also introduced two point substitutions: A316W to improve hydrophobic packing of V3 residues and prevent sporadic, unwanted V3 exposure from its hidden location below V1V2 and E64K to impede the occasional spontaneous and reversible sampling of the CD4-bound conformation (Figure 1A; de Taeye et al., 2015). SOSIP.664 trimers containing the E64K and A316W substitutions and either an H72C-H564C or A73C-A561C disulfide bond are referred to as SOSIP.v5.1 or SOSIP.v5.2 variants, respectively (Figure 1A; see also Table S1).

Figure 1. Design, Antigenicity, Structure, and Conformational Dynamics of BG505 SOSIP.v5 Trimers Containing Novel Disulfide Bonds between gp120 and gp41

(A) Linear schematic of the BG505 SOSIP.664, SOSIP.v4, SOSIP.v5, SOSIP.v5.2 I201C-A433C, and SOSIP.v6 constructs. Modifications that create the SOSIP.664 construct are indicated in red (Sanders et al., 2013). The E64K and A316W substitutions added to make the SOSIP.v4 construct are colored blue (de Taeye et al., 2015). The engineered disulfide bonds in SOSIP.v5 are shown in purple. There are two variants of the SOSIP.v5 construct: the SOSIP.v5.1 has a disulfide bond between H72C and H564C and the SOSIP.v5.2 has a disulfide bond between A73C and A561C. The I201C-A433C disulfide bond, previously described by Kwon et al. (2015), was introduced into the SOSIP.v5.2 background. The E49C-L555C disulfide bond was introduced into the SOSIP.v5.2 construct. The resulting constructs are designated SOSIP.v5.2 I201C-A433C and SOSIP.v6, respectively.

(B) SPR analysis of the binding of bNAbs PG16, PGT145, PGT151, and 35O22 to quaternary epitopes (upper panel), and of CD4-IgG2 and non-NAb 17b CD4i (±prior addition of CD4-IgG2) (lower panel), to the indicated BG505 trimer variants.

(C) Crystal structure of the quaternary complex of BG505 SOSIP.664 H72C-H564C trimer (cyan) in complex with PGT122 Fab (pink), 35O22 Fab (orange), and NIH45-46 scFv (deep blue). The three different antibody Fab or scFv fragments are represented as colored surfaces, and the trimer N-linked glycans as cyan spheres. One of the three protomers is highlighted for clarity, and the gp41 central HR1 helix location proximal to the engineered H72C-H564C disulfide is marked with a rectangle.

(D) Detail of the interaction between gp41 HR1 and gp120. A 2Fo-Fc composite omit map contoured at 1.0σ around gp41 HR1 (blue mesh) reveals ordered electron density (green mesh) for gp41 $HR1_N$ residues 547–569 (dotted lines), implying that the H72C-H564C disulfide bond helps stabilize this region of the trimer in a ground-state prefusion conformation. The figure was rendered using PyMOL.

(E) Differences in hydrogen/deuterium (H/D) exchange rates between the unliganded and CD4-bound forms of the indicated SOSIP variants. The difference between the two states is the difference obtained by subtracting the deuterons exchanged in the unliganded condition from the deuterons exchanged in the CD4-bound forms. While positive values indicate that the residues were destabilized in the presence of 2D-sCD4, negative values indicate stabilization. The net difference in H/D exchange as a sum of all time points is plotted for each observable peptide. Only differences that were outside the error range were included in the summation process. The same set of peptides was used for each trimer construct. The individual exchange plots are shown in Data S1A.

Biochemical and Biophysical Properties of Stabilized BG505 SOSIP.v5 Trimers

The two variants of BG505 SOSIP.v5 trimers were expressed in 293F cells and purified via PGT145-affinity chromatography, as previously described (de Taeye et al., 2015). Non-reducing SDS-PAGE analysis showed that the SOSIP.v5 proteins migrated more slowly than their SOSIP.664 counterparts (Figures S3A and S3B), consistent with a decrease in SDS uptake when a protein becomes more compact. We used tandem mass spectrometry (MS/MS) to confirm that, in addition to the ten canonical disulfide bonds and the SOS bond, the new bond was also formed in both SOSIP.v5 variants (Table S5).

PGT145-purified, native-like BG505 SOSIP.664 trimers have a more open configuration on average compared to those purified by 2G12 columns, typically ~30% versus >95% closed (de Taeye et al., 2015). The addition of the H72C-H564C or A73C-A561C disulfides further increases to 85% closed for SOSIP.v5.1 and 90% for SOSIP.v5.2 trimers (Table 1 and Figure S3C). Dynamic light scattering (DLS) studies support the conclusion that both SOSIP.v5 trimers are more compact (see Table S2 and Figure S3D for these and other biophysical analyses, including small angle X-ray scattering [SAXS]).

We then assessed BG505 trimer thermostability. The introduction of either disulfide bond in the SOSIP.v4.1 context again increased trimer thermostability with T_m values of 75.0°C and 75.3°C for SOSIP.v5.1 and SOSIP.v5.2, respectively, representing increases of 4.3°C and 4.6°C over SOSIP.v4.1 and 7.4°C and 7.7°C over SOSIP.664 (Table 1; Figure S3E).

The glycosylation profiles of the SOSIP.664 H72C-H564C, SOSIP.664 A73C-A561C, and both SOSIP.v5 trimers were all similar to those of SOSIP.664 and SOSIP.v4.1, with oligomannose glycoforms dominating (61%–64%) and, in particular, $Man_8GlcNAc_2$ and $Man_9GlcNAc_2$ glycans (Table 1; Figure S3F). The high density of unprocessed oligomannose glycans on gp120 is a hallmark of native-like Env trimers (Pritchard et al., 2015a, 2015b).

Antigenicity of Stabilized BG505 SOSIP.v5 Trimers

We used a panel of bNAbs to assess the antigenicity of stabilized BG505 trimer variants by ELISA. All of the tested bNAbs bound comparably to the SOSIP.664, SOSIP.v4, and SOSIP.v5 trimers, implying that the changes did not compromise antigenicity (Table 2; Data S1A). Surface plasmon resonance (SPR) studies confirmed that the quaternary-structure-dependent epitopes at the trimer apex (PG16 and PGT145) and at the gp120/gp41 interface (35O22 and PGT151) were fully preserved on both versions of SOSIP.v5 trimers (Figure 1B). SPR analysis showed that the association of CD4-IgG2 was similar between SOSIP.664 and SOSIP.v5, but the dissociation from both SOSIP.v5 variants was markedly faster (Figure 1B), consistent with previous measurements with SOSIP.v4 trimers (de Taeye et al., 2015). These data are also consistent with a report in which stabilization of BG505 SOSIP.664 with an intra-gp120 disulfide bond (DS-SOSIP.664) was shown to increase soluble CD4 (sCD4) dissociation (Kwon et al., 2015).

BG505 SOSIP.664 trimers minimally, but detectably, bind 17b, a non-NAb against a CD4-inducible (CD4i) epitope, by ELISA (Sanders et al., 2013). Under the same conditions, neither SOSIP.v5 trimer bound detectably to 17b when CD4-immunoglobulin G (IgG2) was absent, and the extent of CD4i-epitope induction by CD4-IgG2 was less than with SOSIP.664 and SOSIP.v4.1 in ELISA (Table 2; Data S1B). SPR analysis confirmed and extended these results (Figure 1B). Moreover, non-NAb b6 that targets the CD4 binding site (CD4bs) bound less well to the SOSIP.v5 trimers than to SOSIP.664 and SOSIP.v4.1 (Table 2; Data S1B). Some of the reduction in non-NAb epitope exposure in SOSIP.v5 is attributable to the A316W substitution that is present in SOSIP.v4 trimers (de Taeye et al., 2015). However, the new disulfide bonds in the SOSIP.v5 trimers confer additional benefits (Table 2; Data S1B).

Taken together, the antigenicity studies show that the SOSIP.v5 trimers preserve the desired bNAb reactivity profiles of their SOSIP.664 and SOSIP.v4.1 precursors.

X-Ray Structure of a Stabilized BG505 SOSIP Trimer

To assess the impact of an extra disulfide bond on the trimer structure, we expressed the BG505 SOSIP.664 H72C-H564C trimer in N-acetyl-glucosaminyltransferase I (GnTI)-deficient HEK293S cells and purified it by 2G12-affinity chromatography followed by size exclusion chromatography (SEC). Its crystal structure was determined in complex with bNAbs PGT122 Fab, 35O22 Fab, and NIH45-46 scFv (Figure 1C). Although the crystals only diffracted to 7 Å resolution, the availability of higher-resolution structures of other BG505 SOSIP trimers allowed a clear interpretation of the lower-resolution electron density maps, as exemplified by good refinement statistics (Table S3). The overall architecture of the trimer is indeed preserved upon addition of the 72C-564C inter-subunit disulfide bond (Figure 1C). In addition, bNAbs against three distinct sites of vulnerability (PGT122, N332; 35O22, gp41/gp120 interface; and NIH45-46, CD4bs) interacted with their epitopes in a similar manner to the SOSIP.664 trimer (Figure 1C). Residues 547–569 in the gp41 $HR1_N$ region near the 72C substitution in gp120 have defined electron density similar to that in higher-resolution BG505 SOSIP.664 trimer structures (Garces et al., 2015; PDB: 5CEZ) filtered at the same resolution (Figure 1D), indicating that this region is well ordered and native like. Thus, the added H72C-H564C disulfide bond preserves the native-like trimer structure.

Dynamics of BG505 SOSIP.v5 Trimers

We compared the SOSIP constructs by hydrogen-deuterium exchange (HD-X) to explore the impact of the new trimer-stabilizing changes on protein dynamics and conformational flexibility. The resulting exchange profiles for the BG505 SOSIP.664 A73C-A561C, SOSIP.v5.1 and SOSIP.v5.2 trimers were overall consistent with the SOSIP.664 precursor (Figure S4). Only a few minor changes were observed, which are proximal to the newly introduced disulfide linkages. We used the same method to compare CD4-induced conformational changes. Addition of sCD4 to the SOSIP.664 trimers resulted in less protection in the V2 and V3 loops, but more protection of the CD4bs, gp120 layers 1–3, and gp41 HR1, in agreement with previous studies (Figures 1E and S4) (Guttman et al., 2014). The changes in the various stabilized mutants were greatly diminished or completely abolished (Figures 1E and S4). The SOSIP.v5.2 variant responded least to

Table 1. Biophysical Characterization of Stabilized SOSIP Trimers from BG505, AMC008, B41, and ZM197M Isolates

New Substitutions Added to SOSIP.664	SOSIP Version[a]	Yield (mg/L) (≈)	Morphology (NS-EM): Native-like (%) (>)	Morphology (NS-EM): Closed Native-like (%)	Thermostability (DSC)[b]: T_m (°C)	Thermostability (DSC)[b]: ΔT_m (°C)	Glycan Composition (HILIC-UPLC)[c]: Man_8	Glycan Composition (HILIC-UPLC)[c]: Man_9	Glycan Composition (HILIC-UPLC)[c]: Oligo-mannose
BG505									
–	SOSIP.664	2.0	98	35	67.6	–	18	23	64
H72C-H564C		2.0	98	70	71.9	4.3	21	20	63
A73C-A561C		2.0	98	70	72.5	4.9	ND	ND	ND
E64K A316W	SOSIP.v4.1[d]	2.0	98	70	70.7	3.1	21	23	64
E64K A316W H72C-H564C	SOSIP.v5.1	2.0	98	85	75.0	7.4	21	20	68
E64K A316W A73C-A561C	SOSIP.v5.2	2.0	98	90	75.3	7.7	21	23	64
I201C-A433C[e]	DS-SOSIP	1.0	98	65	74.5	8.9	ND	ND	ND
E64K A316W A73C-A561C I201C-A433C		1.5	98	85	80.7	13.1	23	19	61
E49C-L555C[f]		0.2	ND	ND	75.2	7.6	ND	ND	ND
E64K A316W A73C-A561C E49C-L555C	SOSIP.v6	0.8	98	50	78.7/92.2[g]	11.2/24.6[g]	25	25	69
AMC008									
–	SOSIP.664	2.0	98	15	60.2	–	14	34	67
I535M L543N H66R A316W	SOSIP.v4.2[d]	2.0	98	85	64.5	4.5	12	39	69
I535M L543N H66R A316W H72C-H564C	SOSIP.v5.1	1.5	98	100	68.3	8.1	13	33	65
I535M L543N H66R A316W A73C-A561C	SOSIP.v5.2	1.5	98	100	68.5	8.3	14	30	65
B41									
–	SOSIP.664	2.0	98	45	58.6	–	15	29	63
L543N E64K A316W	SOSIP.v4.1[d]	2.0	98	55	60.7	2.1	14	32	64
L543N E64K A316W H72C-H564C	SOSIP.v5.1	0,2	98	95	63.0	4.3	ND	ND	ND
L543N E64K A316W A73C-A561C	SOSIP.v5.2	1.5	98	70	64.7	6.1	17	25	64
ZM197M									
–	SOSIP.664	0.3	95	15	62.2	–	22	26	70
V535M H66R T316W	SOSIP.v4.2[d]	0.3	90	30	62.6	0.4	21	26	69
V535M H66R T316W H72C-H564C	SOSIP.v5.1	1.0	98	100	69.5	7.3	ND	ND	ND
V535M H66R T316W A73C-A561C	SOSIP.v5.2	1.0	98	100	69.2	7.0	ND	ND	ND

The yields of PGT145-purified trimers are listed, together with the percentages that have a native-like conformation and also in the completely closed form, as determined by negative-stain EM. The 2D class averages were derived using trimers without a C-terminal tag and are shown in Figure S5C. ND, not determined.

[a]An overview of the modifications made to the stabilized trimer variants is shown in Table S1.

[b]The T_m values for each construct were obtained by DSC using a two-state model (Figure S5D). The T_m values are based on two-state model fitting. T_m values for His-tagged trimers (BG505) were consistently ~0.9°C –1.0°C higher than the values for the same trimers without a tag. T_m values obtained with D7324-tagged trimers (AMC008, B41, and ZM197M) were up to 0.3°C higher than those without a tag.

[c]Percentages of Man_8, Man_9, and total oligomannose glycans are given for each trimer (Figure S5E). The percentage of $Man_8GlcNAc_2$ and $Man_9GlcNAc_2$ glycans, as well as the total percentage of oligomannose glycans are provided for each His/D7324-tagged trimer (Figure S5E).

[d]NS-EM and glycan composition data for SOSIP v4.1 and SOSIP v4.2 trimers were previously reported in de Taeye et al. (2015).

[e]Data for this trimer variant were previously described in Kwon et al. (2015) and Guenaga et al. (2015). Experiments were performed with newly purified trimers.

[f]SOSIP.664 trimer variant was described in Garces et al. (2015). Experiments were performed with newly purified trimers.

[g]The two values represent the peaks of the two independent unfolding events observed by DSC (see Figure S3E for the raw data).

Table 2. Antigenic Characterization of Stabilized Trimers from BG505, AMC008, B41, and ZM197M Isolates

		Broadly Neutralizing Antibodies									Non-neutralizing Antibodies					
		V1V2 Apex			V3-Glycan	OD Glycan	CD4bs	gp120-gp41 Interface			CD4i		V3		CD4bs	CD4bs
New Substitutions Added to SOSIP.664	SOSIP Version[a]	PG9	PG16	PGT145	PGT121	2G12	VRC01	PGT151	35022	3BC315	17b	17b+sCD4	14e	19b	B6	CD4 IgG2
BG505																
–	SOSIP.664	0.079	0.054	0.020	0.044	0.014	0.022	0.016	0.026	0.130	0.304	0.077	0.039	0.013	0.143	0.020
H72C-H564C		90	98	76	88	129	118	87	73	89	0	56	72	88	55	80
A73C-A561C		95	158	84	115	119	105	136	93	111	0	43	71	83	59	61
E64K A316W	SOSIP.v4.1[b]	91	93	77	101	104	113	107	71	106	0	15	2	0	13	54
E64K A316W H72C-H564C	SOSIP.v5.1	69	81	94	72	103	106	118	77	95	0	8	2	4	9	35
E64K A316W A73C-A561C	SOSIP.v5.2	77	94	86	79	122	116	131	75	93	0	8	2	5	7	55
E64K A316W A73C-A561C I201C-A433C		76	95	231	88	85	87	135	130	102	0	0	2	9	10	61
E64K A316W A73C-A561C E49C-L555C	SOSIP.v6	112	103	135	58	123	78	130	85	99	0	9	2	6	5	68
AMC008																
–	SOSIP.664	ND	1.761	1.300	0.026	0.010	0.061	0.162	0.832	0.050	0.13	0.001	0.002	ND	0.017	0.534
I535M L543N H66R A316W	SOSIP.v4.2[a]	ND	62	125	114	95	102	115	162	116	0	1	6	ND	30	103
I535M L543N H66R A316W H72C-H564C	SOSIP.v5.1	ND	128	134	127	69	102	118	171	107	0	1	12	ND	34	63
I535M L543N H66R A316W A73C-A561C	SOSIP.v5.2	ND	127	130	119	40	118	185	231	95	0	0	10	ND	23	96
B41																
–	SOSIP.664	0.077	0.094	0.151	0.053	0.043	0.126	0.152	1.665	0.024	0.839	0.001	0.007	0.003	0.017	0.029
L543N E64K A316W	SOSIP.v4.1[a]	97	104	92	108	102	126	121	115	58	0	1	8	14	20	21
L543N E64K A316W H72C-H564C	SOSIP.v5.1	127	73	59	104	70	125	232	135	44	0	2	1	18	34	9
L543N E64K A316W A73C-A561C	SOSIP.v5.2	113	418	86	116	125	116	221	159	84	0	5	28	16	23	23
ZM197M																
–	SOSIP.664	0.263	0.487	2.310	0.192	0.126	0.034	0.142	0.013	0.022	0.10	0.055	0.064	0.637	0.295	0.138
V535M H66R T316W	SOSIP.v4.2[a]	73	93	113	88	95	108	95	85	28	0	42	14	55	59	92
V535M H66R T316W H72C-H564C	SOSIP.v5.1	16	91	352	84	96	125	234	92	28	0	3	12	17	9	13
V535M H66R T316W A73C-A561C	SOSIP.v5.2	30	121	342	87	102	116	267	425	45	0	5	19	36	10	26

Binding of bNAbs and non-NAbs was determined using a Ni-NTA (BG505) or a D7324- (AMC008, B41, and ZM197M) capture ELISA. Half-maximal binding concentrations (half maximal effective concentration [EC_{50}], in μg/mL) are shown for SOSIP.664 trimers (underlined). Antibody binding to the various stabilized trimers is expressed as percentage of the binding to SOSIP.664 (EC_{50} is defined as 100%). The values are representative of at least two independent experiments. The ELISA curves from one representative experiment are shown in Data 1B–1E. ND, not determined.

[a]An overview of the modifications made to the stabilized trimer variants is shown in Table S1.

[b]The SOSIP v4.1 and SOSIP v4.2 trimer variants were described in de Taeye et al. (2015).

sCD4, in that the CD4-induced exposure of V2 and V3 was now entirely abrogated (Figure S4). Thus, the extra inter-subunit disulfide bond and the E64K and A316W substitutions are synergistic in preventing the opening of the apex of the SOSIP.v5.2 trimers when sCD4 is present. The data strongly suggest that the BG505 SOSIP.v5.2 trimers are effectively trapped in the closed, ground state.

Stabilizing SOSIP Trimers from Clades B and C

To assess the generality of the stabilization method, we introduced the H72C-H564C or A73C-A561C disulfide bonds into B41 (clade B) SOSIP.v4.1, AMC008 (clade B), and ZM197M (clade C) SOSIP.v4.2 constructs (Julien et al., 2015; Pugach et al., 2015; de Taeye et al., 2015). The resulting variants were purified and analyzed by SDS-PAGE and blue native (BN)-PAGE analysis. While AMC008 SOSIP.v5 and B41 SOSIP.v5 yielded similar trimer amounts to comparably produced SOSIP.664 and SOSIP.v4 counterparts, the yields for ZM197M were considerably improved (1.0 mg/L for SOSIP.v5.1 and v5.2 versus ~0.3 mg/L for SOSIP.664 and SOSIP.v4) (Table 1). All of the variously purified SOSIP.v5 trimers were cleaved efficiently (Figures S5A and S5B) and migrated more slowly on non-reducing SDS-PAGE gels (Figure S5A). Negative-stain EM confirmed that the PGT145-purified SOSIP.v5 trimers were again invariably native like and also more likely to be in the closed conformation (Table 1; Figure S5C). As for BG505, oligomannose glycoforms dominated (63%–70%, in particular $Man_8GlcNAc_2$ and $Man_9GlcNAc_2$, Table 1; Figure S5E). The additional disulfide bond improved the thermostability of each trimer construct. For AMC008, the T_m increased from 60.2°C (SOSIP.664) to 68.3°C–68.5°C (SOSIP.v5.1 and v5.2; Table 1; Figure S5D). Increases in T_m of between 4°C and 7°C compared to SOSIP.664 were also observed for the various B41 and ZM197M SOSIP.v5.1 and SOSIP.v5.2 trimers (Table 1; Figure S5D). Overall, we conclude that introducing a second inter-subunit disulfide bond confers additional stability on SOSIP.v5 trimers of four different isolates from three different clades.

Antigenic Properties of B41, AMC008, and ZM197M SOSIP.v5 Trimers

In general, the antigenicity properties of the SOSIP.v5.1 and v5.2 trimers of all three genotypes were again comparable to the corresponding SOSIP.v4 and/or SOSIP.664 trimers, bNAb binding was retained, while non-NAb reactivity with V3 and CD4i epitopes was reduced (Table 2; Data S1C–S1E). CD4-IgG2 binding to B41 and ZM197M SOSIP.v5 variants was reduced compared to SOSIP.v4 (Table 2). An unexpected observation was that introduction of an additional disulfide bond into the ZM197M SOSIP.v5 construct improved binding of the quaternary-structure-dependent bNAb PGT145 and PGT151, compared to SOSIP.664 and SOSIP.v4 trimers (Table 2; Data S1C). We noticed that in contrast to the quaternary-specific PGT145 bNAb, the quaternary preferring PG9 showed a slightly decreased binding for ZM197M SOSIP.v5 compared to SOSIP.664 (Table 2; Data S1C). The introduction of an additional disulfide bond also had a noticeable effect for B41, for which PGT151 binding was negligible for SOSIP.664 trimers, but strong for both SOSIP.v5 versions (Table 2; Data S1D). We can conclude that SOSIP.v5 exhibits a similar or somewhat better antigenic profile compared to SOSIP.664 and SOSIP.v4.1.

Comparison and Combination with Other Trimer Stabilization Approaches

Two other SOSIP trimer stabilization strategies are outlined in Figure 1A. First, introducing a disulfide bond between residues 201 and 433 (substitutions I201C and A433C) in the gp120 bridging sheet has been reported to increase the thermostability and reduce the conformational flexibility of these BG505 trimers (Kwon et al., 2015). Second, a disulfide bond between gp120 and gp41 of different protomers (substitutions E49C and L555C) also makes BG505 trimers more thermostable (Garces et al., 2015). BG505 SOSIP trimer variants containing either of these disulfide bonds have a reduced exposure of CD4i non-NAb epitopes (Garces et al., 2015; Kwon et al., 2015).

We introduced the above disulfide bonds into BG505 SOSIP.664 or SOSIP.v5.2 constructs and determined the yields, antigenic profiles, and thermal stability of the resulting trimers. The SOSIP.v6 construct contains the E49C-L555C substitutions on the SOSIP.v5.2 background, while the "SOSIP.v5.2 I201C-A433C" construct includes the 201C-433C disulfide bond in the SOSIP.v5.2 background (Figure 1A). The yields of PGT145-purified SOSIP.v5.2 I201C-A433C and SOSIP.v6 trimers (~1.5 and ~0.8 mg/L, respectively) were lower than for SOSIP.664 and SOSIP.v5.2 (~2.0 mg/L) (Table 1). Non-reducing SDS-PAGE analysis showed that SOSIP.v6 proteins migrated as a trimer, which is consistent with the formation of inter-protomer disulfide bonds (Table S3A). However, two subpopulations were visible on the gels. Based on a comparison with the migration patterns of various control proteins, we propose that the slower migrating variant, ~60% of the total, contains all three engineered disulfide bonds (501C-605C, 73C-561C, and 49C-555C), while the faster migrating species lacks the 73C-561C bond whose formation may be restricted by the presence of the 49C-555C bond in a subpopulation of trimers (Figure S3A).

The antigenic profile of these more stable trimers, SOSIP.v5.2 I201C-A433C and SOSIP.v6, are very similar to their less stable counterparts. The quaternary antibodies PGT151 and PGT145 bound slightly more strongly to the BG505 SOSIP.v5.2 I201C-A433C and SOSIP.v6 trimers than to their precursors, while the binding of non-NAbs to V3 and CD4i epitopes remained very low (Table 2; Data S1B). Both new trimers resembled their precursors in containing mostly oligomannose glycoforms (Table 1; Figure S3F).

The new disulfide bonds further increased BG505 trimer thermostability. Adding the 201C-433C bond to the SOSIP.v5.2 construct increased the T_m from 75.3°C to 80.7°C. The SOSIP.v6 trimer was particularly thermostable, the T_m of the majority subpopulation was 92.2°C, while a smaller unfolding event was detected at 78.8°C, which we propose reflects the minority of trimers in which the 73C-561C linkage is not formed (Table 1; Figure S3E).

Immunogenicity of Stabilized BG505 SOSIP Trimers in Rabbits

We compared the immunogenicity of various prototypic and stabilized BG505 SOSIP trimers in rabbits using a previously

described protocol with 22 μg of trimer per dose and measuring the antibody responses 2 weeks after the third immunization (Figure 2A) (Sanders et al., 2015; de Taeye et al., 2015).

All the BG505 trimer-immunized animals had high and comparable titers of binding antibodies to the corresponding trimers, as measured in ELISA (Figure 2C). The sera from the SOSIP.664 trimer recipients consistently neutralized the autologous tier-2 BG505.T332N virus, which is consistent with our earlier reports (Figure 2D) (Sanders et al., 2015; de Taeye et al., 2015). The corresponding gp120 monomer was almost entirely ineffective in this regard, with only one of five animals responding weakly (Figure 2D). The inferiority of BG505 gp120 at inducing autologous BG505.T332N NAbs was consistent with a previous report, although the difference between the gp120 recipients and the trimer-immunized animals was much more pronounced in the current experiment (Sanders et al., 2015; Figure 2D). The autologous NAb responses elicited by the different trimers were generally comparable (median half maximal inhibitory concentration [IC_{50}]; SOSIP.664, 4432; SOSIP.v4.1, 4503; SOSIP.v5.1 + SOSIP.v5.2 pooled, 3457; and SOSIP.v6, 7,798), except for the SOSIP.v5.2 I201C-A433C hyperstabilized trimer (median IC_{50}, 226; $p = 0.0159$ compared to SOSIP.664) (Figure 2D). Thus, with the possible exception of the I201C-A433C change to the SOSIP.v5.2 construct, the modifications used to create new, more stable trimers do not impair the induction of the autologous BG505.T332N NAb response.

One of the goals of trimer stabilization projects is to reduce the antigenicity and immunogenicity of epitopes for non-NAbs and tier-1 NAbs, the latter being dominated by V3-directed antibodies, and thereby focus the immune response on more productive targets (Kwon et al., 2015; de Taeye et al., 2015). Compared to the BG505 SOSIP.664 trimers and, more so, the gp120 monomers, NAb titers against the tier-1A SF162 virus were reduced by 3-fold for the combined SOSIP.v5.1 and SOSIP.v5.2 groups ($p = 0.0175$ versus SOSIP.664; not statistically significant versus SOSIP.v4.1) and were also lower for SOSIP.v5.2 201C-433C (not statistically significant versus SOSIP.664). In contrast, the SF162 NAb titers, as well as V3 binding antibody titers (data not shown), were almost 7-fold higher in the SOSIP.v6 group compared to the combined SOSIP.664, SOSIP.v4.1, and SOSIP.v5 groups ($p = 0.0031$; Figure 2E).

Sera from the various BG505 trimer-immunized rabbits were generally weakly and sporadically active against a panel of heterologous tier-2 viruses (Table S4A). While the clade A virus 92RW020, the clade B virus SHIV162P3, and the clade C TV1.21 virus were poorly or not neutralized by SOSIP.664-, SOSIP.v4.1-, or SOSIP.v5-immunized animals (median IC_{50} values for the combined SOSIP.664, SOSIP.v4.1, and SOSIP.v5 groups of 25, 20, and 28, respectively), they were more consistently neutralized by sera from SOSIP.v6 recipients (IC_{50} values of 56, 45, and 97). The differences in the ability to neutralize 92RW020, SHIV162P3, and TV1.21 between the BG505 SOSIP.v6 recipient animals and the animals that were immunized with earlier BG505 SOSIP versions were statistically significant ($p = 0.0071$, $p = 0.0021$, and $p = 0.0207$ for the three viruses, respectively). In contrast, prebleed sera did not neutralize the autologous BG505.T332N pseudovirus, nor the heterologous 92RW020 or SHIV162P3 pseudoviruses (Table S4C). For each individual rabbit, we analyzed the number of heterologous tier-2 viruses that were neutralized with IC_{50} values >40. Neutralization at this titer level was only sporadic for the SOSIP.664, SOSIP.v4.1, or SOSIP.v5 groups; the majority of the sera were inactive against all heterologous Tier-2 viruses. In contrast, sera from the SOSIP.v6 group neutralized one (one animal), two (one animal), three (one animal), or four (two animals) of the sixteen viruses in the test panel at a titer >40 (Figure 2I and Table S4A). When the SOSIP.v6 group was compared with the SOSIP.664, SOSIP.v4.1, SOSIP.v5.1 + SOSIP.v5.2, and SOSIP.v5.2 I201C-A433C groups, the number of heterologous Tier-2 viruses neutralized with IC_{50} values >40 was significantly higher ($n = 14$ for SOSIP.v6 versus $n = 0$, 4, 4, and 6 for SOSIP.664, SOSIP.v4.1, SOSIP.v5.1 + SOSIP.v5.2, and SOSIP.v5.2 I201C-A433C, respectively; Kruskal-Wallis $p < 0.001$ in each case) (Figure 2I).

Immunogenicity of Stabilized ZM197M SOSIP Trimers in Rabbits

In a similar study, we tested the ZM197M SOSIP.664, SOSIP.v4.2, and SOSIP.v5.2 trimers in rabbits (Figures 2J–2O). The autologous trimer binding antibody titers were again comparable among the groups, but ~2- to 5-fold lower than those induced by BG505 trimers (Figure 2J). The autologous NAb response to the ZM197M trimers, in general, was also markedly weaker and less consistent than seen with their BG505 counterparts (cf. Figure 2J with Figure 2C and Figure 2K with Figure 2D). The ZM197M SOSIP.v5.2 trimer was clearly the most immunogenic for the autologous NAb response (5/5 responders, with a median IC_{50} of 114, compared to 3/10 responders for the combined SOSIP.664 and SOSIP.v4.2 groups; $p < 0.0001$ by χ^2 test; Figure 2K). The three ZM197M trimers induced SF162 tier-1A NAb titers to comparable extents (Figure 2L).

As seen in the BG505 study, sera against the various ZM197M trimers only sporadically neutralized heterologous tier-2 viruses (Table S4A). Overall, the most frequent NAb responses to 92RW020, SHIV162P3, and TV1.21 were induced by the ZM197M SOSIP.v5.2 variant ($p = 0.007$, non-significant and $p = 0.0137$ for 92RW020, SHIV162P3, and TV1.21, respectively compared to SOSIP.664 and SOSIP.v4.2 combined; Figures 2N–2P). Three of the five SOSIP.v5.2 sera neutralized three heterologous tier-2 viruses at a titer >40, while none of the SOSIP.664 or SOSIP.v4.2 sera did so (Kruskal-Wallis $p < 0.001$; Figure 2P). Assessed across the entire heterologous tier-2 panel, the ZM197M SOSIP.v5.2 sera neutralized significantly more viruses (IC_{50} values > 40) compared to the SOSIP.664 or SOSIP.v4.2 groups ($n = 9$ versus 0 or 0, respectively, Fischer-Freeman $p < 0.0001$; Figure 2P).

In conclusion, the hyperstable BG505 SOSIP.v6 trimers induced significantly broader tier-2 NAb responses than the SOSIP.664, SOSIP.v4, and SOSIP.v5 variants. For the ZM197M genotype, the SOSIP.v5.2 construct was the most broadly immunogenic for tier-2 NAbs compared to its SOSIP.664 and SOSIP.v4.2 counterparts.

Properties of the Autologous Tier-2 NAb Specificities

To investigate what epitopes were targeted by the autologous BG505.T332N NAbs induced by, in particular, BG505 SOSIP.v6 trimers in rabbits, we first performed neutralization depletion

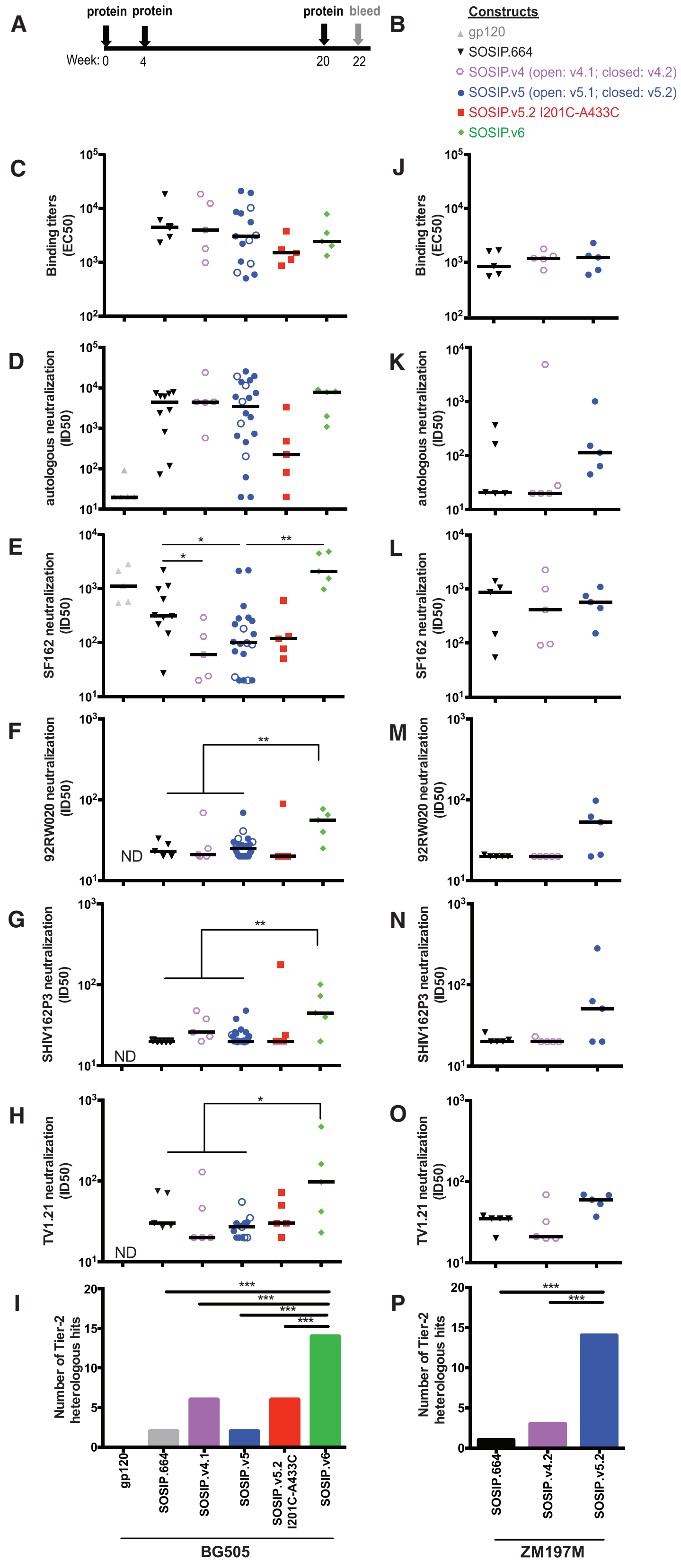

Figure 2. Immunogenicity of BG505 and ZM197M SOSIP.v5 Trimers in Rabbits

(A) Schematic representation of immunization schedule.

(B) Color coding for the immunogens tested.

(C–I) Shows data for BG505-based immunogens.

(J –P) Shows data for ZM197M.

(C and J) Midpoint antibody binding titers (ED_{50}) as measured by D7324-capture ELISA for the trimer variants indicated on the x axis at the bottom of the figure, and by the color-coding scheme outlined in (B).

(D–H and J–O) Neutralization of HIV-1 viruses in the TZM-bl assay by sera from animals immunized with BG505 or ZM197M trimer variants. The plots show ID_{50} values, the serum dilution at which infectivity is inhibited by 50%.

(D–H and K–O) Autologous viruses (D and K); SF162 heterologous tier-1A (E and L); 92RW020 heterologous tier-2 virus (F and M); SHIV162P3 heterologous tier-2 virus (G and N); and TV1.21 heterologous tier-2 virus (H and O).

(I and P) The cumulative numbers of heterologous viruses neutralized with an ID_{50} >40 by the five sera from each group are shown. In the particular case of the BG505 SOSIP.v5 group, where 15 animals were analyzed (SOSIP.v5.1: five animals and SOSIP.v5.2: ten animals), the number of heterologous hits was divided by three. Statistical tests were performed using Mann-Whitney t test for (E)–(H) and Kruskal-Wallis with Dunn's post-test for (I) and (P). Note that Kruskal-Wallis tests were performed on the ID_{50} values. The horizontal bars represent medians. Neutralization titers with SOSIP.v6 sera against 92RW020, SHIVP3, and TV1.21 (plotted in F–H and M–O) were obtained from two or three independent experiments performed in duplicate. The average values are plotted, and the data from the replicate experiments can be found in Table S4C.

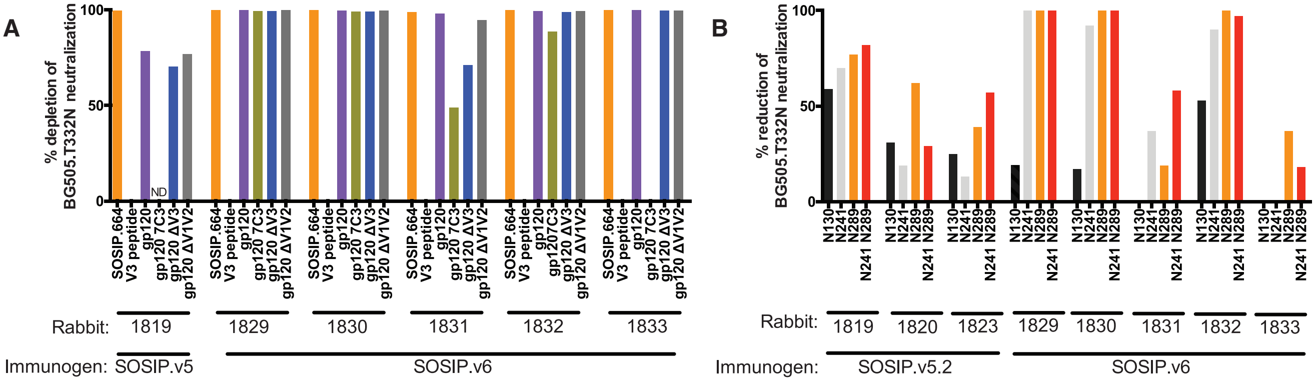

C

	Rabbit ID	MG505.A2	MG505.A2 K241S	MG505.H3	TV1.21	TV1.21 N160A	TV1.21 N332A	TV1.21 K169E	TV1.21 N276A
	**1819**	<50	2655	<50	<20	<20	<20	<20	<20
**SOSIP.v5.2**	**1820**	<50	301	<50	28	68	120	<20	27
	**1823**	<50	97	<50	30	39	57	<20	30
	**1829**	<50	1991	<50	39	60	75	21	49
	**1830**	53	1939	54	76	107	381	21	86
**SOSIP.v6**	**1831**	878	6947	76	114	212	454	<20	78
	**1832**	75	4366	<50	337	799	1698	<20	227
	**1833**	<50	<50	<50	22	39	64	<20	<20

Figure 3. Properties of the Autologous NAb Specificities

(A) Neutralization depletion assays with sera from one BG505 SOSIP.v5.2 recipient and all five BG505 SOSIP.v6-immunized animals using the autologous BG505.T332N virus and a BG505 V3 peptide, as well as a set of different BG505 proteins. Neutralization of BG505.T332N from each individual sera was set at 100%, and the percent depletion was calculated when the V3 peptide or the different BG505 proteins were added in the assay. The percentage shown is the mean of two different experiments.

(B) Neutralization assays with BG505.T332N PNGS mutants. The ID_{50} of the individual sera with the parental BG505.T332N virus was set at 100%, and the percent reduction in ID_{50} when the respective PNGSs are introduced is shown. See also Table S4B.

(C) Neutralization of maternal MG505 viruses and heterologous TV1.21 mutant viruses. The ID_{50}s are shown. Strong neutralization ($ID_{50} > 1,000$) is shown in red; $1,000 > ID_{50} > 100$ is shown in orange; $100 > ID_{50} > 50$ is shown in yellow, and no neutralization ($ID_{50} < 50$) is shown in white.

experiments using a BG505 V3 peptide and a set of BG505 proteins: SOSIP.664 gp140, gp120, gp120-ΔV1V2, gp120-ΔV3, and gp120-7C3 (Sanders et al., 2015). All proteins contained the D368R substitution to prevent neutralization by CD4 binding and interference in neutralization assays. The gp120-7C3 contains seven substitutions in residues 354–363 in the C3 domain that were under selective pressure in the BG505-infected infant (Goo et al., 2014; Sanders et al., 2015). One BG505 SOSIP.v5.2 recipient animal was also included in the analyses.

The autologous NAb response in all SOSIP.v6 recipients and the one SOSIP.v5.2 recipient rabbit was completely depleted when the sera were preincubated with BG505 SOSIP.664 D368R trimer, confirming that the neutralization was trimer directed (Figure 3A). gp120, gp120-ΔV1V2, and gp120-ΔV3 were also efficient at depleting the BG505.T332N neutralization, indicating that most of the autologous NAb response was directed to gp120-associated epitope(s) that did not involve V1V2 or V3. Rabbit 1831 was an exception. In this animal, gp120-ΔV3 was considerably less efficient at depleting BG505.T332N neutralization compared to full-length gp120, suggesting that the V3 domain was involved in the (presentation of) the epitope for the autologous NAb response in this animal. The gp120-7C3 protein was unable to deplete the BG505.T332N NAb activity from the sera of rabbit 1833 and poorly effective in depleting the BG505.T332N NAb activity from animal 1831, suggesting that residues 354–363, in the C3 domain, are involved in the epitope(s) for the autologous NAb activity in these animals. BG505.T332N neutralization of all sera was unaffected by addition of the BG505 V3 peptide, confirming that autologous NAb response was not directed against simple linear epitopes in the V3 domain that are associated with tier-1A virus neutralization (Figure 3A; Sanders et al., 2015).

Next, we tested neutralization of BG505.T332N mutants, specifically focusing on virus mutants in which holes in the BG505 trimer glycan shield that are immunogenic in rabbits were filled in by restoring the respective predicted N-linked glycan sites (PNGSs) (Klasse et al., 2016; McCoy et al., 2016). We previously found that these holes, especially a large glycan hole centered around positions 241 and 289, are frequent targets of autologous BG505.T332N NAb responses induced by BG505 SOSIP trimers (Klasse et al., 2016).

Introduction of PNGSs at positions 241 and 289 almost completely abolished BG505.T332N neutralization by sera from three out of five SOSIP.v6 recipients, as well as one SOSIP.v5.2 recipient (animals 1819, 1829, 1830, and 1832), showing that the NAb activity in these animals was dominated by Ab specificities targeting the 241/289 hole (Figure 3B; Table S4B). The NAb activity in four other rabbits tested, two receiving SOSIP.v5.2 and two receiving SOSIP.v6 (animals 1831 and 1833), were much less affected by the PNGSs knockin mutations, showing that the NAb response in those rabbits was only

partly directed to the 241/289 glycan hole. The introduction of the PNGSs at position 130 generally had a much smaller effect on the NAb sensitivity compared to the introduction of PNGSs at positions 241 and 289. The largest effects were observed for animals 1819 (SOSIP.v5.2) and 1832 (SOSIP.v6), in which cases the NAb activity was reduced by ~50%.

The finding that introduction of the PNGSs at 241 and 289 only had a partial effect on the NAb activity in animals 1831 and 1833 is consistent with the observation that the gp120-7C3 protein did not deplete the NAb activity from the sera of these two animals. Thus, while the NAb specificities of three SOSIP.v6 recipient animals appear to be directed to the 241/289 glycan hole, those in the two other animals appear to be predominantly targeting an epitope that is affected by residues 354–363.

We also tested the maternal virus clones MG505.A2 and MG505.H3. While MG505.A2 has 14 amino-acid differences in gp160 compared to BG505.T332N, only one involves a change in a PNGSs: N332T. MG505.H3 contains 24 amino-acid differences compared to BG505.T332N, including the presence of a PNGS at 241 and the lack of PNGSs at positions 190, 234, 332, 363, and 411.

The sera of three rabbits immunized with BG505 SOSIP.v6 neutralized MG505.A2, but none immunized with BG505 SOSIP.v5.2 did. While MG505.A2 has a hole at position 241, the amino acid at that position is different compared to BG505.T332N (K for MG505.A2 and S for BG505.T332N). To mimic the BG505.T332N virus, a K241S substitution was introduced into MG505.A2. The sera from all the rabbits, except one animal, became substantially more sensitive to this virus, suggesting that the NAb specificities in these sera were directed toward this glycan hole, but were also dependent on the composition of amino acid 241. One exception was animal 1833, which did not neutralize MG505.A2 or its K241S variant, in agreement with the observation that the response in this animal did not target the 241/289 hole. Furthermore, although the serum from animal 1831 potently neutralized the MG505.A2 K241S virus, it also neutralized the parental MG505.A2 virus effectively, providing further evidence that NAb specificities other than those directed to the 241/289 hole were present in this animal. Finally, when sera were tested against the MG505.H3 virus, only two rabbits (1830 and 1831, both recipients of BG505 SOSIP.v6) neutralized this virus and then at low titer (Figure 3C), suggesting that any or all of the 24 amino-acid changes in the MG505.H3 virus cause resistance to the NAb specificities present in the SOSIP.v6-immunized rabbit sera.

Finally, to obtain information on the heterologous NAb responses, we tested some of the sera against a set of TV1.21 pseudovirus mutants. The results showed that knocking out the N160 or N332 glycan enhanced TV1.21 neutralization by SOSIP.v6 sera, suggesting that these glycans shield the heterologous neutralization epitope(s) targeted by the SOSIP.v6 recipient animals. Conversely, the K169E mutation ablated neutralization, suggesting that K169 is part of the neutralization epitope, or that the K169E mutation destroys the target epitope by indirect means. Removal of the N276 glycan had no effect on the sensitivity to the SOSIP.v6 sera (Figure 3C; Table S4C). Because of the poor magnitude of the other heterologous NAb titers, we were not able to map them.

DISCUSSION

We describe the creation of native-like SOSIP trimers that are stabilized by the introduction of additional disulfide bonds. The most stable variant, BG505 SOSIP.v6, has its major T_m at 92.2°C and a minor T_m at 78.8°C, representing an increase of 24.5°C and 11.1°C over the prototypic SOSIP.664 design. The most stable trimers contain two new disulfide bonds per gp120-gp41 protomer, in addition to SOS. The 30 intra-molecular disulfide bonds naturally present in the trimer (ten per protomer) are located within the inner or outer domains of gp120 or, in one case, the immunodominant loop of gp41. In contrast, the engineered bonds are all inter-domain.

The inter-subunit disulfide bond linking gp120 residue-501 to gp41 residue-605 (the "SOS bond") was positioned without the availability of structural information on the gp120-gp41 interface (Binley et al., 2000). The cryo-EM structure of the native, membrane-associated trimer (Lee et al., 2016) confirms the accuracy with which this bond was placed in the SOSIP trimer. The newly introduced gp41 cysteine residues that successfully form inter-subunit disulfide bonds are located between the $\alpha6$ and $\alpha7$ segments (i.e., $HR1_N$) that were not well resolved in the initial BG505 SOSIP.664 trimer structures (Julien et al., 2013a; Lyumkis et al., 2013; Pancera et al., 2014). More recent structures and HD-X experiments imply that this region in the SOSIP structures might be quite dynamic and, hence, capable of adopting multiple conformations (Garces et al., 2015; Guttman et al., 2014). The finding that cysteine residues at multiple positions in gp41 HR1 can pair with gp120 residues 72 or 73 is consistent with the flexibility of this region (Lee et al., 2016). However, the trimer structure predicted various other positions for cysteine substitutions that did not, in practice, lead to the efficient formation of new disulfide bonds. One explanation is that the presence of additional cysteine residues can sometimes interfere with the proper oxidative folding and disulfide bond isomerization in the endoplasmic reticulum, leading to the production of misfolded proteins that are subsequently degraded (Bulleid and van Lith, 2014).

While a previous report showed a correlation between trimer stability and the ability to induce autologous NAb responses (Feng et al., 2016), we did not observe such a correlation here. Thus, the BG505 SOSIP.664, SOSIP.v4.1, SOSIP.v5.1, SOSIP.v5.2, and SOSIP.v6 trimers, displaying a wide range of stabilities, were all equivalent in inducing BG505.T332N NAbs. However, the observation that the BG505 SOSIP.v6 trimer and the ZM197M SOSIP.v5 trimer were more efficient at inducing heterologous tier-2 NAb responses suggests that the relationship between trimer stability and the ability to induce NAbs is likely to be complex. Regardless, the BG505 SOSIP.v6 and ZM197M SOSIP.v5.2 trimers have encouraging immunogenicity properties in rabbits and constitute a baseline for further trimer design and delivery improvements. An additional virtue of a more stable vaccine immunogen is an increased shelf life and simplified storage capacity under real-word conditions (Karp et al., 2015).

From a broader perspective, the reduced conformational flexibility of stabilized trimers may help maintain them in the ground state for longer in vivo, thereby maximizing the presentation of

bNAb epitopes and increasing the probability of a successful encounter with susceptible but rare B cells. How these various factors intersect to drive the induction of higher titer autologous and heterologous tier-2 NAb titers is likely to be also influenced by the genotype of the trimer, as well as its design.

EXPERIMENTAL PROCEDURES

Trimer Expression and Purification

The constructs expressing BG505, B41, AMC008, and ZM1097M SOSIP.664 were transiently expressed in adherent 293T cells or suspension 293F cells in the presence of *furin* and purified using PGT145-affinity chromatography, as previously described (Julien et al., 2013a, 2015; Pugach et al., 2015; Sanders et al., 2013; de Taeye et al., 2015).

Antigenicity Assays

SPR analyses were performed with His-tagged trimers immobilized to CM5 chips, as previously described (Derking et al., 2015; Yasmeen et al., 2014). D7324-capture, nickel nitrilotriacetic acid (Ni-NTA)-capture, and thermostability ELISAs have been described elsewhere (Derking et al., 2015; Sanders et al., 2013; de Taeye et al., 2015).

Biophysical Techniques

Multiple biophysical techniques were used to analyze the properties of the Env trimers, including DLS, SAXS, differential scanning calorimetry (DSC), hydrophilic interaction chromatography-ultra-performance liquid chromatography (HILIC-UPLC), and HDX-MS. Images of Env trimers were generated by negative stain EM (NS-EM) following previously described procedures (Sanders et al., 2013). X-ray crystallography was performed with BG505 Env trimer together with different Fabs as previously described (Julien et al., 2013a).

Neutralization Assays

We used Env-pseudotyped or chimeric molecular clone viruses to perform neutralization assays at Duke University Medical Center, Academic Medical Center, and Weill Medical College of Cornell University (Sanders et al., 2013; Klasse et al., 2016).

Statistics

Different groups in rabbit immunizations were compared by two-tailed Mann-Whitney U tests. Kruskal-Wallis with Dunn's post-test was used when differences between the entire heterologous tier-2 panel were addressed.

Rabbit Immunizations

Rabbits were immunized with 22 μg of trimer and ISCOMATRIX at weeks 0, 4, and 20. Rabbit immunizations were performed under contract at Covance Research Products Inc. (Denver, PA, USA) under permits with approval numbers C0022-15, C0119-15, and C0120-15.

ACCESSION NUMBERS

The accession number for the trimer sequences reported in this paper is PDB: 5WDU.

SUPPLEMENTAL INFORMATION

Supplemental Information includes Supplemental Experimental Procedures, five figures, five tables, and a data file and can be found with this article online at http://dx.doi.org/10.1016/j.celrep.2017.07.077.

AUTHOR CONTRIBUTIONS

Conceptualization, A.T.d.l.P., J.-P.J., R.W.S., and I.A.W.; Methodology, A.T.d.l.P., J.-P.J., S.W.d.T., M.G., G.O., L.K.P., A.-J.B., E.P.G., J.A.B., E.E.S., K.S., T.J.K., P.P., A.Y., C.A.C., J.L.T., M.J.v.G., C.L., D.C.M., H.D., M.C., P.J.K., K.K.L., J.P.M., A.B.W., I.A.W., and R.W.S.; Writing - Original Draft, A.T.d.l.P., J.-P.J., J.P.M., A.B.W., I.A.W., and R.W.S.; Writing - Review & Editing, A.T.d.l.P., J.-P.J., S.W.d.T., F.G., M.G., G.O., T.J.K., P.P., C.D.V., D.C.M., H.D., M.C., P.J.K., K.K.L., J.P.M., A.B.W., I.A.W., and R.W.S.; and Funding Acquisition, A.B.W., J.P.M., I.A.W., and R.W.S.

ACKNOWLEDGMENTS

We thank James Robinson, Dennis Burton, John Mascola, Peter Kwong, Mark Connors, and William Olson for donating antibodies and reagents directly or through the AIDS reagents reference program; Malin Suurkuusk for incorporation of the independent non-two-state model in the NanoAnalyze software; Sal Butera and Dennis Burton for use of rabbit sera from the C0124-15 study; Hansi Dean, Wayne Koff, Joanne Stefano, and Beth Rasmussen for their contributions to rabbit studies C0022-15, C0045-15, C0119-15, and C0120-15; and Mitch Hartog, Luigi van Riel, Tim Rijkhold-Meesters, and Enzo Scutigliani for technical help. We thank Tsutomu Matsui and the support staff at the Stanford Synchrotron Radiation Lightsource (SSRL) for assistance with SAXS data collection. This work was supported by grants from NIH (P01 AI110657) and Scripps (CHAVI-ID UM1 AI100663), the International AIDS Vaccine Initiative (IAVI), an Aids fonds Netherlands grant (2012041), and a Canadian Institutes for Health Research grant (THA-118628). R.W.S. is a recipient of a Vidi Grant from the Netherlands Organization for Scientific Research (NWO) and a Starting Investigator grant from the European Research Council (ERC-StG-2011–280829-SHEV). The EM work was conducted at The Scripps Research Institute with support from Collaboration for AIDS Vaccine Discovery (CAVD) grants (OPP1084519 and OPP1115782). SSRL is supported by the U.S. Department of Energy, Office of Science and Office of Basic Energy Sciences under contract no. DE-AC02-76SF00515.

Received: March 21, 2017
Revised: June 20, 2017
Accepted: July 26, 2017
Published: August 22, 2017

REFERENCES

Binley, J.M., Sanders, R.W., Clas, B., Schuelke, N., Master, A., Guo, Y., Kajumo, F., Anselma, D.J., Maddon, P.J., Olson, W.C., et al. (2000). A recombinant human immunodeficiency virus type 1 envelope glycoprotein complex stabilized by an intermolecular disulfide bond between the gp120 and gp41 subunits is an antigenic mimic of the trimeric virion-associated structure. J. Virol. *74*, 627–643.

Bulleid, N.J., and van Lith, M. (2014). Redox regulation in the endoplasmic reticulum. Biochem. Soc. Trans. *42*, 905–908.

Camacho, C.J., and Thirumalai, D. (1995). Modeling the role of disulfide bonds in protein-folding: entropic barriers and pathways. Proteins *22*, 27–40.

Cheng, C., Pancera, M., Bossert, A., Schmidt, S.D., Chen, R., Chen, X., Druz, A., Narpala, S., Doria-Rose, N.A., McDermott, A.B., et al. (2015). Immunogenicity of a prefusion HIV-1-envelope trimer in complex with a quaternary-specific antibody. J. Virol. *90*, 2740–2755.

Creighton, T.E. (1988). Disulphide bonds and protein stability. BioEssays *8*, 57–63.

de Taeye, S.W., Ozorowski, G., Torrents de la Peña, A., Guttman, M., Julien, J.P., van den Kerkhof, T.L.G.M., Burger, J.A., Pritchard, L.K., Pugach, P., Yasmeen, A., et al. (2015). Immunogenicity of stabilized HIV-1 Envelope trimers with reduced exposure of non-neutralizing epitopes. Cell *163*, 1702–1715.

Derking, R., Ozorowski, G., Sliepen, K., Yasmeen, A., Cupo, A., Torres, J.L., Julien, J.P., Lee, J.H., van Montfort, T., de Taeye, S.W., et al. (2015). Comprehensive antigenic map of a cleaved soluble HIV-1 envelope trimer. PLoS Pathog. *11*, e1004767.

Feng, Y., Tran, K., Bale, S., Kumar, S., Guenaga, J., Wilson, R., de Val, N., Arendt, H., DeStefano, J., Ward, A.B., et al. (2016). Thermostability of well-ordered HIV spikes correlates with the elicitation of autologous tier 2 neutralizing antibodies. PLoS Pathog. *12*, e1005767.

Garces, F., Lee, J.H., de Val, N., Torrents de la Peña, A., Kong, L., Puchades, C., Hua, Y., Stanfield, R.L., Burton, D.R., Moore, J.P., et al. (2015). Affinity maturation of a potent family of HIV antibodies is primarily focused on accommodating or avoiding glycans. Immunity *43*, 1053–1063.

Goo, L., Chohan, V., Nduati, R., and Overbaugh, J. (2014). Early development of broadly neutralizing antibodies in HIV-1-infected infants. Nat. Med. *20*, 655–658.

Guenaga, J., de Val, N., Tran, K., Feng, Y., Satchwell, K., Ward, A.B., and Wyatt, R.T. (2015). Well-ordered trimeric HIV-1 subtype B and C soluble spike mimetics generated by negative selection display native-like properties. PLoS Pathog. *11*, e1004570.

Guttman, M., Garcia, N.K., Cupo, A., Matsui, T., Julien, J.P., Sanders, R.W., Wilson, I.A., Moore, J.P., and Lee, K.K. (2014). CD4-induced activation in a soluble HIV-1 Env trimer. Structure *22*, 974–984.

Huang, J., Kang, B.H., Pancera, M., Lee, J.H., Tong, T., Feng, Y., Georgiev, I.S., Chuang, G.Y., Druz, A., Doria-Rose, N.A., et al. (2014). Broad and potent HIV-1 neutralization by a human antibody that binds the gp41-120 interface. Nature *515*, 138.

Julien, J.P., Cupo, A., Sok, D., Stanfield, R.L., Lyumkis, D., Deller, M.C., Klasse, P.-J., Burton, D.R., Sanders, R.W., Moore, J.P., et al. (2013a). Crystal structure of a soluble cleaved HIV-1 envelope trimer. Science *342*, 1477–1483.

Julien, J.P., Sok, D., Khayat, R., Lee, J.H., Doores, K.J., Walker, L.M., Ramos, A., Diwanji, D.C., Pejchal, R., Cupo, A., et al. (2013b). Broadly neutralizing antibody PGT121 allosterically modulates CD4 binding via recognition of the HIV-1 gp120 V3 base and multiple surrounding glycans. PLoS Pathog. *9*, e1003342.

Julien, J.P., Lee, J.H., Ozorowski, G., Hua, Y., Torrents de la Peña, A., de Taeye, S.W., Nieusma, T., Cupo, A., Yasmeen, A., Golabek, M., et al. (2015). Design and structure of two HIV-1 clade C SOSIP.664 trimers that increase the arsenal of native-like Env immunogens. Proc. Natl. Acad. Sci. USA *112*, 1–6.

Karp, C.L., Lans, D., Esparza, J., Edson, E.B., Owen, K.E., Wilson, C.B., Heaton, P.M., Levine, O.S., and Rao, R. (2015). Evaluating the value proposition for improving vaccine thermostability to increase vaccine impact in low and middle-income countries. Vaccine *33*, 3471–3479.

Klasse, P.J., Labranche, C.C., Ketas, T.J., Ozorowski, G., Cupo, A., Pugach, P., Ringe, R.P., Golabek, M., van Gils, M.J., Guttman, M., et al. (2016). Sequential and simultaneous immunization of rabbits with HIV-1 envelope glycoprotein SOSIP.664 trimers from clades A, B and C. PLoS Pathog. *12*, e1005864.

Kong, L., He, L., de Val, N., Vora, N., Morris, C.D., Azadnia, P., Sok, D., Zhou, B., Burton, D.R., Ward, A.B., et al. (2016). Uncleaved prefusion-optimized gp140 trimers derived from analysis of HIV-1 envelope metastability. Nat. Commun. *7*, 1–15.

Kwon, Y.D., Pancera, M., Acharya, P., Georgiev, I.S., Crooks, E.T., Gorman, J., Joyce, M.G., Guttman, M., Ma, X., Narpala, S., et al. (2015). Crystal structure, conformational fixation and entry-related interactions of mature ligand-free HIV-1 Env. Nat. Struct. Mol. Biol. *22*, 522–531.

Lee, J.H., Ozorowski, G., and Ward, A.B. (2016). Cryo-EM structure of a native, fully glycosylated, cleaved HIV-1 envelope trimer. Science *351*, 1043–1048.

Liszka, M.J., Clark, M.E., Schneider, E., and Clark, D.S. (2012). Nature versus nurture: Developing enzymes that function under extreme conditions. Annu. Rev. Chem. Biomol. Eng. *3*, 77–102.

Lyumkis, D., Julien, J.P., de Val, N., Cupo, A., Potter, C.S., Klasse, P.-J., Burton, D.R., Sanders, R.W., Moore, J.P., Carragher, B., et al. (2013). Cryo-EM structure of a fully glycosylated soluble cleaved HIV-1 envelope trimer. Science *342*, 1484–1490.

McCoy, L.E., van Gils, M.J., Ozorowski, G., Messmer, T., Briney, B., Voss, J.E., Kulp, D.W., Macauley, M.S., Sok, D., Pauthner, M., et al. (2016). Holes in the glycan shield of the native HIV envelope are a target of trimer-elicited neutralizing antibodies. Cell Rep. *16*, 2327–2338.

McLellan, J.S., Chen, M., Leung, S., Graepel, K.W., Du, X., Yang, Y., Zhou, T., Baxa, U., Yasuda, E., Beaumont, T., et al. (2013). Structure of RSV fusion glycoprotein trimer bound to a prefusion-specific neutralizing antibody. Science *340*, 1113–1117.

Pancera, M., Zhou, T., Druz, A., Georgiev, I.S., Soto, C., Gorman, J., Huang, J., Acharya, P., Chuang, G.-Y., Ofek, G., et al. (2014). Structure and immune recognition of trimeric pre-fusion HIV-1 Env. Nature *514*, 455–461.

Pritchard, L.K., Vasiljevic, S., Ozorowski, G., Seabright, G.E., Cupo, A., Ringe, R., Kim, H.J., Sanders, R.W., Doores, K.J., Burton, D.R., et al. (2015a). Structural constraints determine the glycosylation of HIV-1 envelope trimers. Cell Rep. *11*, 1604–1613.

Pritchard, L.K., Harvey, D.J., Bonomelli, C., Crispin, M., and Doores, K.J. (2015b). Cell- and protein-directed glycosylation of native cleaved HIV-1 envelope. J. Virol. *89*, 8932–8944.

Pugach, P., Ozorowski, G., Cupo, A., Ringe, R., Yasmeen, A., de Val, N., Derking, R., Kim, H.J., Korzun, J., Golabek, M., et al. (2015). A native-like SOSIP.664 trimer based on an HIV-1 subtype B *env* gene. J. Virol. *89*, 3380–3395.

Sanders, R.W., and Moore, J.P. (2014). HIV: A stamp on the envelope. Nature *514*, 437–438.

Sanders, R.W., Vesanen, M., Schuelke, N., Schiffner, L., Kalyanaraman, R., Berkhout, B., Maddon, P.J., Olson, W.C., Lu, M., Moore, J.P., et al. (2002). Stabilization of the soluble, cleaved, trimeric form of the envelope glycoprotein complex of human immunodeficiency virus type 1. J. Virol. *76*, 8875–8889.

Sanders, R.W., Derking, R., Cupo, A., Julien, J.P., Yasmeen, A., de Val, N., Kim, H.J., Blattner, C., de la Peña, A.T., Korzun, J., et al. (2013). A next-generation cleaved, soluble HIV-1 Env trimer, BG505 SOSIP.664 gp140, expresses multiple epitopes for broadly neutralizing but not non-neutralizing antibodies. PLoS Pathog. *9*, e1003618.

Sanders, R.W., van Gils, M.J., Derking, R., Sok, D., Ketas, T.J., Burger, J.A., Ozorowski, G., Cupo, A., Simonich, C., Goo, L., et al. (2015). HIV-1 neutralizing antibodies induced by native-like envelope trimers. Science *349*, aac4223.

Scharf, L., Wang, H., Gao, H., Chen, S., McDowall, A.W., and Bjorkman, P.J. (2015). Broadly neutralizing antibody 8ANC195 recognizes closed and open states of HIV-1 Env. Cell *162*, 1379–1390.

Steichen, J.M., Kulp, D.W., Tokatlian, T., Escolano, A., Dosenovic, P., Stanfield, R.L., McCoy, L.E., Ozorowski, G., Hu, X., Kalyuzhniy, O., et al. (2016). HIV vaccine design to target germline precursors of glycan-dependent broadly neutralizing antibodies. Immunity *45*, 483–496.

Stewart-Jones, G.B., Soto, C., Lemmin, T., Chuang, G.Y., Druz, A., Kong, R., Thomas, P.V., Wagh, K., Zhou, T., Behrens, A.J., et al. (2016). Trimeric HIV-1-Env structures define glycan shields from clades A, B, and G. Cell *165*, 813–826.

van Gils, M.J., and Sanders, R.W. (2013). Broadly neutralizing antibodies against HIV-1: templates for a vaccine. Virology *435*, 46–56.

Yasmeen, A., Ringe, R., Derking, R., Cupo, A., Julien, J.P., Burton, D.R., Ward, A.B., Wilson, I.A., Sanders, R.W., Moore, J.P., and Klasse, J.P. (2014). Differential binding of neutralizing and non-neutralizing antibodies to native-like soluble HIV-1 Env trimers, uncleaved Env proteins, and monomeric subunits. Retrovirology *11*, 41.

Cell Reports, Volume *20*

Supplemental Information

Improving the Immunogenicity of Native-like HIV-1 Envelope Trimers by Hyperstabilization

Alba Torrents de la Peña, Jean-Philippe Julien, Steven W. de Taeye, Fernando Garces, Miklos Guttman, Gabriel Ozorowski, Laura K. Pritchard, Anna-Janina Behrens, Eden P. Go, Judith A. Burger, Edith E. Schermer, Kwinten Sliepen, Thomas J. Ketas, Pavel Pugach, Anila Yasmeen, Christopher A. Cottrell, Jonathan L. Torres, Charlotte D. Vavourakis, Marit J. van Gils, Celia LaBranche, David C. Montefiori, Heather Desaire, Max Crispin, Per Johan Klasse, Kelly K. Lee, John P. Moore, Andrew B. Ward, Ian A. Wilson, and Rogier W. Sanders

Figure S1

A

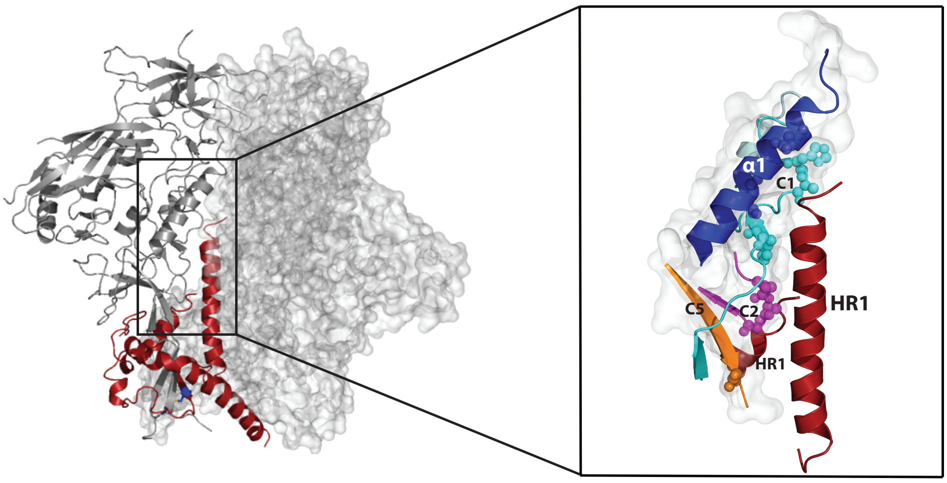

B

Location	Disulfide Bond	Trimerization	2G12	PGT145	PGT151	VRC01	PGT121	PG16	19b	F240	17b	17b+sCD4
C5 - DL	501-605	+++	+++	+++	+++	+++	+++	+++	+++	-	++	+++
C1-HR1	51-574	+	++	-	-							
	51-578	++	++	++	+							
	53-564	-	+	-	-							
	53-573	-	++	-	++							
	53-574	+++	++	+	+							
	72-554	++	++	++	+							
	72-555	+	++	++	++							
	72-564	++	+++	+++	+++	+++	+++	+++	+++	++	-	++
	73-558	++	+++	+++	+++	+++	+++	+++	+++	++	-	++
	73-561	++	+++	+++	+++	+++	+++	+++	+++	++	-	++
	73-567	++	+++	++	-							
	73-568	++	+++	+++	+++	+++	+++	+++	+++	++	-	++
α1-HR1	107-557	-	+	-	-							
	113-557	-	+	-	-							
	113-567	++	++	-	-							
loop A - HR1	220-568	+	+	+	++							
	221-568	+	++	+	+							
	221-571	-	+	+	-							
	221-578	+	++	-	-							
	221-579	+	++	+	++							
	221-582	+	++	++	++							
	222-571	-	+	-	-							
	222-579	-	++	-	-							
C5-HR1	492-574	-	++	-	-							
	492-585	++	++	+	++							

C

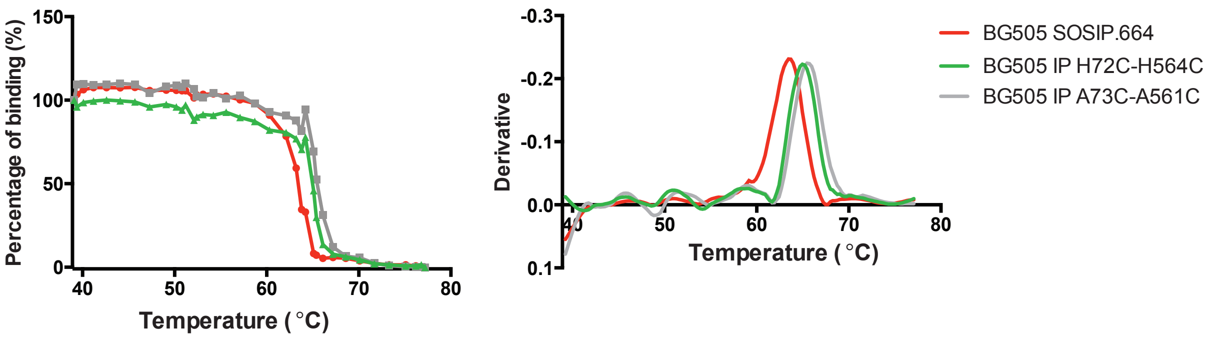

D

	T_m (°C)	ΔT_m (°C)
BG505 SOSIP.664	63.7	0.0
BG505 IP H72C-H564C	65.0	+1.3
BG505 IP A73C-A558C	64.5	+0.8
BG505 IP A73C-A561C	65.5	+2.0
BG505 IP A73C-A568C	64.0	+0.3
BG505 SOSIP.664 H72C-H564C	65.5	+1.8
BG505 SOSIP.664 A73C-A561C	66.0	+2.3

Figure S1. **Introducing a novel disulfide bond between gp120 and gp41.** Related to Fig. 1. (**A**) Five regions in gp120 and gp41 that are in reasonably close contact are shown in the crystal structure of BG505 SOSIP.664 (PDB: 4TVP). These regions involve residues in C1, C2 and C5 of gp120 and HR1 of gp41. The gp120 and gp41 subunits of a gp120-gp41 protomer are shown in grey and dark red, respectively. The SOS disulfide bond between residues 501 and 605 is colored blue. A close view of the gp120-gp41 interface is presented in the inset, where gp120 residues that likely contact gp41 are shown as blue spheres. (**B**) Screening of a panel of unpurified His-tagged BG505 SOSIP.664 proteins in which the original SOS disulfide bond (Cys501-Cys605) was replaced by two new cysteine residues in the regions highlighted in panel **A**, above. We used BG505 SOSIP.664 structures described in Julien et al., 2013b and Pancera et al., 2014 to design the structure-based disulfide bonds. BN-PAGE followed by western blotting was used to assess trimerization efficiency, which was reduced compared to SOSIP.664 for the majority of the mutants. The antigenic profile of the same trimer variants was determined by His-tag ELISA. If no disulfide bond is formed between the gp120 and gp41 subunits, they dissociate rapidly; the binding of the anti-gp120 MAb (2G12) to a construct captured via the His-tag on the gp41 subunit is therefore evidence for the successful formation of an intermolecular disulfide bond (Blattner et al., 2014; Ringe et al., 2013; Sanders et al., 2013). Several of the new constructs, particularly those with a cysteine residue introduced at position 72 or 73 in the loop between $\alpha\bar{1}$ and β0 together with one inserted within positions 554-568 in the loop between α6 and α7, were strongly reactive with 2G12. The implication is that an inter-subunit disulfide bond could form in these constructs. Two bNAbs that recognize different quaternary structure-dependent epitopes (PGT145: V1V2-apex; PGT151: gp120-gp41 interface) were used to assess the antigenic structure of the mutant trimers. Four mutants, H72C-H564C, A73C-A558C, A73C-A561C and A73C-L568C, bound to both PGT145 and PGT151 to similar extents to the original BG505 SOSIP.664 trimer; these cysteine pairs preserve the antigenic structure of the trimers appropriately. The antigenic profile of these four trimers was then assessed using a larger panel of bNAbs, which showed that the quaternary structure-dependent bNAbs 35022, 3BC315 and PG16 all bound efficiently each trimer. The spontaneous opening of the trimer variants was investigated using the CD4i non-NAb 17b, which did not bind to any of the four trimers in the absence of sCD4 in contrast with SOSIP.664, and did so only weakly when sCD4 was present. The anti-gp41 non-NAb F240 bound slightly more strongly to the four mutant trimers than to the original BG505 SOSIP.664 version, suggesting that the absence of the 501-605 disulfide exposes this epitope to some extent. This was confirmed using purified trimers (not shown). The F240 epitope in the immunodominant disulfide loop region of gp41 is known to overlap with residue 605, where the Cys residue involved in the SOS bond is located (Cavacini et al., 1998). Color code: red, no binding; orange, moderate binding; green, strong binding. Antibody binding is also scaled from no reactivity (-) to strong binding (+++). The data presented are based on 2 or 3 experiments. (**C**) The stability of unpurified His-tagged trimer mutants was evaluated using a thermal melting assay described elsewhere (de Taeye et al., 2015). The proteins were incubated for 1 h at varying temperatures in a graded PCR machine before 2G12 binding was assessed by Ni-NTA ELISA (right panel). The first derivative reveals the unfolding pattern (right panel). (**D**) The midpoints of thermal denaturation (T_m) for each trimer variant are based on the melting profiles in panel **C**. The T_m values for mutants containing the H72C-H564C or A73C-A561C disulfide bonds were increased by 1.3°C and 2.0°C, respectively, compared to SOSIP.664. The implication is that locating the disulfide bond at the core of the trimer, as opposed to the membrane proximal end in SOSIP.664, increases its stability. These results were conformed with purified trimers and DSC analysis (not shown). The T_m values for additional mutant trimers containing two disulfide proteins are also presented for reference (see Fig. S2).

Figure S2

A

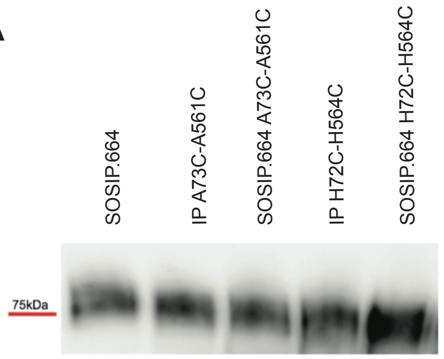

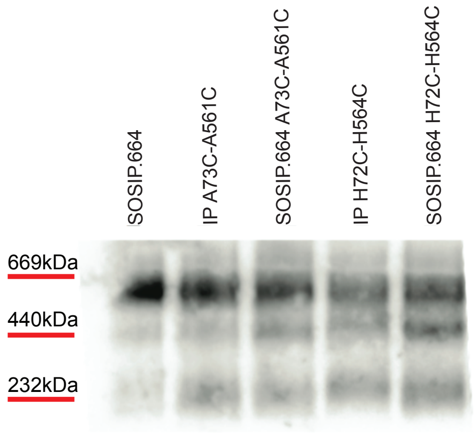

B

	2G12	PGT145	PGT151	VRC01	19b	17b	F240
BG505 SOSIP.664	+++	+++	+++	+++	++	++	-
BG505 IP H72C-H564C	+++	+++	+++	+++	++	-	++
BG505 IP A73C-A561C	+++	+++	+++	+++	++	-	++
BG505 SOSIP.664 H72C-H564C	+++	+++	+++	+++	++	-	-
BG505 SOSIP.664 A73C-A561C	+++	+++	+++	+++	++	-	-

C

BG505 SOSIP.664

Disulfide bond	2G12	PGT145	PGT151	17b	T_m (°C)
SOSIP.664	+++	+++	+++	+/-	64.0
H72C	++	+	+	-	63.3
A73C	++	-	+	-	58.2
H72C-A558C	++	++	++	-	ND
H72C-P559C	-	-	-	-	63.3
H72C-E560C	+++	++	+++	-	ND
H72C-A561C	+++	+++	+++	-	65.3
H72C-Q562C	++	-	+	-	ND
H72C-Q563C	+++	+++	+++	-	65.1
**H72C-H564C**	+++	+++	+++	-	65.6
H72C-L565C	+++	+++	+++	-	66.0
H72C-L566C	+++	+	+	-	63.8
H72C-K567C	+++	+++	+++	-	65.6
A73C-A558C	+++	++	+++	-	65.6
A73C-P559C	+	-	-	-	ND
A73C-E560C	+++	++	+++	-	65.3
**A73C-A561C**	+++	+++	+++	-	66.1
A73C-Q562C	+++	-	+	-	ND
A73C-Q563C	+++	+++	+++	-	64.2
A73C-H564C	+++	++	+++	-	65.3
A73C-L565C	+++	+++	+++	-	65.6
A73C-L566C	+++	++	+++	-	65.8
A73C-K567C	++	+	+	-	ND
A73C-L568C	+++	+++	+++	-	63.8
A73C-T569C	+++	+++	+++	-	65.1

Figure S2. Trimers with two disulfide bonds linking gp120 and gp41. Related to Fig. 1. (**A**) Unpurified His-tagged BG505 Env proteins that include the original SOS bond (Cys501-Cys605; i.e. SOSIP.664) or one of the new H72C-H564C or A73C-A561C bonds, instead of or in addition to the SOS bond, were assessed for cleavage and trimerization efficiency by reducing SDS-PAGE (left panel) and BN-PAGE (right panel). All of the new mutants were properly cleaved, but their trimerization was less efficient than for SOSIP.664. (**B**) The antigenic phenotype of the same unpurified variants was assessed by ELISA. The 2G12, VRC01, PGT145 and PGT151 bNAbs each bound strongly to all the variants, similar to SOSIP.664. The trimers containing only the H72C-H564C or the A73C-A561C disulfide bond (i.e., not the SOS bond) were less reactive with the CD4i non-NAb 17b, but more reactive gp41 non-NAb F240 (see Fig. S1). The mutants with two inter-subunit disulfide bonds had a phenotype reflecting the presence of each bond. Thus, 17b binding was not detected, a property shared with the H72C-H564C and A73C-A561C single disulfide bond mutants, while F240 binding was also very low, a property conferred by the SOS bond. (**C**) Additional Cys substitutions at most of the residues between 558 and 569 were screened for their ability to form disulfide bonds with Cys72 and Cys73, in mutants that also contained the SOS bond. The various unpurified mutant proteins were tested for 2G12, PGT145 and PGT151 reactivity and for thermostability, as described in Fig. S1. Several inter-subunit disulfide bonds could form with no apparent adverse influence on the overall conformation of the trimer, suggesting that the 558-569 region of gp41 is conformationally flexible. Thus, in general, the mutants bound quaternary structure-dependent bNAbs to a similar or higher extent than the original SOSIP.664 trimer, and their thermostability was higher, which is presumably attributable to the additional stabilizing effect of the second inter-subunit disulfide bond in the trimer core. Color code: red, no binding; orange, moderate binding; green, strong binding. Antibody binding is also scaled from no reactivity (-) to strong binding (+++). ND: Not determined. The data are based on 3 experiments.

Figure S3

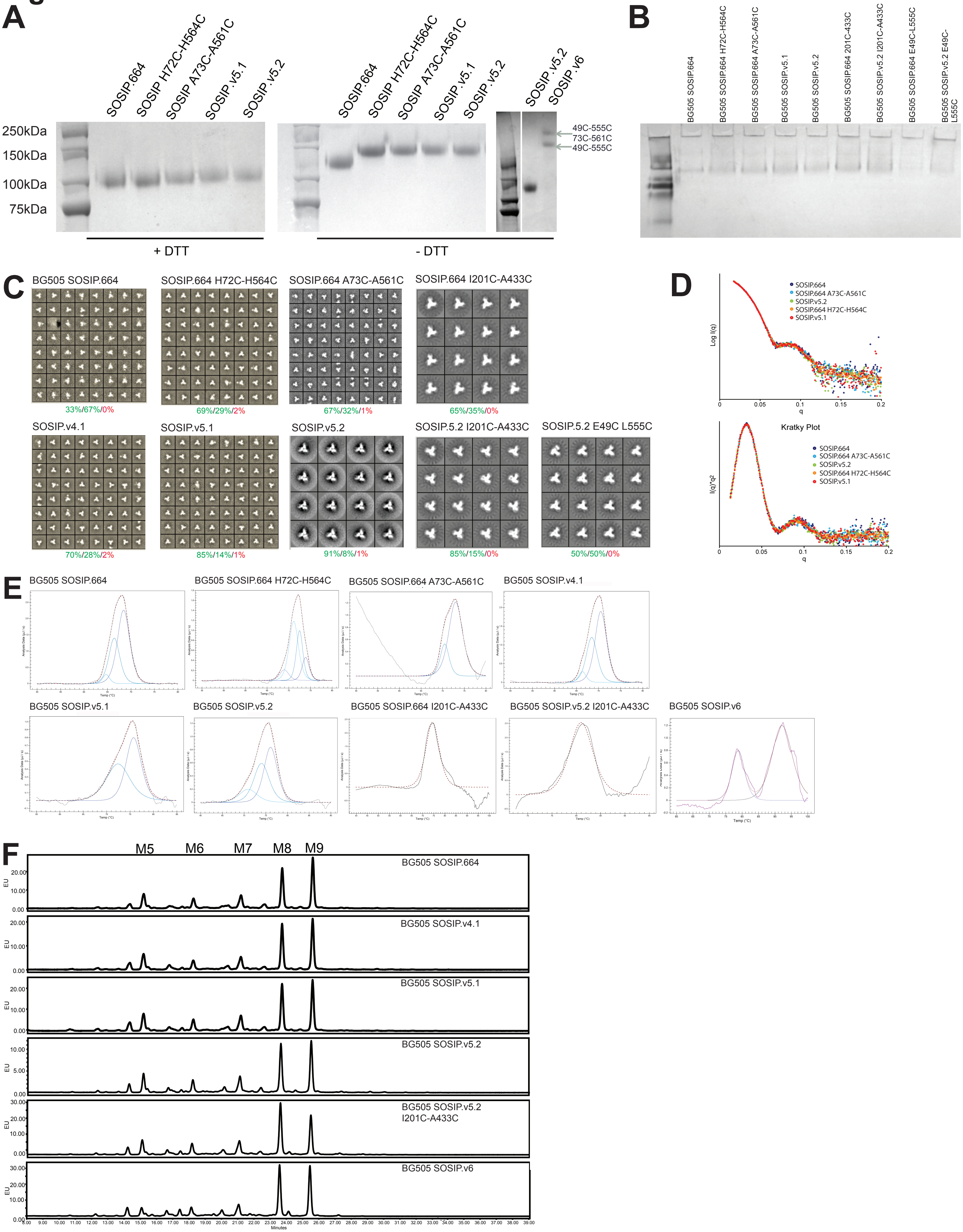

A
SOSIP.664
SOSIP H72C-H564C
SOSIP A73C-A561C
SOSIP.v5.1
SOSIP.v5.2
SOSIP.v6
250kDa
150kDa
100kDa
75kDa
49C-555C
73C-561C
49C-555C
+ DTT
- DTT
B
BG505 SOSIP.664
BG505 SOSIP.664 H72C-H564C
BG505 SOSIP.664 A73C-A561C
BG505 SOSIP.v5.1
BG505 SOSIP.v5.2
BG505 SOSIP.664 201C-433C
BG505 SOSIP.v5.2 I201C-A433C
BG505 SOSIP.664 E49C-L555C
BG505 SOSIP.v5.2 E49C-L555C
C
BG505 SOSIP.664
SOSIP.664 H72C-H564C
SOSIP.664 A73C-A561C
SOSIP.664 I201C-A433C
33%/67%/0%
69%/29%/2%
67%/32%/1%
65%/35%/0%
SOSIP.v4.1
SOSIP.v5.1
SOSIP.v5.2
SOSIP.5.2 I201C-A433C
SOSIP.5.2 E49C L555C
70%/28%/2%
85%/14%/1%
91%/8%/1%
85%/15%/0%
50%/50%/0%
D
Log I(q)
q
SOSIP.664
SOSIP.664 A73C-A561C
SOSIP.v5.2
SOSIP.664 H72C-H564C
SOSIP.v5.1
Kratky Plot
I(q)*q2
E
BG505 SOSIP.664
BG505 SOSIP.664 H72C-H564C
BG505 SOSIP.664 A73C-A561C
BG505 SOSIP.v4.1
BG505 SOSIP.v5.1
BG505 SOSIP.v5.2
BG505 SOSIP.664 I201C-A433C
BG505 SOSIP.v5.2 I201C-A433C
BG505 SOSIP.v6
F
M5
M6
M7
M8
M9
BG505 SOSIP.664
BG505 SOSIP.v4.1
BG505 SOSIP.v5.1
BG505 SOSIP.v5.2
BG505 SOSIP.v5.2 I201C-A433C
BG505 SOSIP.v6
EU
Minutes

Figure S3. Biochemical, biophysical and antigenic characterization of PGT145-purified stabilized BG505 SOSIP trimers. Related to Table 1 and Table 2 (**A**) Cleavage efficiency and disulfide bond formation was determined by SDS-PAGE under reducing and non-reducing conditions, respectively. Under reducing conditions, all proteins showed a gp120 band, indicating that they were completely cleaved. Under non-reducing conditions, the proteins all showed a gp140 band, but they migrated differently through the gels, indicative of different levels of compactness (compact proteins take up less SDS and therefore migrate slower through SDS-PAGE gels). The double disulfide bond variants all showed a slower migration pattern due to the presence of the extra bond. (**B**) The purified His-tagged BG505 SOSIP.664, SOSIP.v4.1 and SOSIP.v5 proteins were exclusively trimeric as determined by BN-PAGE. (**C**) The presence of native-like trimers in PGT145-purified preparations was assessed by NS-EM. The 2D reference-free class averages for the two double disulfide bond proteins (SOSIP.v5.1 and SOSIP.v5.2) are compared to SOSIP.664 and SOSIP.v4.1. The percentages of closed native-like and open native-like trimers (Pugach et al., 2015) are shown in green and the percentage of non-native trimers in red. (**D**) SAXS scattering curves (left panel) and Kratky plots (right panel) show the consistency of the scattering pattern among the various BG505 trimer mutants, indicating that they all have a similar overall structure in solution. (**E**) The thermal stabilities of BG505 SOSIP.664, SOSIP.v4.1 and SOSIP.v5 trimers were measured by DSC. The independent non-two state best-fit curves are depicted by a dashed red line and the T_m values of each peak are given in the graphs. See the Supplemental Experimental Procedures section for more details on curve modeling. (**F**) Glycan profiles of trimer variants were determined by HILIC-UPLC. $Man_{5\text{-}9}GlcNAc_2$ glycans are indicated as M5-M9 above the panel.

Figure S4

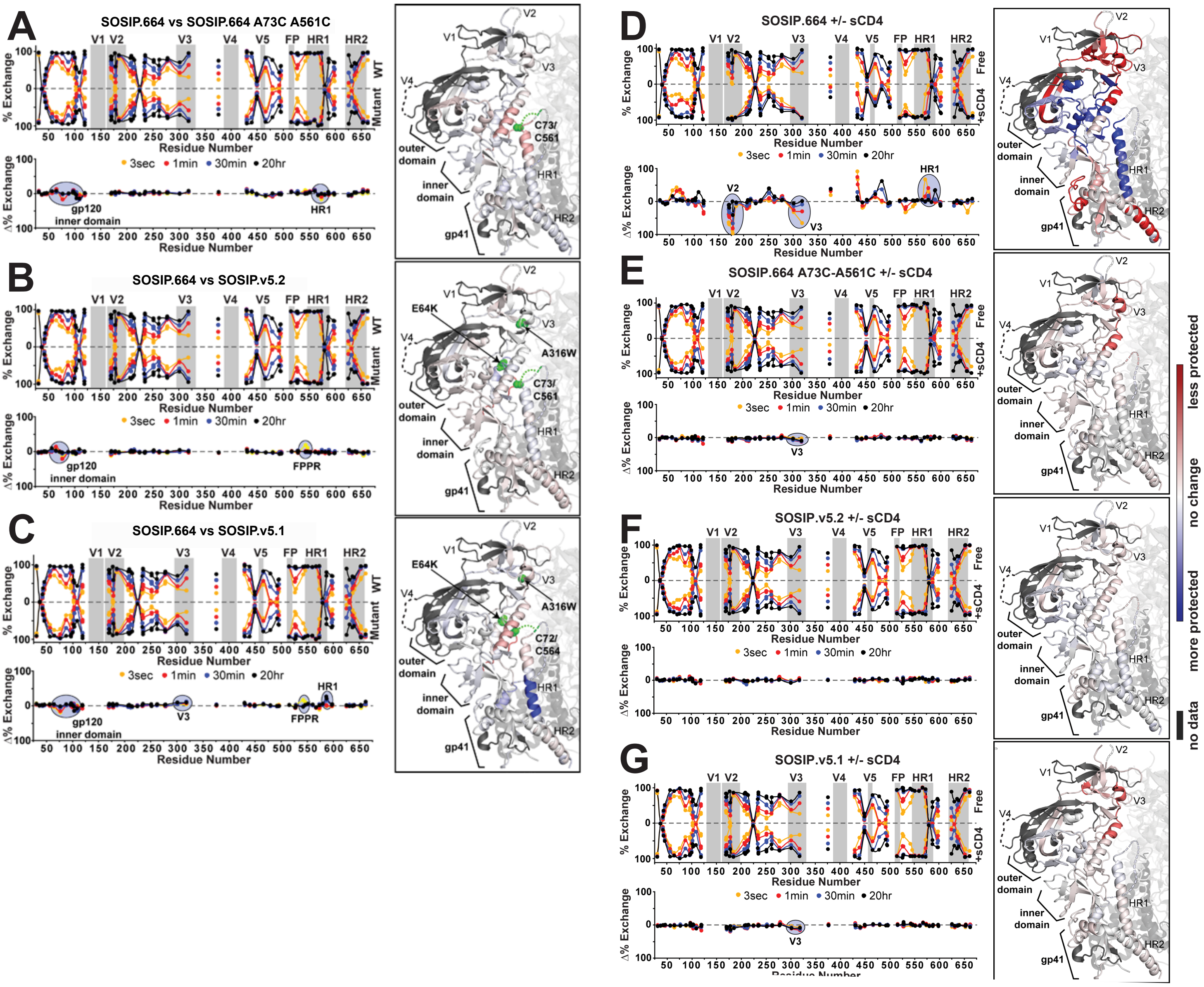
A
SOSIP.664 vs SOSIP.664 A73C A561C
B
SOSIP.664 vs SOSIP.v5.2
C
SOSIP.664 vs SOSIP.v5.1
D
SOSIP.664 +/- sCD4
E
SOSIP.664 A73C-A561C +/- sCD4
F
SOSIP.v5.2 +/- sCD4
G
SOSIP.v5.1 +/- sCD4
V1
V2
V3
V4
V5
FP
HR1
HR2
WT
Mutant
Free
+sCD4
% Exchange
Δ% Exchange
Residue Number
3sec
1min
30min
20hr
gp120 inner domain
FPPR
E64K
A316W
C73/ C561
C72/ C564
outer domain
inner domain
gp41
less protected
no change
more protected
no data

Figure S4. HDX-MS profiles of PGT145 purified wild type and stabilized BG505 SOSIP trimers. Related to Fig. 1. Butterfly plots comparing the HDX-MS profiles of various purified BG505 trimers. (**A**) SOSIP.664 A73C-A561C; (**B**) SOSIP.v5.2; (**C**) SOSIP.v5.1. (**D-F**) the same trimers as in **A-C** but with sCD4 present. The percent exchange for each observable peptide is plotted at the position of the center of the peptide on the primary sequence for each time point (3 s to 20 h). The difference plots below each primary plot reveal regions undergoing slower exchange (more protected, above the zero) and faster exchange (less protected, below the zero). Differences are mapped onto one lobe of the trimer crystal structure (PDB: 4VTP, Pancera et al., 2014). Segments unresolved in the crystal structure (V2, V4, and a portion of gp41) are shown as dashed lines. Point mutations are indicated and shown as green spheres.

Figure S5

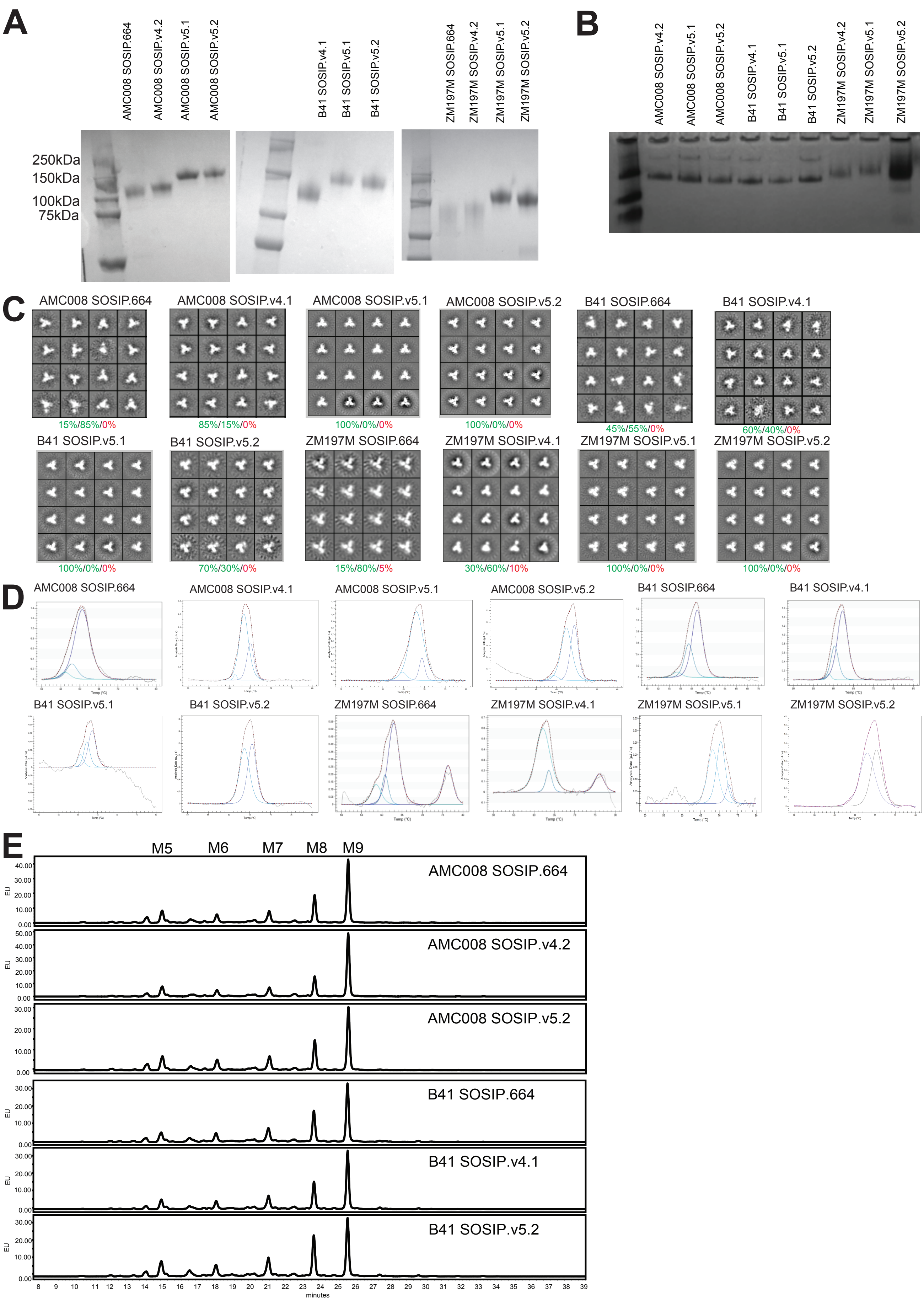
A
AMC008 SOSIP.664
AMC008 SOSIP.v4.2
AMC008 SOSIP.v5.1
AMC008 SOSIP.v5.2
B41 SOSIP.v4.1
B41 SOSIP.v5.1
B41 SOSIP.v5.2
ZM197M SOSIP.664
ZM197M SOSIP.v4.2
ZM197M SOSIP.v5.1
ZM197M SOSIP.v5.2
250kDa
150kDa
100kDa
75kDa
B
AMC008 SOSIP.v4.2
AMC008 SOSIP.v5.1
AMC008 SOSIP.v5.2
B41 SOSIP.v4.1
B41 SOSIP.v5.1
B41 SOSIP.v5.2
ZM197M SOSIP.v4.2
ZM197M SOSIP.v5.1
ZM197M SOSIP.v5.2
C
AMC008 SOSIP.664
15%/85%/0%
AMC008 SOSIP.v4.1
85%/15%/0%
AMC008 SOSIP.v5.1
100%/0%/0%
AMC008 SOSIP.v5.2
100%/0%/0%
B41 SOSIP.664
45%/55%/0%
B41 SOSIP.v4.1
60%/40%/0%
B41 SOSIP.v5.1
100%/0%/0%
B41 SOSIP.v5.2
70%/30%/0%
ZM197M SOSIP.664
15%/80%/5%
ZM197M SOSIP.v4.1
30%/60%/10%
ZM197M SOSIP.v5.1
100%/0%/0%
ZM197M SOSIP.v5.2
100%/0%/0%
D
AMC008 SOSIP.664
AMC008 SOSIP.v4.1
AMC008 SOSIP.v5.1
AMC008 SOSIP.v5.2
B41 SOSIP.664
B41 SOSIP.v4.1
B41 SOSIP.v5.1
B41 SOSIP.v5.2
ZM197M SOSIP.664
ZM197M SOSIP.v4.1
ZM197M SOSIP.v5.1
ZM197M SOSIP.v5.2
E
M5
M6
M7
M8
M9
AMC008 SOSIP.664
AMC008 SOSIP.v4.2
AMC008 SOSIP.v5.2
B41 SOSIP.664
B41 SOSIP.v4.1
B41 SOSIP.v5.2
EU
minutes
8 9 10 11 12 13 14 15 16 17 18 19 20 21 22 23 24 25 26 27 28 29 30 31 32 33 34 35 36 37 38 39

Figure S5. Biochemical biophysical and antigenic characterization of PGT145-purified stabilized clade-B (AMC008 and B41) and clade C (ZM197M) SOSIP.664 trimer variants. Related to Table 1 and Table 2. (**A**) Cleavage efficiency and disulfide bond formation was determined by SDS-PAGE under reducing and non-reducing conditions, respectively. Under reducing conditions, all proteins yielded a gp120 band, indicating that they were completely cleaved. Under non-reducing conditions the proteins all migrated as a gp140 band, but the mutants containing two disulfide bonds migrated more slowly through the gels. (**B**) The various subtype B (AMC008 and B41) and subtype C (ZM197M) proteins, purified by PGT145 affinity chromatography, were exclusively trimeric as determined by BN-PAGE. (**C**) The formation of native-like trimers was assessed by NS-EM. The 2D reference free class averages for the two double disulfide bond proteins (SOSIP.v5) compared to SOSIP.664 are shown. The percentages of closed native-like and open native-like trimers (Pugach et al., 2015) are shown in green and the percentage of non-native trimers in red. (**D**) Thermal stability was measured by DSC for the various D7324-tagged AMC008, B41 and ZM197M trimers. The independent non-two state best-fit curves are depicted in dashed red line and the T_m values of each peak are given in the graphs. See the Materials and Methods section for more details on curve modeling. (**E**) Glycan profiles of AMC008, B41 and ZM197M trimer variants were determined by HILIC-UPLC. $Man_{5\text{-}9}GlcNAc_2$ glycans are indicated as M5-M9 above the panel.

Table S1. Nomenclature of SOSIP trimers. Modifications present in each SOSIP trimer design are in green, with red indicating their absence. Related to Fig. 1.

			A501C-T605C	I559P	R6	MPER	I535M	L543Q	L543N	A316W	E64K	H66R	H72C-H564C	A73C-A561C	E49C-L555C
**SOSIP.681**	SOSIP.v1	SOSIP.v1	green	green	green	green	red	red	red	red	red	red	red	red	red
**SOSIP.664**	SOSIP.v2	SOSIP.v2	green	green	green	red	red	red	red	red	red	red	red	red	red
	**SOSIP.v3**	SOSIP.v3.1	green	green	green	red	green	green	red	red	red	red	red	red	red
		SOSIP.v3.2	green	green	green	red	green	red	green	red	red	red	red	red	red
	**SOSIP.v4**[a]	SOSIP.v4.1	green	green	green	red	green	green	green	green	green	red	red	red	red
		SOSIP.v4.2	green	green	green	red	green	green	green	green	red	green	red	red	red
	**SOSIP.v5**	SOSIP.v5.1	green	green	green	red	green	green	green	green	green	green	green	red	red
		SOSIP.v5.2	green	green	green	red	green	green	green	green	green	green	red	green	red
	**SOSIP.v6**	SOSIP.v6	green	green	green	red	green	green	green	green	green	green	red	green	green

[a] Described in de Taeye et al., 2015.

Table S2. Biophysical properties of BG505 SOSIP.664 trimers. Related to Fig. 1 and Table 1.

	Introduced mutations/ SOSIP version	**DLS** [a]		**SAXS** [a]	
		R_h (Å)[a]	Pd (%)	Rg (Å) (Guinier) [b]	Rg (Å) (GNOM) [c]
BG505	SOSIP.664	69.2	2.6	52.8	52.5
	SOSIP.664 H72C-H564C	ND	ND	ND	ND
	SOSIP.664 A73C-A561C	68.8	2.0	52.8	51.7
	SOSIP.v4.1	68.7	3.3	53.0	51.9
	SOSIP.v5.1	67.8	3.6	52.1	51.7
	SOSIP.v5.2	67.2	4.4	54.6	51.8

[a] DLS and SAXS analysis were performed in stabilized SOSIP trimer variants.

[b] Standard deviation differs in +/- 0.3-0.5Å.

[c] Standard deviation is +/- 0.2.

Table S3. X-ray data collection and refinement statistics. Related to Fig. 1.

	BG505 SOSIP.664 72C-564C + PGT122 + 35O22 + NIH45-46
Beamline	APS 23-ID-D
# Crystals	1
Wavelength, Å	1.03317
Space group	C2
Unit cell a, b, c (Å)	361.8, 215.9, 176.6
α, β, γ (°)	90, 114.0, 90
Resolution (Å)	40.0 – 7.0 (7.1 – 7.0)
Completeness	99.3 (99.9)
Redundancy	7.6 (8.1)
No. total reflections	149,758
No. unique reflections	19,551
I/σ	7.2 (1.7)
$CC_{1/2}$	99.4 (44.8)
R_{sym}	0.20 (0.88)
R_{pim}	0.08 (0.33)
Resolution (Å)	40.0 – 7.0
No. reflections total/R_{free}	19,539/978
R_{cryst}/R_{free}	0.33/0.34
RMSD bond length (Å)	0.02
RMSD bond angles (°)	1.2
Protein atoms/Glycan atoms	42,237
Wilson B-value ($Å^2$)	278
Overall average B-value ($Å^2$)	367
Ramachandran allowed %	99.0
MolProbity all-atom clashscore	10.4
PDB ID	5WDU

Table S5. Disulfide bond formation in BG505 SOSIP variants. Related to Fig. 1 and Table 1. Location of disulfide bonds was assessed by MS-MS-UPLC. CS is Charged State. NA is not applicable.

Disulfide Loop Domain	Disulfide-Linked Peptides	BG505 SOSIP.664 His tag				
		CS	Theoretical m/z	Experimental m/z	Mass Error (ppm)	MS/MS
I	DAETTLFC^{54}ASDAK<br>HNVWATHAC^{74}VPTDPNPQEIHLED^{88}VTEEFNMWK	3+	1719.4427	1719.4495	4	
		4+	1289.8338	1289.8410	6	
		5+	1032.0685	1032.0750	6	✓
		6+	860.2250	860.2331	10	
		7+	737.4796	737.4832	5	
II	NNMVEQMHTDIISLWDQSLKPC^{119}VK<br>LINC^{196}D^{197}TSAITQAC^{205}PK<br>LTPLC^{126}VTLQC^{131}TD^{132}VTND^{136}ITDDMR<br>D^{156}C^{157}SFD^{160}MTTELR	7+	1169.8301	1169.8358	5	
		8+	1023.7272	1023.7337	6	✓
		9+	910.0917	910.0940	3	
		10+	819.1832	819.1869	5	
III	C^{228}K<br>FN^{234}GTGPC^{239}PSVSTVQC^{247}THGIKPVVSTQLLLN^{262}GSLAEEEVMIR<br>VSFEPIPIHYC^{218}APAGFAILK	5+	1346.6868	1346.6867	0.1	
		6+	1122.4069	1122.4067	0.2	
		7+	962.2069	962.2082	1	✓
		8+	842.0570	842.0570	0	
IV	NILVQFNTPVQID^{295}C^{296}TRPND^{301}NTR<br>QAHC^{331}D^{332}VSK	2+	1721.8332	1721.8347	1	
		3+	1148.2245	1148.2295	4	
		4+	861.4202	861.4246	5	✓
		5+	689.3376	689.3425	7	
		6+	574.6159	574.6182	4	
V	FADSSGGDLEVTTHSFNC^{378}GGEFFYC^{385}DTSGLFDSTWISDTSVQGSDSTGSDDSITLPC^{418}R<br>C^{445}VSDITGLILTR	4+	1852.5669	1852.5723	3	
		5+	1482.2549	1482.2632	6	✓
		6+	1235.3803	1235.3881	6	
gp41 SOSIP	C^{501}K<br>LIC^{604}C^{605}TNVPWN^{611}SSWSNR<br>DQQLLGIWGC^{598}SGK	3+	1176.8808	1176.8860	4	
		4+	882.9124	882.9167	5	
		5+	706.5314	706.5352	5	✓
		6+	588.9440	588.9460	3	

Disulfide Loop Domain	Disulfide-Linked Peptides	BG505 SOSIP.v5.2 His tag				
		CS	Theoretical m/z	Experimental m/z	Mass Error (ppm)	MS/MS
I	DAETTLFC^{54}ASDAK<br>HNVWATHAC^{73}C^{74}VPTDPNPQEIHLED^{88}VTEEFNMWK<br>APEC^{561}QQHLLK	4+	1588.4748	1588.4701	3	
		5+	1270.9813	1270.9789	2	
		6+	1059.3189	1059.3171	2	✓
		7+	908.1316	908.1304	1	
		8+	794.7410	794.7401	1	
II	NNMVEQMHTDIISLWDQSLKPC^{119}VK*<br>LINC^{196}D^{197}TSAITQAC^{205}PK<br>LTPLC^{126}VTLQC^{131}TN^{132}VTND^{136}ITDDMR*<br>D^{156}C^{157}SFD^{160}MTTELR*	6+	1375.1331	1375.1414	6	
		7+	1178.8294	1178.8367	6	
		8+	1031.6017	1031.6061	4	✓
		9+	917.0912	917.0961	5	
		10+	825.4828	825.4881	6	
III	C^{228}K<br>FN^{234}GTGPC^{239}PSVSTVQC^{247}THGIKPVVSTQLLLN^{262}GSLAEEEVMIR<br>VSFEPIPIHYC^{218}APAGFAILK	5+	1346.6868	1346.6826	3	
		6+	1122.4069	1122.4022	4	
		7+	962.2069	962.2042	3	✓
		8+	842.0570	842.0552	2	
		9+	748.6070	748.6050	3	
IV	NILVQFNTPVQID^{295}C^{296}TRPND^{301}NTR<br>QAHC^{331}D^{332}VSK	2+	1721.8332	1721.8383	3	
		3+	1148.2245	1148.2256	1	
		4+	861.4202	861.4220	3	✓
		5+	689.3376	689.3415	6	
		6+	574.6159	574.6183	4	
V	FADSSGGDLEVTTHSFNC^{378}GGEFFYC^{385}DTSGLFDSTWISDTSVQGSDSTGSDDSITLPC^{418}R<br>C^{445}VSDITGLILTR	4+	1852.5669	1852.5714	3	
		5+	1482.2549	1482.2606	4	✓
		6+	1235.3803	1235.3863	5	
gp41 SOSIP	C^{501}K<br>LIC^{604}C^{605}TNVPWN^{611}SSWSNR<br>DQQLLGIWGC^{598}SGK	3+	1176.8808	1176.8824	1	
		4+	882.9124	882.9155	4	
		5+	706.5314	706.5343	4	✓
		6+	588.9440	588.9454	2	

Disulfide Loop Domain	Disulfide-Linked Peptides	BG505 SOSIP.v5.2 I201C-A433C His tag				
		CS	Theoretical *m/z*	Experimental *m/z*	Mass Error (ppm)	MS/MS
I	DAETTLFC^{54}ASDAK<br>HNVWATHAC^{73}C^{74}VPTDPNPQEIHLED^{88}VTEEFNMWK<br>APEC^{561}QQHLLK	4+	1588.4748	1588.4761	1	✓
		5+	1270.9813	1270.9756	5	
		6+	1059.3189	1059.3146	4	
		7+	908.1316	908.1285	3	
		8+	794.7410	794.7391	2	
II	IGC^{433}GQAMYAPPIQGVIR* NNMVEQMHTDIISLWDQSLKPC^{119}VK*<br>LINC^{196}D^{197}TSAC^{201}TQAC^{205}PK<br>LTPLC^{126}VTLQC^{131}TN^{132}VTND^{136}ITDDMR*<br>D^{156}C^{157}SFD^{160}MTTELR*	7+	1414.5061	1414.5113	6	✓
		8+	1237.8187	1237.8262	4	
		9+	1100.3952	1100.3992	5	
		10+	990.4564	990.4588	6	
III	C^{228}K<br>FN^{234}GTGPC^{239}PSVSTVQC^{247}THGIKPVVSTQLLLN^{262}GSLAEEEVMIR<br>VSFEPIPIHYC^{218}APAGFAILK	5+	1346.6868	1346.6850	3	✓
		6+	1122.4069	1122.4051	4	
		7+	962.2069	962.2031	3	
		8+	842.0570	842.0570	2	
IV	NILVQFNTPVQID^{295}C^{296}TRPND^{301}NTR<br>QAHC^{331}D^{332}VSK	3+	1148.2245	1148.2255	1	✓
		4+	861.4202	861.4207	1	
		5+	689.3376	689.3392	2	
		6+	574.6159	574.6169	2	
V	FADSSGGDLEVTTHSFNC^{378}GGEFFYC^{385}DTSGLFDSTWISDTSVQGSDSTGSDDSITLPC^{418}R<br>C^{445}VSDITGLILTR	4+	1852.5669	1852.5686	1	✓
		5+	1482.2549	1482.2595	3	
		6+	1235.3803	1235.3865	5	
gp41 SOSIP	C^{501}K<br>LIC^{604}C^{605}TNVPWN^{611}SSWSNR<br>DQQLLGIWGC^{598}SGK	3+	1176.8808	1176.8805	0.3	✓
		4+	882.9124	882.9136	1	
		5+	706.5314	706.5323	1	
		6+	588.9440	588.9447	1	

Disulfide Loop Domain	Disulfide-Linked Peptides	BG505 SOSIP.v6 His tag				
		CS	Theoretical *m/z*	Experimental *m/z*	Mass Error (ppm)	MS/MS
I	NLLSGIVQQSNC^{555}LR<br>DAC^{49}TTLFC^{54}ASDAK<br>HNVWATHAC^{73}C^{74}VPTDPNPQEIHLED^{88}VTEEFNMWK<br>APEC^{561}QQHLLK	6+	1336.1194	1336.1263	5	✓
		7+	1145.3891	1145.3919	3	
		8+	1002.3413	1002.3442	3	
		9+	891.0820	891.0833	2	
		10+	802.0745	802.0776	4	
II	NNMVEQMHTDIISLWDQSLKPC^{119}VK*<br>LINC^{196}D^{197}TSAITQAC^{205}PK<br>LTPLC^{126}VTLQC^{131}TD^{132}VTND^{136}ITDDMR*<br>D^{156}C^{157}SFD^{160}MTTELR*	6+	1375.2971	1375.3055	6	✓
		7+	1178.9700	1178.9772	6	
		8+	1031.7247	1031.7313	6	
		9+	917.2005	917.2061	6	
		10+	825.5812	825.5874	8	
III	C^{228}K<br>FN^{234}GTGPC^{239}PSVSTVQC^{247}THGIKPVVSTQLLLN^{262}GSLAEEEVMIR<br>VSFEPIPIHYC^{218}APAGFAILK	5+	1346.6868	1346.6806	5	✓
		6+	1122.4069	1122.4022	4	
		7+	962.2069	962.2032	4	
		8+	842.0570	842.0535	4	
		9+	748.6070	748.6044	4	
IV	NILVQFNTPVQID^{295}C^{296}TRPND^{301}NTR<br>QAHC^{331}D^{332}VSK	3+	1148.2245	1148.2257	1	✓
		4+	861.4202	861.4220	2	
		5+	689.3376	689.3401	4	
		6+	574.6159	574.6174	3	
V	FADSSGGDLEVTTHSFNC^{378}GGEFFYC^{385}DTSGLFDSTWISDTSVQGSDSTGSDDSITLPC^{418}R<br>C^{445}VSDITGLILTR	4+	1852.5669	1852.5607	5	✓
		5+	1482.2549	1482.2617	4	
		6+	1235.3803	1235.3852	3	
gp41 SOSIP	C^{501}K<br>LIC^{604}C^{605}TNVPWN^{611}SSWSNR<br>DQQLLGIWGC^{598}SGK	3+	1176.8808	1176.8837	3	✓
		4+	882.9124	882.9151	3	
		5+	706.5314	706.5342	4	
		6+	588.9440	588.9458	3	

Disulfide Loop Domain	Disulfide-linked Peptides	BG505 SOSIP.664	BG505 SOSIP.v5.2	BG505 SOSIP.v5.2 I201C-A433C	BG505 SOSIP.v6
I	HNVWATHA$C^{73}C^{74}$VPTDPNPQEIHLED^{88}VTEEFNMWK	NA	Trace	✗	✗
I	DAC^{49}TTLFC^{54}ASDAK	NA	NA	NA	✓
II	LIN$C^{196}D^{197}$TSAITQAC^{205}PK	Trace	Trace	NA	✓
II	LTPLC^{126}VTLQC^{131}TD^{132}VTND^{136}ITDDMR	✓	Trace	Trace	✓
III	C^{228}K<br>VSFEPIPIHYC^{218}APAGFAILK	Trace	✗	✗	Trace
III	FD^{234}GTGPC^{239}PSVSTVQC^{247}THGIKPVVSTQLLLD^{262}GSLAEEEVMIR	✓	✗	Trace	✓
gp41 SOSIP	LI$C^{604}C^{605}$TNVPWN^{611}SSWSNR	✓	✓	✓	✓

Supplemental Experimental Procedures

Construct design

The constructs expressing BG505, B41, AMC008 and ZM1097M SOSIP.664 proteins have been described elsewhere (Julien et al., 2015; Pugach et al., 2015; Sanders et al., 2013; de Taeye et al., 2015). To improve the formation of soluble trimers, these constructs contained the following changes compared to the original Env sequence: a TPA signal sequence; A501C and T605C; I559P; REKR to RRRRRR at the C-terminus of gp120; a stop codon after residue 664 (Binley et al., 2000, 2002; Khayat et al., 2013; Klasse et al., 2013; Sanders et al., 2002). In addition, we introduced substitutions to restore glycan-dependent bNAb epitopes: T332N in BG505 and D156N, E295N and D332N in ZM197M (Julien et al., 2015; Pugach et al., 2015; Sanders et al., 2013; de Taeye et al., 2015). We refer to these constructs as SOSIP.664. We further introduced the SOSIP.v4 mutations, i.e. E64K or H66R and A316W trimer-stabilizing changes (de Taeye et al., 2015), as well as the I/V535M and L543N trimer-improving changes to gp41, where necessary (de Taeye et al., 2015). We also constructed AMC008, B41 and ZM197M trimer variants bearing either a His-tag (BG505) or a D7324 epitope-tag sequence at the C-terminus after residue 664 (GSGSGGSGHHHHHHHH or GSAPTKAKRRVVQREKR, respectively), as described in Sanders et al. 2013. Point mutations were made using Quickchange site-directed mutagenesis kit (Agilent Technologies, La Jolla, CA, USA) and constructs were verified by sequencing prior to use.

Protein expression and purification

The Env proteins were transiently expressed in adherent 293T cells or suspension 293F cells in the presence of excess co-transfected *furin* and purified using PGT145-affinity chromatography as described previously (Julien et al., 2013a; Julien et al., 2015; Pugach et al., 2015; Sanders et al., 2013; de Taeye et al., 2015). All of the exploratory experiments using unpurified Env from supernatant of transfected cells made use of 293T cell-expressed proteins, while purified trimers were derived from 293F cells. SDS-PAGE and BN-PAGE analyses were performed as previously described (Julien et al., 2013a; Pugach et al., 2015; Sanders et al., 2013; de Taeye et al., 2015).

Neutralization assays

The TZM-bl cell line, which expresses high levels of CD4, CCR5 and CXCR4 and contains beta-galactosidase and luciferase genes under the control of the HIV-1 long terminal repeat promoter, was used for neutralization assays. We used Env-pseudotyped or chimeric molecular clone viruses to perform the neutralization assays at DUMC, Duke University Medical Center, AMC, Academic Medical Center, and WCMC, Weill Medical College of Cornell University. For methodology, see https://www.hiv.lanl.gov/content/nab-reference-strains/html/Protocol-for-Neutralizing-Antibody-Screening-Assay-for-HIV-1-in-TZMbl-cells_Jan2016.pdf; Sanders et al., 2013 and Klasse et al., 2016, respectively.

SDS-PAGE and Blue Native-PAGE

The presence of Env trimer was analyzed using SDS-PAGE and BN-PAGE followed by western blot or Coomassie blue dye staining according to previous protocols described in Sanders et al. 2002 and Schülke et al. 2002. In summary, the input material, Env-containing supernatant or purified trimer, was mixed with loading buffer (for SDS-PAGE: 25mM Tris, 192mM glycine, 20% v/v glycerol, 4% m/v SDS, 0.1% v/v bromophenol blue in milli-Q water; for Native-PAGE: 500μl 20x MOPS buffer, 1000μl 100% ultrapure glycerol, 50μl Coomassie brilliant blue and 600μl milli-Q water). For SDS-PAGE gels, the samples were incubated at 99°C for 5min with or without DTT (100mM) and 4-12% Tris-Glycine gels were run at 125V for 2h using running buffer (Invitrogen). For Native-PAGE, the samples were directly loaded onto a 4-12% Bis-Tris NuPAGE gel and the gel was run at 200V for 2h using Anode and Cathode-buffer (Invitrogen). Western Blot was performed using Arp3119 followed by HRP-labeled goat anti-mouse or 2G12 MAb (0.1μg/ml) followed by HRP-labeled goat anti-human IgG for SDS-PAGE and Native-PAGE, respectively. Coomassie blue staining was performed using PageBlue Protein Staining Solution (Thermo Scientific) and Colloidal Blue Staining Kit (Life Technologies) for SDS-PAGE and Native-PAGE, respectively.

Surface Plasmon Resonance (SPR), ELISA and thermostability ELISA

SPR analyses were performed with His-tagged trimers immobilized by anti-His antibody covalently linked to CM5 chips as previously described (Derking et al., 2015; Yasmeen et al., 2014).

D7324-capture and Ni-NTA capture ELISAs have been described elsewhere (Derking et al., 2015; Sanders et al., 2013). Briefly, for D7324-capture ELISA microloan-600, half-area plates (Greiner Bio-One) were coated overnight with D7324 antibody at 10μg/ml in 0.1M $NaHCO_3$, pH 8.6. After washing and blocking the D7324 antibody, the trimers were added at 2.2 μg/ml in TBS for 2h. After washing the unbound trimers with TBS, the different antibodies were added serially diluted. After washing with TBS, the HRP-labeled goat anti-human IgG (Jackson Immunoresearch) was added for 45 minutes. The colorimetric detection was performed with develop solution (1% 3,3',5,5'-tetramethylbenzidine, 0.01% H_2O_2, 100mM sodium acetate and 100mM citric acid) after five washes with TBS/0.05% tween-20). The colorimetric reaction was stopped with 0.8M H_2SO_4 when a plateau was reached. For Ni-NTA capture ELISA, Ni-HisSorb plates (Qiagen) were used. Trimers were directly added to the plate at a concentration of 0.6μg/ml. Detection was done using broadly neutralizing antibodies serially diluted.

ELISA-based thermostability ELISA was performed with unpurified BG505 His-tagged mutants as previously described (de Taeye et al. 2015). In short, 100μl of unpurified supernatants were incubated for 30 mins at different temperatures ranging between 39 and 77°C using a G-storm PCR machine (GRI Lab Care). Supernatants were transferred to a 96-well Ni-NTA plate and a Ni-NTA capture ELISA was performed following the described protocol above. The detection was done using the 2G12 antibody at 0.1μg/ml.

Differential scanning calorimetry (DSC)

To probe the thermostability of SOSIP.664 trimers, we used a Nano-DSC (TA Instruments, New Castle, DE, USA) and analyzed the data with NanoAnalyze Software v.3.3.0 (TA Instruments). The data were fitted using an independent non-two-state model, as the asymmetry of some of the peaks suggested the presence of unfolding intermediates. However, for simplification, we also analyzed the data using a two-state scaled model. We report the T_m values derived from the two-state scaled model in the main manuscript. All of the DSC data were derived using tagged trimers. When some comparative studies were performed, we found that the T_m values obtained with His-tagged trimers were consistently ~0.9-1.0°C higher than those for the same trimers without tag (data not shown), and 0.6°C higher than those for D7324-tagged trimers (de Taeye et al., 2015). For example, PGT145-purified His-tagged BG505 SOSIP.664 trimers have a T_m of 67.6°C, while the corresponding non-tagged trimers have a T_m of 66.7°C (data not shown), and the D7324-tagged versions have a T_m of 67.1°C (de Taeye et al., 2015). The implication is that the His-tag has a modest stabilizing effect on the trimer.

Analysis of total glycan profiles by HILIC-UPLC

N-linked glycans were enzymatically released from Env glycoproteins by in-gel digestion with Peptide-N-Glycosidase F (PNGase F), subsequently fluorescently labelled with 2-aminobenzoic acid (2-AA) and analyzed by HILIC-UPLC (Hydrophilic Interaction Liquid Chromatography – Ultra Performance Liquid Chromatography), as previously described (Behrens et al., 2016; Neville et al., 2009; Pritchard et al., 2015). Digestion of released glycans with Endoglycosidase H (Endo H) was used to determine the abundance of oligomannose-type glycans (Pritchard et al., 2015).

Negative-stain electron microscopy

Purified Env trimers were analyzed by negative-stain EM. A 3 μl aliquot containing ~0.03 mg/mL of the trimer was applied for 5 s onto a carbon-coated 400 Cu mesh grid that had been glow discharged at 20 mA for 30 s, then negatively stained with uranyl formate for 30 s. Data were collected using a FEI Tecnai F20 or T12 electron microscope operating at 120 keV, with an electron dose of ~55 $e^-/Å^2$ and a magnification of 52,000x that resulted in a pixel size of 2.05Å at the specimen plane. Images were acquired with a Gatan US4000 CCD or Tietz TemCam-F416 CMOS camera using a nominal defocus range of 900 to 1300 nm.

Image processing

Particles were picked automatically using DoG Picker and put into a particle stack using the Appion software package. Initial, reference-free, two-dimensional (2D) class averages were calculated using particles binned by five via the Xmipp Clustering 2D Alignment and sorted into classes. Particles

corresponding to trimers were selected into a substack and binned by four before another round of reference-free alignment was carried out using the Xmipp Clustering and 2D alignment and IMAGIC software systems.

Small Angle X-ray Scattering (SAXS)

SAXS measurements were conducted on Beam Line 4-2 at the Stanford Synchrotron Radiation Lightsource (Smolsky et al., 2007). The focused 11 keV X-ray beam irradiated a thin-wall quartz capillary cell, placed at 2.5 m upstream of the MX 225HE detector (Rayonix, Evanston, IL, USA). Aliquots containing 50 µl of BG505 constructs (1 – 2 mg/ml) were injected onto a high resolution Sepharose 200 column (GE Healthcare, Wilmington, MA, USA) with a flow rate of 50 µl/min in a buffer comprising 20 mM Na_3PO_4 pH 7.4, 150 mM NaCl, 0.02% NaN_3, 1 mM EDTA. The flow from the column passed through a UV detector cell and into the quartz capillary cell. Exposures of 1 s duration were collected every 5 s throughout the run, with a circulating water bath maintaining the capillary cell temperature at 8°C. The detector pixel numbers were radially integrated and scaled as described previously (de Taeye et al., 2015). The radius of gyration (R_g) and scattering at zero angle [I(0)] parameters for each frame were batch analyzed using autoRg, and frames with stable R_g values were merged *in primus* for the final scattering curve (Petoukhov et al., 2007). Real space distance distribution functions were calculated from the merged data sets using GNOM (Svergun, 1992).

Dynamic Light Scattering (DLS)

Proteins in PBS at 1 - 2 mg/ml were centrifuged at 15000g for 10 min at 4°C immediately before light scattering measurements. Data were collected on a Dynapro instrument (Wyatt Technologies, Goleta, CA, USA), with 30 acquisitions of 10 s each at 20°C, and analyzed with the manufacturer's software (Dynamics, Wyatt Technologies). We note that the hydrodynamic radius (R_h) of 6.9 nm derived using DLS for BG505 SOSIP.664 trimers that we report here and elsewhere (Table S2; de Taeye et al., 2015) is slightly lower than the previously reported value of 8.1 nm (Julien et al., 2013b). The latter value was derived using flow mode SEC coupled with static and quasi-elastic light scattering detectors, and fitted using a monomodal model. Calculated hydrodynamic radius values derived using this method represent the mean and distribution of diffusion constants.

X-ray crystallography

The BG505 SOSIP.664 72C-564C trimer was expressed and purified as previously described (Julien et al. 2013b). Purified trimers were mixed with a molar excess of the PGT122 Fab and 35O22 Fab and treated with EndoH (New England BioLabs). Subsequently, the complex was mixed with NIH45-46 scFv in molar excess and the quaternary complex was purified to size homogeneity using a Superose 6 10/30 gel filtration column (GE Healthcare). The complex was set up for crystallization trials at a protein concentration of 4 mg/ml, using an Oryx8 crystallization robot (Douglas Instruments). Crystals grew in sitting drop experiments from a condition containing 8% (w/v) polyethylene glycol 8000, 0.1 M Tris, pH 8.5. A complete dataset was obtained to 7.0 Å resolution from a single flash-cooled crystal that was cryo-protected with 30% glycerol. Data were processed using XDS (Kabsch, 2010), and data collection and processing statistics are reported in Table S3. For molecular replacement in PHASER (McCoy et al., 2007), a hybrid model was generated using the BG505 SOSIP.664 trimer + PGT122 Fab + 35022 Fab from PDB ID: 4TVP and NIH45-46 scFv superposed from PDB ID: 5D9Q. The resulting hybrid model was used as the search model. Iterative rigid body and grouped B-factor refinements were carried-out with non-crystallographic symmetry (NCS) in PHENIX and inspected in COOT (Adams et al., 2010; Emsley and Cowtan, 2004). Refinement statistics are summarized in Table S3.

Hydrogen-Deuterium Exchange (HD-X)

Soluble two-domain CD4 (2D-sCD4) (Garlick et al., 1990) was obtained from the NIH. All proteins were SEC-purified using a Superdex S200 column (GE Healthcare) and a PBS buffer (20 mM sodium phosphate pH 7.4, 150 mM NaCl, 1 mM EDTA, 0.02 % sodium azide) and concentrated to 1 mg/mL immediately before HDX-MS analysis. Complexes were formed by an overnight incubation at 4°C with 2D-sCD4, which was present at a 3-fold molar excess relative to each protomer of the trimer. Native gels were run for each sample to monitor sCD4-trimer complex formation. BG505 trimers (15 µg) were diluted 10-fold into deuterated PBS buffer at room temperature. After incubation periods of 3 s, 1 min, 30 min, and 20 h, the exchange reactions were quenched by mixing with an equal volume of cold 200 mM

TCEP, 0.2% formic acid (final pH 2.5). The samples were subsequently digested with pepsin (0.15 mg/mL) for 5 min on ice, flash frozen in liquid nitrogen and stored at -80°C. Differences in deuterium exchange profiles that exceeded the error of the measurement were visualized on the trimer structure (PDB: 4TVP) using custom macros in PyMOL (DeLano, 2002).

Analysis of disulfide bond patterns in BG505 Env trimers

Disulfide bond patterns of BG505 Env trimers were determined by mapping the disulfide-linked peptides by mass spectrometry. Details of the sample preparation were described previously (Go et al., 2011, 2014). Briefly, about 20 μg of Env samples were alkylated with a 10-fold molar excess of 4-vinylpyridine in the dark for one hour at room temperature to cap free cysteine residues. Alkylated Env samples were subsequently deglycosylated with 500 U of PNGase F in 100 μL of 100 mM ammonium citrate buffer (pH 6.5) for one week at 37°C. The fully deglycosylated and alkylated samples were digested overnight with trypsin (protein to enzyme ratio of 30:1) at 37°C and were analyzed by LC-MS using an Orbitrap Velos Pro™ hybrid (Thermo Scientific, San Jose CA) mass spectrometer equipped with electron transfer dissociation (ETD) module coupled to an Acquity Ultra Performance Liquid Chromatography (UPLC®) system (Waters, Milford MA). About 5 μL (1 μg equivalent) of the tryptic digest was injected onto a C18 PepMap™ 300 column (300 μm i.d. x 15 cm, 300 Å; Thermo Scientific, Sunnyvale, CA) and the peptides were separated using a linear gradient starting from 3% B to 40% B gradient in 50 min, then 90% B in 10 min, and re-equilibration at 97% A for 10 min. LC-MS runs were performed with a flow rate of 5 μL/min using mobile phases consisting of solvent A: 99.9% HPLC-grade H_2O + 0.1% formic acid and solvent B: 99.9 % HPLC grade CH_3CN + 0.1% formic acid. Data were collected using the data-dependent mode with five most intense ions in a high resolution scan in the Orbitrap were subjected to alternating collision-induced dissociation (CID) and ETD in the linear ion trap to determine the disulfide connectivity. Data analysis was performed using Mascot search engine for peptides containing free cysteine residues and disulfide bond patterns were analyzed manually as described previously (Go et al., 2011, 2014).

Immunizations

Rabbits were immunized at week 0, 4 and 20 with 22 μg of Env trimer or gp120. Rabbit sera was taken at week 22 and assayed for autologous and cross-reactive antibody responses using the TZM-bl cell neutralization assay. Binding titers were assessed by performing D7324 capture ELISA with week 22 sera as described elsewhere (Sanders et al., 2013). The tier classification of the Env pseudotyped viruses have been described in deCamp et al., 2014.

Supplemental References

Adams, P.D., Afonine, P. V., Bunkóczi, G., Chen, V.B., Davis, I.W., Echols, N., Headd, J.J., Hung, L.W., Kapral, G.J., Grosse-Kunstleve, R.W., et al. (2010). PHENIX: A comprehensive Python-based system for macromolecular structure solution. Acta Crystallogr. Sect. D Biol. Crystallogr. *66*, 213–221.

Behrens, A.J., Vasiljevic, S., Pritchard, L.K., Harvey, D.J., Andev, R.S., Krumm, S.A., Struwe, W.B., Cupo, A., Kumar, A., Zitzmann, N., et al. (2016). Composition and antigenic effects of individual glycan sites of a trimeric HIV-1 envelope glycoprotein. Cell Rep. *14*, 2695–2706.

Binley, J.M., Sanders, R.W., Master, A., Cayanan, C.S., Wiley, C.L., Schiffner, L., Travis, B., Kuhmann, S., Burton, D.R., Hu, S.-L., et al. (2002). Enhancing the proteolytic maturation of human immunodeficiency virus type 1 envelope glycoproteins. J. Virol. *76*, 2606–2616.

Cavacini, L.A., Emes, C.L., Wisnewski, A. V, Power, J., Lewis, G., Montefiori, D., and Posner, M.R. (1998). Functional and molecular characterization of human monoclonal antibody reactive with the immunodominant region of HIV type 1 glycoprotein 41. AIDS Res. Hum. Retroviruses *14*, 1271–1280.

deCamp, A., Hraber, P., Bailer, R.T., Seaman, M.S., Ochsenbauer, C., Kappes, J., Gottardo, R., Edlefsen, P., Self, S., Tang, H., et al. (2014). Global panel of HIV-1 Env reference strains for standardized assessments of vaccine-elicited neutralizing antibodies. J. Virol. *88*, 2489–2507.

DeLano, W. (2002). Pymol: An open-source molecular graphics tool. CCP4 Newsl. Protein Crystallogr. *700*.

Emsley, P., and Cowtan, K. (2004). Coot: Model-building tools for molecular graphics. Acta Crystallogr. Sect. D Biol. Crystallogr. *60*, 2126–2132.

Garlick, R.L., Kirschner, R.J., Eckenrode, F.M., Tarpley, W.G., and Tomich, C.S. (1990). *Escherichia coli* expression, purification, and biological activity of a truncated soluble CD4. AIDS Res. Hum. Retroviruses *6*, 465–479.

Go, E.P., Zhang, Y., Menon, S., and Desaire, H. (2011). Analysis of the disulfide bond arrangement of the HIV-1 envelope protein CON-S gp140 ΔCFI shows variability in the V1 and V2 regions. J. Proteome Res. *10*, 578–591.
Go, E.P., Hua, D., and Desaire, H. (2014). Glycosylation and disulfide bond analysis of transiently and stably expressed clade C HIV-1 gp140 trimers in 293T cells identifies disulfide heterogeneity present in both proteins and differences in o-linked glycosylation. J. Proteome Res. *13*, 4012–4027.
Kabsch, W. (2010). *XDS*. Acta Crystallogr. Sect. D Biol. Crystallogr. *66*, 125–132.
Khayat, R., Lee, J.H., Julien, J.-P., Cupo, A., Klasse, P.J., Sanders, R.W., Moore, J.P., Wilson, I. A., and Ward, A.B. (2013). Structural characterization of cleaved, soluble HIV-1 envelope glycoprotein trimers. J. Virol. *87*, 9865–9872.
Klasse, P.J., Depetris, R.S., Pejchal, R., Julien, J.-P., Khayat, R., Lee, J.H., Marozsan, A.J., Cupo, A., Cocco, N., Korzun, J., et al. (2013). Influences on trimerization and aggregation of soluble, cleaved HIV-1 SOSIP envelope glycoprotein. J. Virol. *87*, 9873–9885.
McCoy, A.J., Grosse-Kunstleve, R.W., Adams, P.D., Winn, M.D., Storoni, L.C., and Read, R.J. (2007). Phaser crystallographic software. J. Appl. Crystallogr. *40*, 658–674.
Neville, D.C.A., Dwek, R.A., and Butters, T.D. (2009). Development of a single column method for the separation of lipid- And protein-derived oligosaccharides. J. Proteome Res. *8*, 681–687.
Petoukhov, M. V., Konarev, P. V., Kikhney, A.G., and Svergun, D.I. (2007). ATSAS 2.1 - Towards automated and web-supported small-angle scattering data analysis. J. Appl. Crystallogr. *40*, 223–228.
Ringe, R.P., Sanders, R.W., Yasmeen, A., Kim, H.J., Lee, J.H., Cupo, A., Korzun, J., Derking, R., van Montfort, T., Julien, J.-P., et al. (2013). Cleavage strongly influences whether soluble HIV-1 envelope glycoprotein trimers adopt a native-like conformation. Proc. Natl. Acad. Sci. U. S. A. *110*, 18256–18261.
Schülke, N., Vesanen, M.S., Sanders, R.W., Lu, M., Anselma, D.J., Villa, A.R., Paul, W., Parren, H.I., Binley, J.M., Roux, K.H., et al. (2002). Oligomeric and conformational properties of a proteolytically mature , immunodeficiency virus type 1 gp140 envelope glycoprotein. J. Virol. *76*, 7760.
Smolsky, I.L., Liu, P., Niebuhr, M., Ito, K., Weiss, T.M., and Tsuruta, H. (2007). Biological small-angle X-ray scattering facility at the Stanford Synchrotron Radiation Laboratory. J. Appl. Crystallogr. *40*, 453–458.
Svergun, D.I. (1992). Determination of the regularization parameter in indirect-transform methods using perceptual criteria. J. Appl. Crystallogr. *25*, 495–503.
Yasmeen, A., Ringe, R., Derking, R., Cupo, A., Julien, J.P., Burton, D.R., Ward, A.B., Wilson, I.A., Sanders, R.W., Moore, J.P., et al. (2014). Differential binding of neutralizing and non-neutralizing antibodies to native-like soluble HIV-1 Env trimers, uncleaved Env proteins, and monomeric subunits. Retrovirology *11*, 41.
